# PRMT3 restricts porcine epidemic diarrhea virus replication by disrupting the interaction between VAPA and the viral nucleocapsid protein

**DOI:** 10.1371/journal.ppat.1014599

**Published:** 2026-09-08

**Authors:** He-Yong Wu, Shu-Yu Zhong, Ao-Si Qi, Zi-Ru Wang, Qiu-Se Tu, Feng Li, Rui Wu, Jing-Yao Wang, Fan Song, Tian-Yi Zhang, Ting Li, Qing-Chun Yu, Hong-Ming Yuan, Dong-Mei Lv, Hong-Sheng Ouyang, Xi-Liang Du, Da-Xin Pang, Zi-Cong Xie

**Affiliations:** 1 State Key Laboratory for Diagnosis and Treatment of Severe Zoonotic Infectious Diseases, Animal Genome Editing Technology Innovation Center, College of Animal Sciences, Jilin University, Changchun, Jilin, China; 2 The First Affiliated Hospital, Zhejiang University School of Medicine, Zhejiang University, Hangzhou, Zhejiang, China; 3 Chongqing Jitang Biotechnology Research Institute Co., Ltd., Chongqing, China; 4 State Key Laboratory for Zoonotic Diseases, Key Laboratory of Zoonosis Research, Ministry of Education, College of Veterinary Medicine, Jilin University, Changchun, Jilin, China; Yangzhou University, CHINA

## Abstract

Porcine epidemic diarrhea virus (PEDV) represents a severe threat to the global swine industry. Its infection process involves intricate virus–host interactions and immune evasion mechanisms, but effective therapeutic targets remain elusive. In this study, we identified protein arginine methyltransferase 3 (PRMT3) as a novel regulatory factor that significantly modulates PEDV infection via genome-wide CRISPR/Cas9 knockout library screening. Knockout or inhibition of PRMT3 markedly enhanced PEDV infection in multiple cell lines, including LLC-PK1, IPEC-J2, and primary porcine intestinal epithelial cells. Mechanistic investigations revealed that PRMT3 can restrict PEDV infection by interacting with vesicle-associated membrane protein-associated protein A (VAPA). Further analysis revealed that VAPA facilitates cholesterol transport through binding to oxysterol-binding protein (OSBP) and inhibits the autophagic degradation of the viral nucleocapsid (N) protein, with both processes being critical for promoting PEDV infection in host cells. A detailed analysis revealed that K52 within its major sperm protein (MSP) domain interacts with D404 and D405 in the two phenylalanines in an acidic tract (FFAT)-like motifs of the N protein, and these interactions proved essential for PEDV infection. In summary, this is the first study to identify and validate the PRMT3–VAPA–N protein autophagic degradation axis as a key pathway through which PRMT3 suppresses PEDV infection, with VAPA acting as an essential host factor for PEDV pathogenesis. These findings uncover novel signaling pathways and molecular targets for the development of anti-PEDV therapeutics.

## Author summary

Porcine epidemic diarrhea virus (PEDV) severely hampers global pig production, and few effective therapeutic targets have been identified. Using genome-wide CRISPR/Cas9 screening, we identified protein arginine methyltransferase 3 (PRMT3) as a novel anti-PEDV–host restriction factor. We found that PRMT3 knockout or inhibition markedly boosted PEDV replication in diverse porcine cell lines. Mechanically, we revealed that PRMT3 can restrict PEDV by interacting with the proviral factor vesicle-associated membrane protein-associated protein A (VAPA). We further demonstrated that VAPA promotes viral entry via oxysterol-binding protein-dependent cholesterol transport and suppresses autophagic degradation of the viral nucleocapsid (N) protein to facilitate replication, relying on the VAPA major sperm protein (MSP) domain and D404 in N protein for mutual binding. Our study establishes the critical PRMT3–VAPA–N protein–autophagy axis against PEDV and provides candidate targets for antiviral development.

## Introduction

Porcine epidemic diarrhea (PED) is a highly contagious intestinal disease caused by porcine epidemic diarrhea virus (PEDV), a member of the Coronaviridae family. PEDV causes severe enteric lesions in suckling piglets, with mortality rates ranging from 80% to 100% [1,2]. The continuous emergence of novel PEDV variants has complicated prevention and control efforts. Commercial PEDV vaccines are currently available, yet they exhibit obvious limitations: these vaccines cannot provide comprehensive cross-protection against continuously emerging PEDV variant strains and show insufficient protective efficacy, which hinders the prevention and control of PED. The PEDV genome contains seven open-reading frames encoding four structural proteins [spike, envelope, membrane, and nucleocapsid (N)], 16 nonstructural proteins (nsp1–nsp16), and the accessory protein ORF3 [3]. Among members of Coronaviridae, PEDV is unique in that it possesses an alkaline phosphorylated N protein, and this protein binds and packages viral genomic RNA into nucleocapsids, playing an indispensable role in viral particle assembly [4]. Several host restriction factors and signaling pathways have been found to regulate PEDV replication and infection in recent years, but few of these factors have been thoroughly characterized. The identification of key host factors that restrict viral infection, thereby representing potential drug targets, requires further in-depth investigation. Elucidating host factors and signaling pathways involved in PEDV infection is critical for developing innovative interventions against PED.

Protein arginine methylation, catalyzed by protein arginine methyltransferases (PRMTs), is a vital posttranslational modification that regulates diverse cellular signaling cascades. To date, nine PRMT isoforms (PRMT1–PRMT9) have been identified in mammals and classified into three subtypes. Type I PRMTs (PRMT1, PRMT2, PRMT3, PRMT4, PRMT6, and PRMT8) catalyze the conversion of monomethyl arginine (MMA) to asymmetric dimethylarginine [5]. Type II PRMTs (PRMT5 and PRMT9) mediate the formation of symmetric dimethylarginine from MMA, whereas the type III PRMT PRMT7 exclusively generates MMA [6]. PRMT3 is a unique member of the PRMT family characterized by a distinct structure and predominant cytoplasmic localization. Its N-terminal region contains a unique C2H2 zinc-finger motif and two N-terminal helices not found in other PRMTs, and its C-terminal catalytic domain is responsible for methyltransferase activity. Structural analysis of PRMT3 identified four critical functional motifs: the dimerization arm, α-helix, β-barrel, and Rossmann fold [7]. PRMT3 is essential for 80S ribosome maturation, which it performs by binding and methylating the 40S ribosomal protein S2 [8,9]. Accumulating evidence has highlighted the key roles of PRMT3 in tumorigenesis and multiple pathological processes [7]. Recent studies found that PRMT3 attenuates antiviral innate immunity triggered by DNA and RNA viruses by catalyzing asymmetric arginine dimethylation of the cytosolic DNA sensor cGAS and RNA sensor RIG-I [10,11].

Vesicle-associated membrane protein-associated protein A (VAPA) is an endoplasmic reticulum-integrated transmembrane protein featuring an N-terminal MSP domain, a central coiled-coil domain, and a C-terminal transmembrane domain [12]. VAPA exerts diverse physiological functions by interacting with two phenylalanines in an acidic tract (FFAT) motif-containing proteins via its N-terminal MSP domain or through homotypic interactions via its transmembrane domain, with the major sperm protein (MSP) domain being the major functional module [13,14]. VAPA participates in vesicular trafficking, membrane fusion, lipid metabolism, protein complex assembly, and cell motility [12]. Previous studies revealed that VAPA promotes viral replication through interactions with the hepatitis C virus proteins NS5A and NS5B [15,16], and facilitates norovirus replication through binding to an FFAT-like motif in the viral NS1/2 protein [17]. Meanwhile, IFITM3 competitively binds to VAPA to disrupt the VAPA–oxysterol-binding protein (OSBP) interaction, impairing endosomal cholesterol transport and restricting viral entry [18].

CRISPR/Cas9 screening has emerged as a powerful high-throughput tool for identifying host factors essential for viral infection, including influenza A virus [19,20], porcine deltacoronavirus (PDCoV) [21], and porcine pseudorabies virus (PRV) [22]. Genome-wide CRISPR/Cas9 knockout screens have identified host genes critical for multiple stages of the viral life cycle (e.g., adsorption, internalization, replication, assembly), such as *SLC35A1* [23], *PKCθ* [24], *IFITM3* [25], *YIPF5* [26], *RPSA* [27], and *ST3GAL4* [28] and elucidated their underlying mechanisms (e.g., regulating viral receptor expression, mediating apoptotic signaling, facilitating viral entry and replication factory formation, modulating host signaling and metabolism).

In this study, we performed genome-wide CRISPR/Cas9 knockout screening in Vero cells to select cell populations resistant to PEDV-induced CPEs. After four rounds of PEDV infection, surviving cells were enriched for several host genes, including proviral factors (*TMEM41B*, *VMP1*, *IFITM1*, *IFITM3*) and, notably, host restriction factors, among which PRMT3 exhibited a significant inhibitory effect on PEDV infection. Interestingly, we found that PRMT3 might modulate PEDV infection through pathways beyond innate immune suppression. Further mechanistic studies show that PRMT3 inhibits PEDV infection by regulating the PEDV-induced and VAPA-dependent antiviral autophagy process.

## Results

### Genome-scale CRISPR/Cas9 screening identified *PRMT3* as a restriction factor for PEDV infection

**Fig 1:**
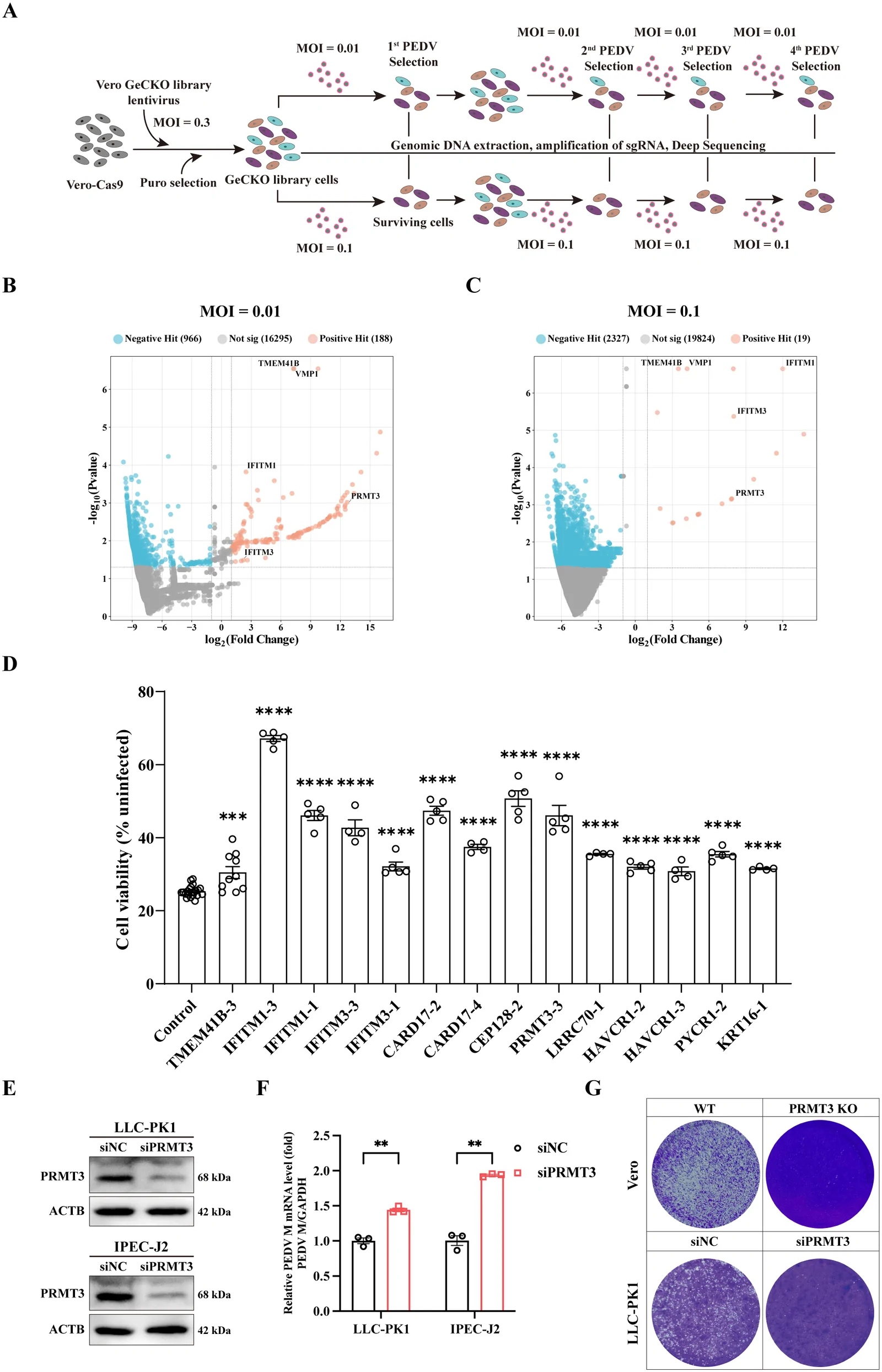
Genome-scale CRISPR/Cas9 screening identified *PRMT3* as a restriction factor for PEDV infection. (A) Schematic workflow of genome-wide CRISPR/Cas9 knockout screening in Vero cells. (B and C) Volcano plots showing gene enrichment in PEDV-surviving cells versus control cells at MOI = 0.01 (B) and MOI = 0.1 (C), with enrichment significance displayed as −log_10_(*P*-value). (D) Vero cells transduced with non-targeting sgRNA or individual gene-specific sgRNAs were infected with PEDV LW/L (MOI = 0.01). Cell viability was measured by CellTiter-Glo Luminescent Assay at 60 hpi (control sgRNA, *n* = 21; individual sgRNAs, *n* ≥ 4). (E and F) LLC-PK1 and IPEC-J2 cells were transfected with non-targeting siRNA (siNC) or *PRMT3*-specific siRNA, then infected with PEDV LW/L (MOI = 0.01) for 24 h. Endogenous PRMT3 protein levels were detected by Western blotting (E) to validate siRNA efficiency. PEDV M mRNA levels were quantified by RT-qPCR (F) to evaluate viral infection. (G) WT and PRMT3 KO Vero cells, as well as LLC-PK1 cells transfected with siNC or PRMT3-specific siRNA, were infected with PEDV LW/L for 60 h and stained with crystal violet. All data are representative of three independent experiments and are expressed as mean ± standard error of the mean (SEM). P-values were calculated using unpaired Student’s t-tests or one-way analysis of variance (ANOVA). Statistical significance is denoted as *P < 0.05, **P < 0.01, ***P < 0.001, and ****P < 0.0001. All Western blot results are representative of at least three independent experiments.

Genome-scale CRISPR/Cas9 knockout screening has revolutionized viral infection research by enabling systematic, high-throughput, unbiased identification of key nodes in virus–host interactions, shifting the field from single-gene studies to global functional profiling. However, nearly all current CRISPR library screening studies, which are based on virus-induced cytopathic effects (CPEs), have focused on identifying genes that affect viral loads while neglecting genes that directly influence virus-induced CPEs. In this study, we conducted a genome-wide CRISPR/Cas9 knockout screen in Vero cells based on PEDV-induced CPEs. The screening workflow is illustrated in Fig 1A. Briefly, Vero cells were infected with PEDV at multiplicities of infection (MOIs) of 0.01 and 0.1, and surviving cells were collected. Single-guide RNAs (sgRNAs) were amplified by polymerase chain reaction and subjected to next-generation sequencing. By calculating the log_2_ fold change and significance (−log_10_P-value) of sgRNA enrichment between control and surviving mutant cells, top-ranked candidate genes were identified. After four rounds of PEDV infection, multiple genes were enriched in surviving cells, including *TMEM41B*, *VMP1*, *IFITM3*, *IFITM1*, and *PRMT3* (Fig 1B and 1C).

Based on the screening results, we selected 10 candidate genes and generated single-knockout Vero cell lines for each gene. Knockout cells and control cells (without sgRNA) were simultaneously infected with the PEDV LW/L strain. Cell viability was assessed using the CellTiter-Glo luminescence assay at 60 hours post-infection (hpi). Knockout of *TMEM41B*, *IFITM1*, *IFITM3*, and *PRMT3* significantly alleviated PEDV-induced CPEs in Vero cells (Fig 1D). Contrarily, *PRMT3* knockout markedly enhanced PEDV infectivity in Vero cells.

To validate this phenotype, we designed and synthesized *PRMT3*-specific small interfering RNAs and transfected them into porcine kidney (LLC-PK1) and porcine small intestinal epithelial (IPEC-J2) cells (Fig 1E and S1A Fig). A viral infection assay confirmed that *PRMT3* silencing significantly promoted PEDV infection (Fig 1F). Crystal violet staining of PEDV-infected *PRMT3*-knockout (PRMT3 KO) Vero cells and *PRMT3*-silenced LLC-PK1 cells further demonstrated that *PRMT3* suppression reduced PEDV-induced CPEs and enhanced viral infectivity (Fig 1G). These data indicate that *PRMT3* suppression enhances PEDV infection and attenuates virus-induced cellular pathological damage.

### Changes in PRMT3 expression levels can significantly affect PEDV infection

**Fig 2:**
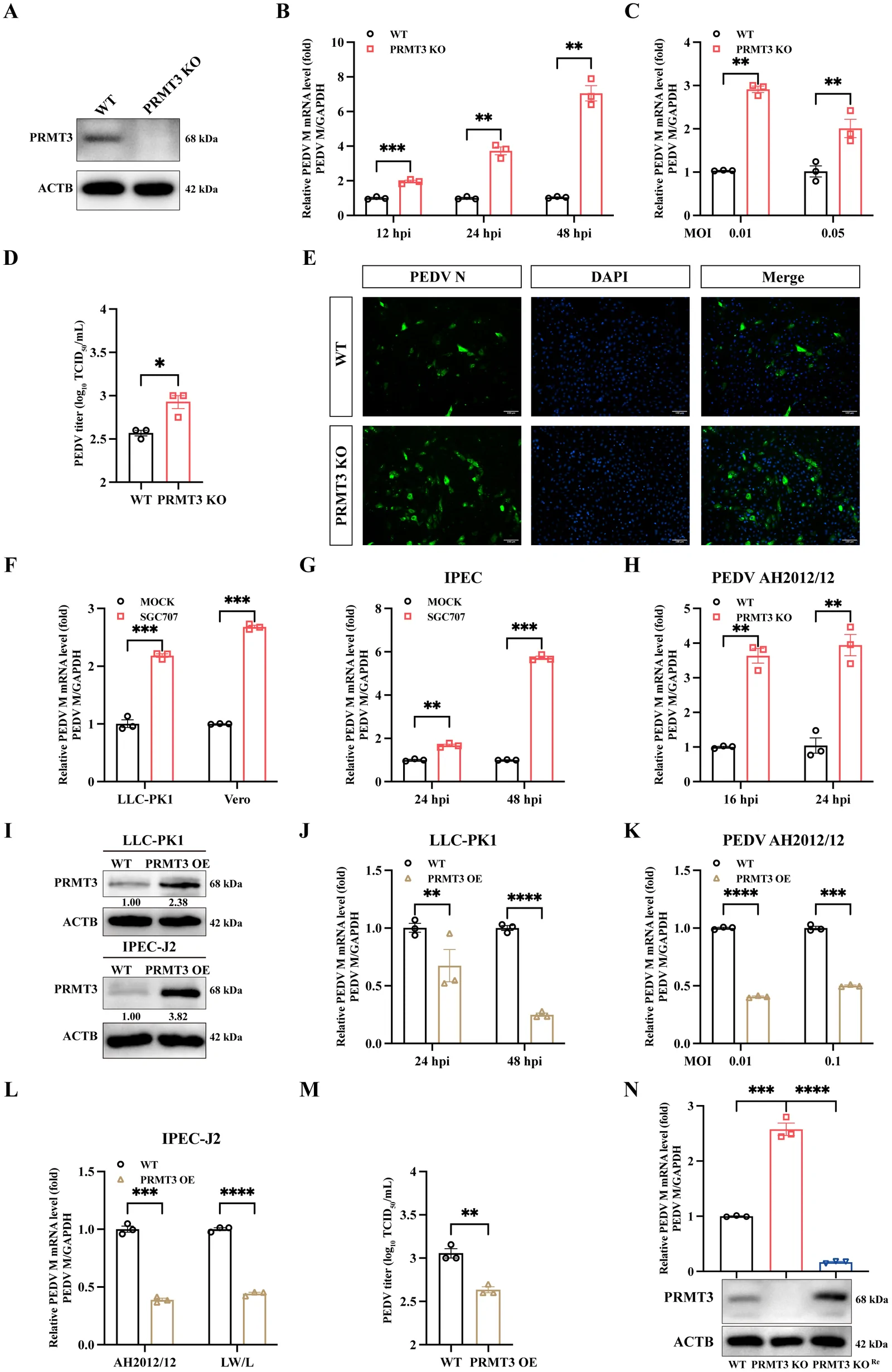
Changes in *PRMT3* expression levels can significantly affect PEDV infection. (A) Western blotting analysis of endogenous PRMT3 in WT and PRMT3 KO LLC-PK1 cells; β-actin was used as the internal control. (B and C) WT and PRMT3 KO cells were infected with PEDV LW/L (MOI = 0.01) for 12, 24, and 48 h (B), or with MOI = 0.01 and 0.05 for 24 h (C). Viral RNA was quantified by RT-qPCR. (D and E) WT and PRMT3 KO cells were infected with PEDV LW/L (MOI = 0.01) for 24 h. Viral titer was determined by TCID₅₀ assay (D), and PEDV N protein expression was detected by IFA (E). Scale bar, 100 µm. (F) LLC-PK1 and Vero cells were pre-treated with the PRMT3 inhibitor SGC707 for 24 h, then infected with PEDV LW/L (MOI = 0.01) for 24 h. Viral RNA was quantified by RT-qPCR. (G) Primary porcine intestinal epithelial cells were pre-treated with SGC707 for 24 h, then infected with PEDV LW/L for 24 and 48 h. Viral RNA was quantified by RT-qPCR. (H) WT and PRMT3 KO cells were infected with PEDV AH2012/12 (MOI = 0.01) for 16 and 24 h. Viral RNA was quantified by RT-qPCR. (I) Western blotting analysis of PRMT3 in WT, PRMT3 OE LLC-PK1, and PRMT3 OE IPEC-J2 cells; β-actin was used as the internal control. Band intensity was quantified by ImageJ. (J) WT and PRMT3 OE LLC-PK1 cells were infected with PEDV LW/L (MOI = 0.01) for 24 and 48 h. Viral RNA was quantified by RT-qPCR. (K) WT and PRMT3 OE LLC-PK1 cells were infected with PEDV AH2012/12 at MOI = 0.01 and 0.1 for 24 h. Viral RNA was quantified by RT-qPCR. (L) WT and PRMT3 OE IPEC-J2 cells were infected with PEDV LW/L or AH2012/12 (MOI = 0.1) for 24 h. Viral RNA was quantified by RT-qPCR. (M) WT and PRMT3 OE LLC-PK1 cells were infected with PEDV AH2012/12 (MOI = 0.1) for 24 h. Viral titer was determined by TCID₅₀ assay. (N) WT, PRMT3 KO, and PRMT3 KO-Rescue (PRMT3 KO-Re) cells were infected with PEDV LW/L (MOI = 0.01) for 24 h. Viral RNA was quantified by RT-qPCR, and PRMT3 expression was detected by Western blotting. All data are representative of three independent experiments and are expressed as mean ± standard error of the mean (SEM). P-values were calculated using unpaired Student’s t-tests or one-way analysis of variance (ANOVA). Statistical significance is denoted as *P < 0.05, **P < 0.01, ***P < 0.001, and ****P < 0.0001. All Western blot results are representative of at least three independent experiments.

To further define the role of PRMT3 in PEDV infection, we generated a PRMT3 KO LLC-PK1 cell line using CRISPR/Cas9 (Fig 2A). Wild-type (WT) and PRMT3 KO cells were infected with PEDV at different MOIs and time points. Reverse transcription-quantitative polymerase chain reaction (RT-qPCR), 50% tissue culture infectious dose assay (TCID₅₀), and immunofluorescence assay (IFA) results consistently demonstrated that *PRMT3* knockout significantly enhanced PEDV infectivity in LLC-PK1 cells (Fig 2D and 2E).

To further validate these findings, we isolated and cultured primary porcine intestinal epithelial cells (IPEC-WW; S1B Fig). Epithelial marker genes including *Villin* and other epithelium-specific genes were readily detectable in IPEC-WW cells (S1C and S1D Fig). Prior to PEDV infection, Vero, LLC-PK1, and IPEC-WW cells were pretreated with the selective PRMT3 inhibitor SGC707 for 24 h. RT-qPCR revealed that PRMT3 inhibition enhanced PEDV infection in all three cell lines (Fig 2F and 2G). To confirm the universality of this effect, we tested the PEDV AH2012/12 strain, finding that *PRMT3* knockout also significantly promoted infection by this strain (Fig 2H).

We further established stable *PRMT3*-overexpressing (PRMT3 OE) IPEC-J2 and LLC-PK1 cells using the PiggyBac transposon system (Fig 2I and S1E Fig). RT-qPCR, TCID₅₀, and IFA demonstrated that *PRMT3* overexpression significantly suppressed infection by both the LW/L and AH2012/12 strains (Fig 2J–2M, S1F, and S1G Fig). In addition, we generated an enzymatically inactive mutant of PRMT3 (PRMT3-3A, G263A/C264A/G265A) [9] and overexpressed it in LLC-PK1 cells. Subsequent PEDV infection assays demonstrated that the inhibitory effect of PRMT3 against PEDV infection is independent of its methyltransferase activity (S1H Fig). Finally, rescue experiments in PRMT3 KO cells illustrated that restoring PRMT3 expression reversed the proviral phenotype (Fig 2N). Collectively, these results demonstrate that PRMT3 expression significantly suppresses PEDV infection.

### PEDV infection might upregulate PRMT3 *via* the transcription factor SP1

**Fig 3:**
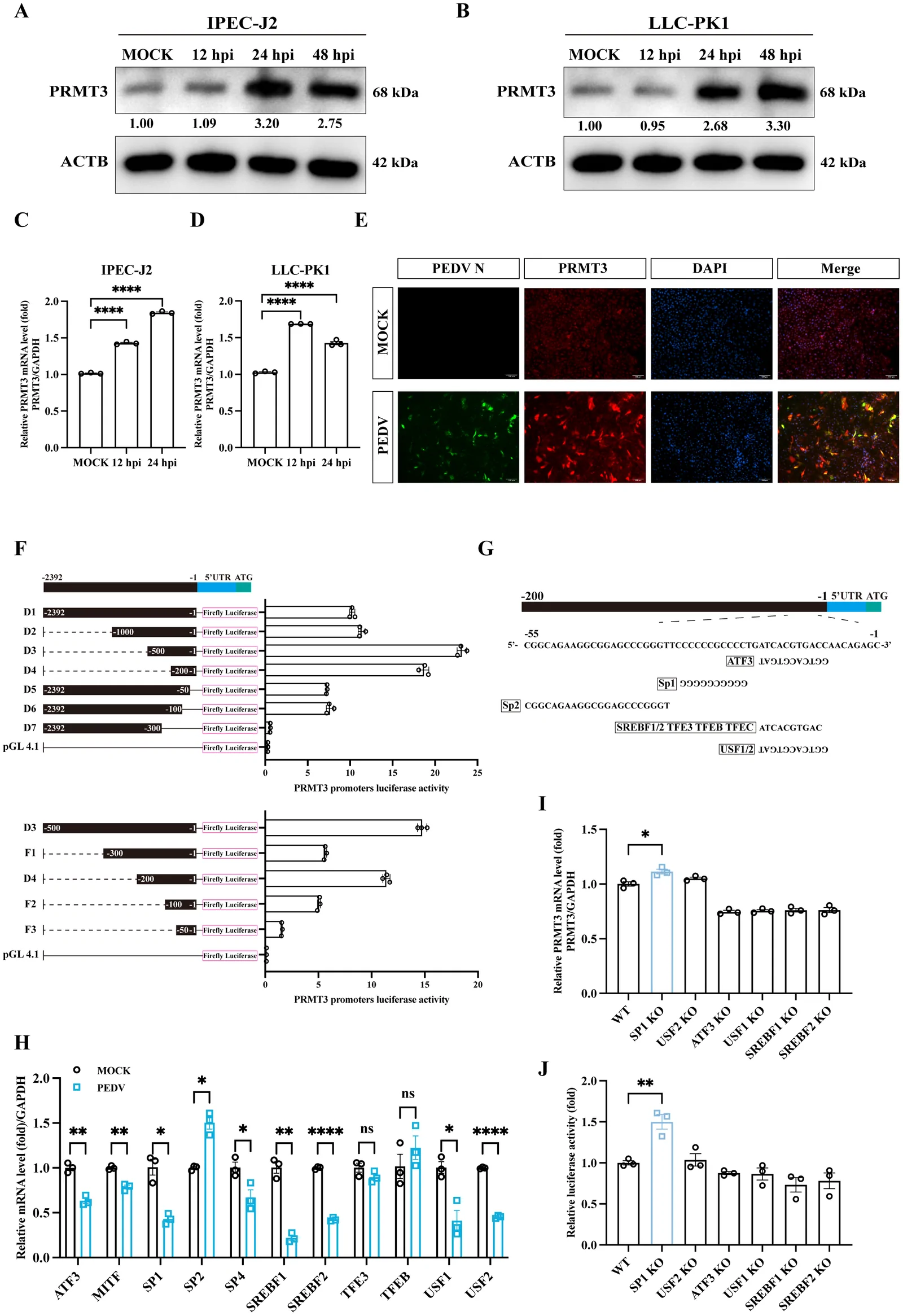
PEDV infection might upregulate PRMT3 *via* the transcription factor SP1. (A and B) Western blotting analysis of endogenous PRMT3 in mock-infected and PEDV LW/L-infected IPEC-J2 and LLC-PK1 cells at 24 hpi. (C–E) PRMT3 expression in mock-infected and PEDV LW/L-infected IPEC-J2 and LLC-PK1 cells was detected by RT-qPCR (C and D) and IFA (E) at 24 hpi. (F) HEK293T cells were co-transfected with pGL4.1 luciferase vectors carrying full-length or truncated PRMT3 promoters (D1–D7, F1–F3) and the pRL-TK internal control vector. Luciferase activity was measured by dual-luciferase assay. (G) Transcription factor binding sites in the PRMT3 core promoter were predicted using the JASPAR database. (H) IPEC-J2 cells were infected with PEDV LW/L (MOI = 0.1) for 24 h. The mRNA levels of candidate transcription factors were quantified by RT-qPCR. (I) PRMT3 mRNA levels in WT and transcription factor KO IPEC-J2 cells were detected by RT-qPCR. (J) WT and transcription factor KO IPEC-J2 cells were co-transfected with pGL4.1-D4 (PRMT3 core promoter luciferase vector) and pRL-TK. Luciferase activity was measured by dual-luciferase assay. All data are representative of three independent experiments and are expressed as mean ± standard error of the mean (SEM). P-values were calculated using unpaired Student’s t-tests or one-way analysis of variance (ANOVA). Statistical significance is denoted as *P < 0.05, **P < 0.01, and ****P < 0.0001. All Western blot results are representative of at least three independent experiments.

To explore the regulatory relationship between PEDV infection and PRMT3 expression, we infected IPEC-J2 and LLC-PK1 cells with PEDV at MOIs of 0.1 and 0.01, respectively. RT-qPCR, western blotting (WB), and IFA demonstrated that PRMT3 expression was significantly higher in PEDV-infected cells than uninfected controls (Fig 3A–3E).

To dissect the molecular mechanism underlying PRMT3 upregulation, we cloned the full-length (2392 bp) and truncated PRMT3 promoters (D1–D7) into the pGL4.1 luciferase vector and measured their transcriptional activity in HEK293T cells. The promoter fragment spanning nucleotides −500 to −1 exhibited the strongest transcriptional activity (Fig 3F). Further truncation of this region (F1–F3) localized the core PRMT3 promoter to the −200 to −1 region.

Bioinformatic analysis predicted potential transcription factor binding sites within the core promoter (Fig 3G). We then screened for transcription factors with altered expression in PEDV-infected cells, uncovering *SP1*, *ATF3*, *SREBF1*, *SREBF2*, *USF1*, and *USF2* as candidates (Fig 3H). Subsequent knockout experiments in IPEC-J2 cells revealed that *SP1* deficiency significantly enhanced *PRMT3* transcription and *PRMT3* promoter-driven luciferase activity (Fig 3I and 3J). These findings demonstrate that PEDV infection might upregulate PRMT3 by downregulating SP1.

### PRMT3 modulates PEDV infection through pathways beyond innate immune suppression

**Fig 4:**
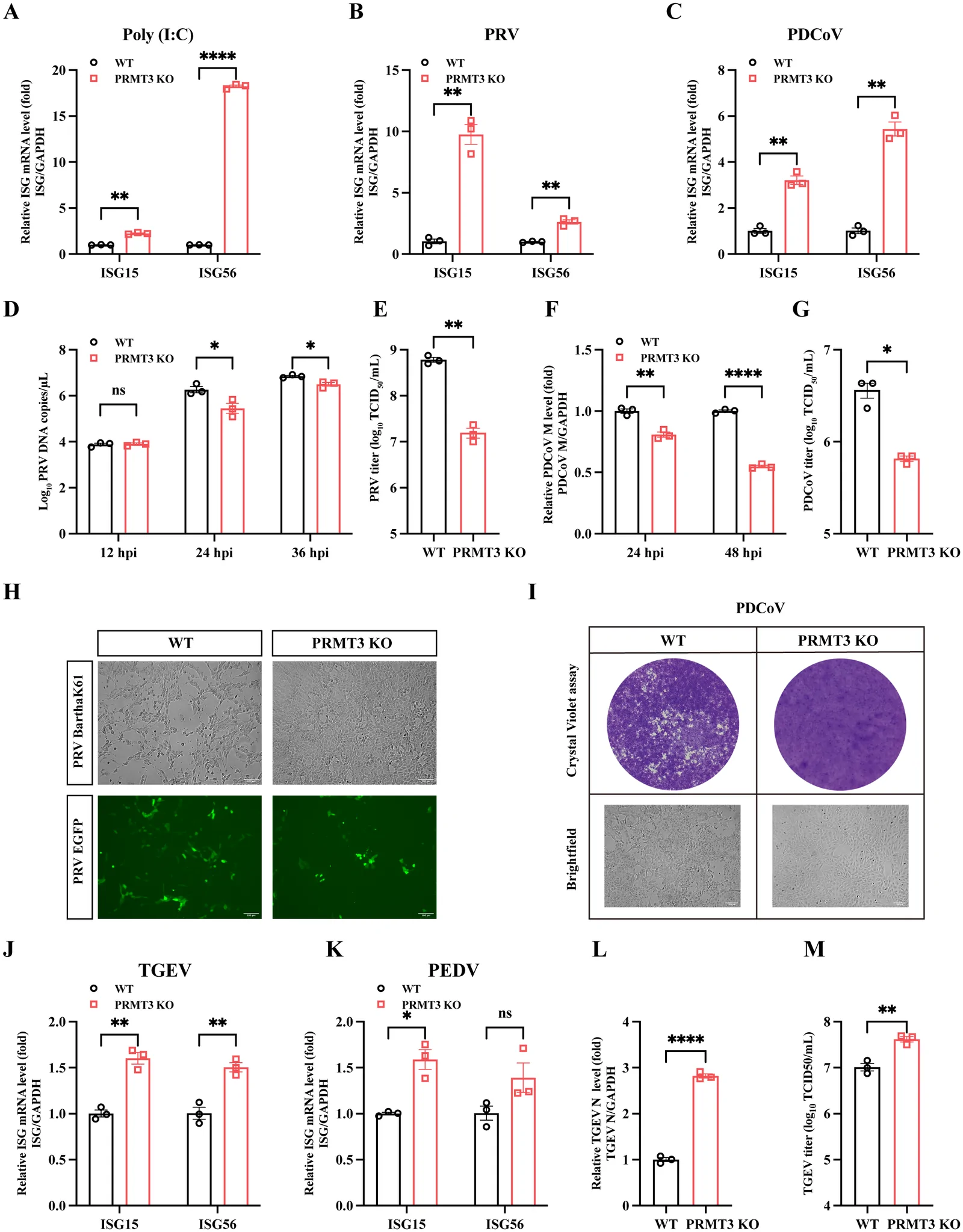
PRMT3 modulate PEDV infection through pathways beyond innate immune suppression. (A) WT and PRMT3 KO LLC-PK1 cells were treated with poly(I:C) for 24 h. mRNA levels of ISG15 and ISG56 were quantified by RT-qPCR. (B and C) WT and PRMT3 KO LLC-PK1 cells were infected with PRV-BarthaK61 (MOI = 0.001) (B) or PDCoV-CZ2020 (MOI = 0.1) (C) for 24 h. mRNA levels of ISG15 and ISG56 were quantified by RT-qPCR. (D and E) WT and PRMT3 KO LLC-PK1 cells were infected with PRV-BarthaK61 (MOI = 0.001) for 24 h. PRV genomic copy number was measured by absolute RT-qPCR (D), and viral titer was determined by TCID₅₀ assay (E). (F and G) WT and PRMT3 KO cells were infected with PDCoV-CZ2020 (MOI = 0.1) for 24 and 48 h. Viral RNA was quantified by RT-qPCR (F), and viral titer was determined by TCID₅₀ assay (G). (H) WT and PRMT3 KO LLC-PK1 cells were infected with PRV-BarthaK61 or PRV-EGFP (MOI = 0.001) for 24 h. CPE and GFP fluorescence were observed. (I) WT and PRMT3 KO LLC-PK1 cells were infected with PDCoV-CZ2020 (MOI = 0.1) for 24 h, then fixed and stained with crystal violet to visualize CPE. (J and K) WT and PRMT3 KO cells were infected with PEDV LW/L (J) or TGEV-SD/L (K) for 24 h. mRNA levels of ISG15 and ISG56 were quantified by RT-qPCR. (L and M) WT and PRMT3 KO LLC-PK1 cells were infected with TGEV-SD/L (MOI = 0.01) for 24 h. Viral RNA was quantified by RT-qPCR (L), and viral titer was determined by TCID₅₀ assay (M). All data are representative of three independent experiments and are expressed as mean ± standard error of the mean (SEM). P-values were calculated using unpaired Student’s t-tests or one-way analysis of variance (ANOVA). Statistical significance is denoted as ns: not significant (P > 0.05), *P < 0.05, **P < 0.01, and ****P < 0.0001. All Western blot results are representative of at least three independent experiments.

Previous studies reported that PRMT3 negatively regulates antiviral innate immunity triggered by RNA and DNA viruses (*e.g.*, vesicular stomatitis virus) by asymmetrically methylating intracellular nucleic acid sensors [11,29]. Consistent with these reports, PRMT3 KO LLC-PK1 cells displayed enhanced expression of the interferon-stimulated genes *ISG15* and *ISG56* following poly(I:C) stimulation (Fig 4A).

To determine whether PRMT3 exerts virus-specific effects, we infected PRMT3 KO LLC-PK1 and WT cells with PRV, PDCoV, transmissible gastroenteritis virus (TGEV), and PEDV. RT-qPCR illustrated that PRMT3 KO cells mounted stronger antiviral innate immune responses against PRV and PDCoV (Fig 4B and 4C), and *PRMT3* knockout significantly inhibited PRV and PDCoV infection (Fig 4D–4I). Contrarily, although PRMT3 KO cells also exhibited enhanced innate immune responses to TGEV and PEDV (Fig 4J and 4K), *PRMT3* knockout significantly promoted TGEV infection (Fig 4L and 4M)—mirroring its effect on PEDV. These data suggest that the known innate immune regulatory function of PRMT3 is insufficient to fully explain its inhibitory effect against PEDV, suggesting that PRMT3 can modulate PEDV infection through pathways beyond innate immune suppression.

### PRMT3 might restrict PEDV infection *via* an interaction with VAPA

**Fig 5:**
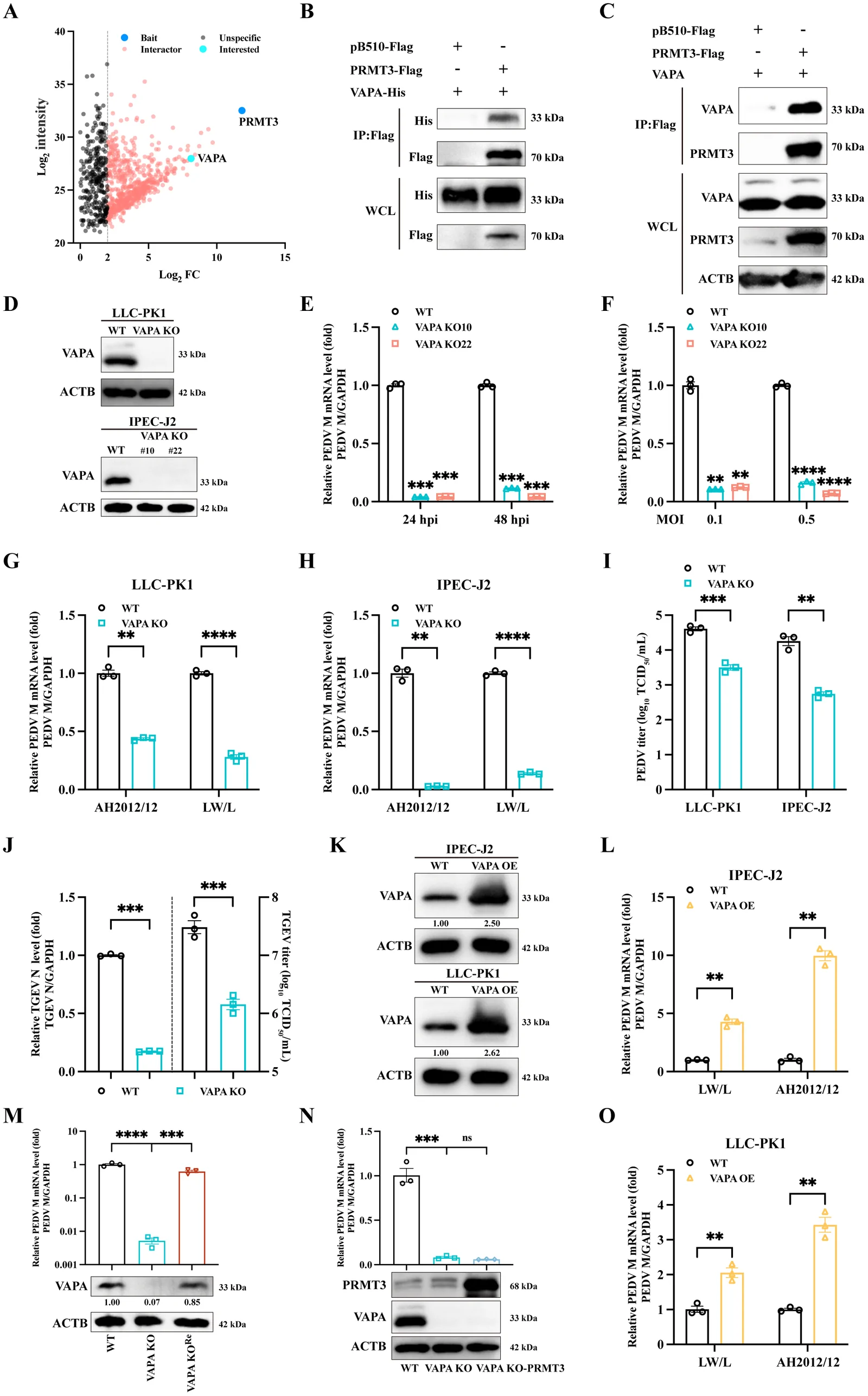
PRMT3 might restrict PEDV infection *via* an interaction with VAPA. (A) Volcano plot of PRMT3-His IP-MS analysis in PRMT3 KO LLC-PK1 cells. Proteins with >4-fold change were defined as candidate PRMT3-interacting proteins. LFQ intensity was used for relative protein quantification. (B) HEK293T cells were co-transfected with PRMT3-Flag and VAPA-His plasmids. The interaction between PRMT3 and VAPA was detected by Co-IP. (C) IPEC-J2 cells were transfected with PRMT3-Flag plasmid. Endogenous VAPA was immunoprecipitated to validate its interaction with PRMT3. (D) Western blotting analysis of endogenous VAPA in WT, VAPA KO IPEC-J2, and VAPA KO LLC-PK1 cells; β-actin was used as the internal control. (E and F) WT and VAPA KO IPEC-J2 cells were infected with PEDV AH2012/12 (MOI = 0.1) for 24 and 48 h (E), or with MOI = 0.1 and 0.5 for 24 h (F). Viral RNA was quantified by RT-qPCR. (G and H) WT and VAPA KO LLC-PK1/IPEC-J2 cells were infected with PEDV LW/L or AH2012/12 (MOI = 0.1) for 24 h. Viral RNA was quantified by RT-qPCR. (I) WT and VAPA KO LLC-PK1/IPEC-J2 cells were infected with PEDV AH2012/12 (MOI = 0.1) for 24 h. Viral titer was determined by TCID₅₀ assay. (J) WT and VAPA KO IPEC-J2 cells were infected with TGEV-SD/L (MOI = 0.01) for 24 h. Viral RNA and titer were quantified by RT-qPCR and TCID₅₀ assay, respectively. (K) Western blotting analysis of VAPA in WT, VAPA OE LLC-PK1, and VAPA OE IPEC-J2 cells; β-actin was used as the internal control. Band intensity was quantified by ImageJ. (L and O) WT and VAPA OE IPEC-J2 (L) / LLC-PK1 (O) cells were infected with PEDV LW/L or AH2012/12 (MOI = 0.1) for 24 h. Viral RNA was quantified by RT-qPCR. (M) WT, VAPA KO, and VAPA KO-Rescue (VAPA KO-Re) cells were infected with PEDV LW/L (MOI = 0.1) for 24 h. Viral RNA was quantified by RT-qPCR, and VAPA expression was detected by Western blotting. (N) WT, VAPA KO, and VAPA KO cells overexpressing PRMT3 were infected with PEDV AH2012/12 (MOI = 0.1) for 24 h. Viral RNA was quantified by RT-qPCR. VAPA and PRMT3 expression was detected by Western blotting. All data are representative of three independent experiments and are expressed as mean ± standard error of the mean (SEM). P-values were calculated using unpaired Student’s t-tests or one-way analysis of variance (ANOVA). Statistical significance is denoted as ns: not significant (P > 0.05), **P < 0.01, ***P < 0.001, and ****P < 0.0001. All Western blot results are representative of at least three independent experiments.

To identify the molecular mechanism by which PRMT3 regulates PEDV infection, we expressed His-tagged PRMT3 in PRMT3 KO LLC-PK1 cells and performed immunoprecipitation coupled with mass spectrometry (IP-MS) under PEDV-infected conditions to screen for PRMT3-interacting proteins (S1I–S1K Fig). Relative protein quantification based on label-free quantification (LFQ) intensity and volcano plot analysis identified VAPA as a candidate PRMT3 binding partner (Fig 5A). Gene Ontology and Kyoto Encyclopedia of Genes and Genomes enrichment analyses further supported a functional link between PRMT3 and VAPA (S1L and S2A Fig). Co-immunoprecipitation (Co-IP) assays in HEK293T and IPEC-J2 cells confirmed the interaction between PRMT3 and VAPA (Fig 5B, 5C, and S2B Fig), and immunofluorescence colocalization assays validated their intracellular co-localization (S2C Fig).

To assess the role of VAPA in PEDV infection, we generated *VAPA*-knockout (VAPA KO) IPEC-J2 and LLC-PK1 cell lines (Fig 5D). Infection assays with PEDV (LW/L and AH2012/12 strains) illustrated that *VAPA* knockout significantly suppressed PEDV infection in both cell lines (Fig 5E–5I). Virus-specificity testing revealed that *VAPA* knockout additionally inhibited TGEV infection, but it had no effect on PDCoV or PRV infection (Fig 5J, S2D and S2E Fig). We further established *VAPA*-overexpressing (VAPA OE) IPEC-J2 and LLC-PK1 cell lines (Fig 5K) and found that *VAPA* overexpression significantly enhanced PEDV infection (Fig 5L and 5O). Rescue experiments in VAPA KO cells confirmed that VAPA is essential for efficient PEDV infection (Fig 5M). Taken together, these results demonstrate that VAPA plays a critical role in PEDV infection.

Further experiments revealed that overexpression of PRMT3 in VAPA KO cells failed to suppress PEDV infection (Fig 5N). However, neither PRMT3 knockout nor its overexpression altered VAPA expression (Fig S2F). Collectively, these data indicate that PRMT3 may inhibit PEDV infection through interaction with VAPA, while the precise mechanism underlying this interaction remains to be elucidated.

### VAPA regulates PEDV infection at the entry and replication stages

**Fig 6:**
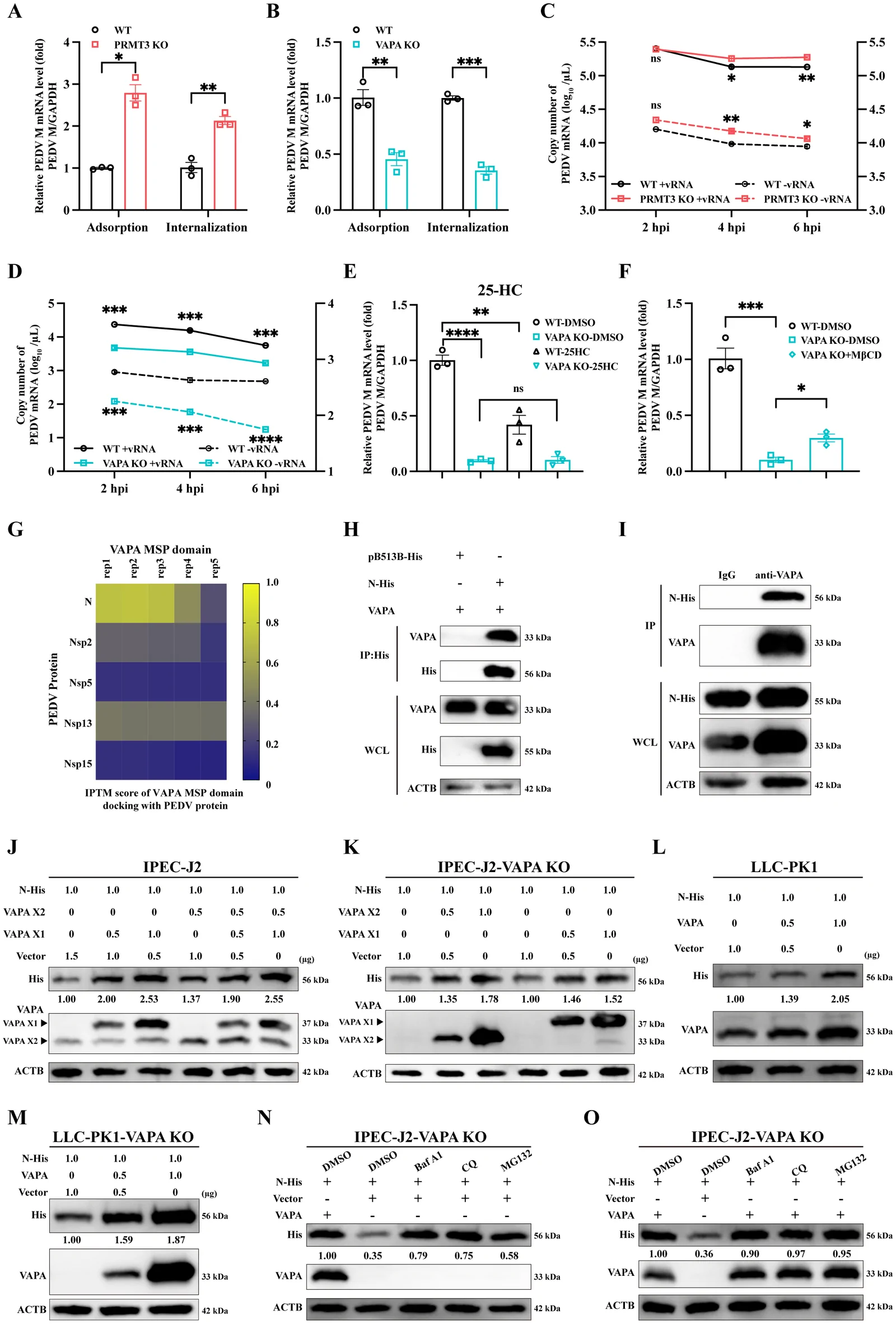
VAPA regulates PEDV infection at the entry and replication stages. (A) Viral adsorption and internalization were evaluated in WT and PRMT3‑KO cells. Cells were inoculated with PEDV LW/L (MOI = 1) at 4 °C for 1 h to assess viral adsorption. For viral internalization, infected cells were washed with PBS, incubated at 37°C for 1 h, and then subjected to acid washing to remove non-internalized surface virions. Viral RNA was extracted and quantified by RT-qPCR. (B) Viral adsorption and internalization of PEDV LW/L (MOI = 1) in WT and VAPA‑KO IPEC‑J2 cells were determined using the same protocol as in (A). (C) WT and PRMT3 KO cells were infected with PEDV LW/L (MOI = 1). Positive-sense (+vRNA) and negative-sense (−vRNA) viral RNA were quantified by RT-qPCR. (D) Viral RNA replication in WT and VAPA KO cells was measured as in (C). (E) WT and VAPA KO cells were infected with PEDV AH2012/12 (MOI = 0.1) for 2 h, then treated with 25-HC or DMSO for 22 h. Viral RNA was quantified by RT-qPCR. (F) WT, VAPA KO, and MβCD-treated VAPA KO cells were infected with PEDV LW/L (MOI = 1) at 4°C for 1 h, then incubated at 37°C for 1 h. Viral entry was quantified by RT-qPCR at 4 hpi. (G) Heatmap of interface prediction template modeling scores for the VAPA MSP domain docked with PEDV proteins using AlphaFold3. (H) HEK293T cells were co-transfected with VAPA and N-His plasmids. The interaction between VAPA and PEDV N protein was detected by Co-IP. (I) IPEC-J2 cells were transfected with N-His plasmid. Endogenous VAPA was immunoprecipitated to validate its interaction with N protein. (J and K) IPEC-J2 and VAPA KO IPEC-J2 cells were co-transfected with N-His and increasing doses of VAPA-X1/VAPA-X2 plasmids. N protein levels were detected by Western blotting and quantified. (L and M) LLC-PK1 and VAPA KO LLC-PK1 cells were co-transfected with N-His and increasing doses of VAPA-X2 plasmid. N protein levels were detected by Western blotting and quantified. (N and O) VAPA KO IPEC-J2 cells were transfected with N-His ± VAPA plasmid, then treated with DMSO, MG132, CQ, or Baf A1 for 9 h. N protein levels were detected by Western blotting and quantified. All data are representative of three independent experiments and are expressed as mean ± standard error of the mean (SEM). P-values were calculated using unpaired Student’s t-tests or one-way analysis of variance (ANOVA). Statistical significance is denoted as ns: not significant (P > 0.05), *P < 0.05, **P < 0.01, ***P < 0.001, and ****P < 0.0001. All Western blot results are representative of at least three independent experiments.

We next investigated the specific stages of PEDV infection regulated by PRMT3 and VAPA. *PRMT3* knockout promoted PEDV adsorption and internalization (Fig 6A), whereas *VAPA* knockout inhibited these entry steps (Fig 6B). Similarly, PRMT3 knockout enhanced PEDV genome replication (Fig 6C), whereas VAPA knockout suppressed replication (Fig 6D).

Previous studies reported that VAPA regulates intracellular cholesterol homeostasis by interacting with OSBP, thereby modulating the entry of vesicular stomatitis virus and influenza A virus [18,30]. To determine whether this mechanism applies to PEDV, we treated cells with 25-Hydroxycholesterol (25-HC)— a VAPA–OSBP interaction inhibitor [31,32] —to PEDV infection. 25-HC treatment significantly suppressed PEDV infection by blocking the VAPA–OSBP interaction (S2G Fig and Fig 6E). To confirm the role of cholesterol transport, we treated VAPA KO cells with methyl-β-cyclodextrin (MβCD), a cholesterol-depleting reagent [31] which restored PEDV entry by reversing late endosomal cholesterol accumulation (S2H Fig and Fig 6F). These results demonstrate that VAPA promotes PEDV entry by facilitating OSBP-dependent cholesterol transport in late endosomes.

Previous research found that VAPA facilitates norovirus replication by binding to the FFAT-like motif in the viral NS1/2 protein [17]. To identify the viral factor mediating the effect of VAPA on replication, we screened PEDV proteins for FFAT-like motifs, identifying five candidates (Nsp2, Nsp5, Nsp13, Nsp15, and N) [13,33,34]. Molecular docking of the VAPA MSP domain with these viral proteins predicted the N protein as the primary binding partner (Fig 6G). Co-IP assays in HEK293T and IPEC-J2 cells confirmed the direct interaction between VAPA and the PEDV N protein (Fig 6H and 6I), whereas no interaction with Nsp5 was observed (S2I Fig).

To characterize the functional contribution of the VAPA–N protein interaction during PEDV infection, we generated expression constructs encoding two VAPA splice variants (VAPA X1 and VAPA X2) and evaluated their functional interplay with the N protein in cells ectopically expressing the viral N protein. In both WT and VAPA‑KO IPEC‑J2 cells, overexpression of either VAPA X1 or VAPA X2 attenuated N protein degradation in a concentration‑dependent manner (Fig 6J and 6K). Consistent results were also observed in LLC‑PK1 and VAPA‑KO LLC‑PK1 cells, further validating that VAPA restrains N protein degradation (Fig 6L and 6M). To dissect the mechanism by which VAPA stabilizes the N protein, we ectopically expressed PEDV N protein in VAPA KO cells, which were subsequently treated with the proteasome inhibitor MG132 and the autophagy inhibitors chloroquine (CQ) and bafilomycin A1 (Baf A1). Autophagy inhibitors exerted a much stronger inhibitory effect on N protein degradation than MG132 (Fig 6N and 6O). Collectively, these results demonstrate that VAPA promotes PEDV infection at both the entry and replication stages, specifically facilitating viral entry by interacting with OSBP and enhancing viral replication by binding to the N protein.

### PRMT3 promotes antiviral autophagy by disrupting the VAPA–N protein interaction

**Fig 7:**
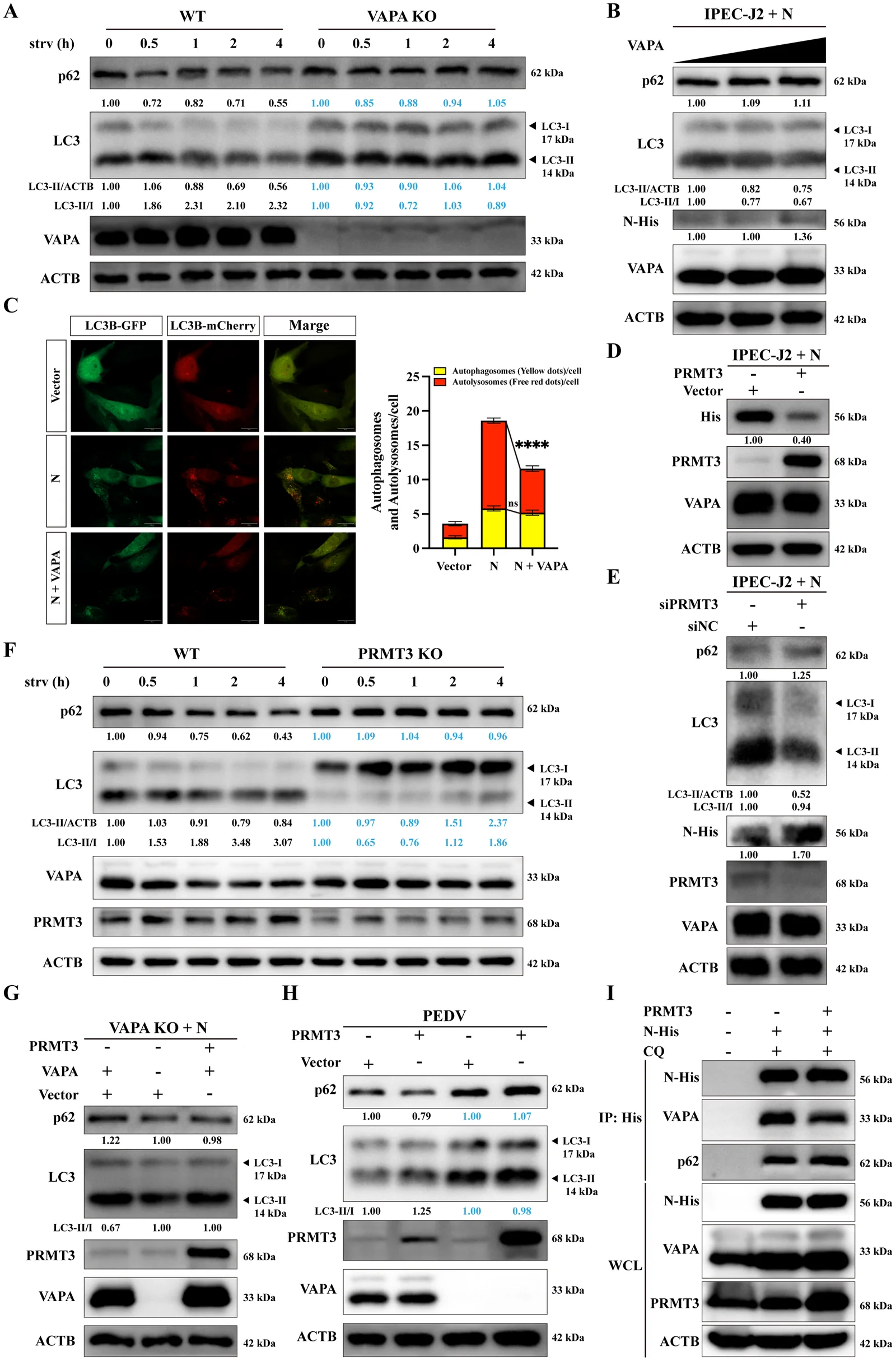
PRMT3 promotes antiviral autophagy by disrupting the VAPA–N protein interaction. (A) WT and VAPA KO IPEC-J2 cells were starved for the indicated times. LC3 and p62 levels were detected by Western blotting. The LC3-II/I ratio and protein levels were quantified and normalized to 0 h. (B) IPEC-J2 cells were co-transfected with N-His and increasing doses of VAPA plasmid. LC3, p62, and N protein levels were detected by Western blotting and quantified. (C) IPEC-J2 cells were infected with Ad-mCherry-GFP-LC3B, then transfected with vector, N-His, or N-His + VAPA plasmids. Autophagic flux was observed by fluorescence microscopy. Scale bar, 20 µm. The numbers of intracellular red and yellow puncta were quantified to assess autophagic flux. (D) IPEC-J2 cells were co-transfected with N-His and PRMT3 plasmids. N protein levels were detected by Western blotting and quantified. (E) IPEC-J2 cells were co-transfected with N-His and siNC or PRMT3-specific siRNA. LC3, p62, and N protein levels were detected by Western blotting and quantified. (F) WT and PRMT3 KO LLC-PK1 cells were starved for the indicated times. LC3 and p62 levels were detected by Western blotting, quantified, and normalized to 0 h. (G) VAPA KO IPEC-J2 cells were transfected with N-His ± PRMT3 and/or VAPA plasmids. LC3 and p62 levels were detected by Western blotting and quantified. (H) WT and VAPA KO IPEC-J2 cells were transfected with PRMT3 or vector, then infected with PEDV LW/L (MOI = 0.1) for 24 h. LC3 and p62 levels were detected by Western blotting, quantified, and normalized to vector-transfected controls. (I) IPEC-J2 cells were co-transfected with N-His and PRMT3 expression plasmids, followed by treatment with CQ for 9 h. Co-IP assays were performed to examine the effects of PRMT3 on the interactions of N-His with endogenous VAPA and p62. Autophagosome dots in each cell were counted by selecting at least three random fields of view per sample to assess the level of autophagic flux, and the results were presented as mean ± standard error of the mean (SEM). P-values were calculated using unpaired Student’s t-tests. Statistical significance is denoted as ns: not significant (P > 0.05) and ****P < 0.0001. The Western blot data are representative of at least three independent experiments.

Previous research implicated VAPA in autophagosome biogenesis [35]. The results revealed that VAPA depletion impaired starvation-induced autophagosome formation in IPEC-J2 cells, as reflected by decreased LC3-II/LC3-I ratios and attenuated p62 degradation (Fig 7A). To delineate the regulatory effects of VAPA and PRMT3 on the PEDV N protein, we ectopically expressed the PEDV N protein in cells to examine how PRMT3 and VAPA modulate the autophagic degradation of the viral N protein. Subsequent assays illustrated that VAPA inhibited PEDV N protein-induced autophagy in a concentration-dependent manner (Fig 7B), and autophagic flux analysis using Ad-mCherry-GFP-LC3B further verified that VAPA suppressed N protein-triggered autophagy (Fig 7C). Concurrently, our data indicated that during VAPA-mediated inhibition of autophagic N protein degradation, VAPA disrupted the interaction between p62 and the PEDV N protein while exerting no effect on mTOR signaling (S3A and S3B Fig). Collectively, these findings demonstrate that VAPA is essential for starvation-induced autophagy, but it inhibits the selective autophagic degradation of the PEDV N protein.

We next investigated the role of PRMT3 in this regulatory process. *PRMT3* overexpression accelerated N protein degradation (Fig 7D), whereas *PRMT3* knockdown impeded selective autophagic N protein degradation (Fig 7E). Furthermore, inhibition of autophagosome degradation by CQ abolished the ability of PRMT3 to promote autophagic PEDV N protein degradation. Meanwhile, the enzymatically inactive PRMT3 mutant retained the ability to promote autophagic N protein degradation, confirming that the ability of PRMT3 to facilitate autophagic degradation of the PEDV N protein is independent of its methyltransferase activity (S3C Fig). Consistently, autophagic flux assays using the Ad-mCherry-GFP-LC3B further validated that PRMT3 potentiated the selective autophagic degradation of the PEDV N protein (S3D Fig). PRMT3 was also indispensable for efficient autophagy induction under starvation conditions (Fig 7F). Whereas PEDV infection induces autophagy in host cells [36,37], *PRMT3* knockout attenuated antiviral autophagy in LLC-PK1 cells (S3E Fig). Consistently, autophagic flux assays using the Ad-mCherry-GFP-LC3B further validated that PRMT3 potentiated PEDV-triggered antiviral autophagy (S3F Fig). These findings demonstrate that PRMT3 promotes the selective autophagic degradation of the PEDV N protein and serves as a key regulator of both starvation-induced autophagy and antiviral autophagy.

To further dissect the functional crosstalk among PRMT3, VAPA, and autophagy, we evaluated the regulatory role of PRMT3 in autophagy in VAPA KO IPEC-J2 cells. PRMT3 antagonized the inhibitory effect of VAPA on N protein-selective autophagy (Fig 7G). Furthermore, PRMT3 facilitated antiviral autophagy in WT cells but not in VAPA KO cells, confirming that the regulatory function of PRMT3 on autophagy is VAPA-dependent (Fig 7H). Mechanistically, Co-IP assays in IPEC‑J2 cells revealed that PRMT3 attenuated the interaction between the PEDV N protein and VAPA, but enhanced the association of the N protein with the autophagy adaptor p62 (Fig 7I), indicating that PRMT3 mediates the selective autophagic degradation of the N protein by disrupting the VAPA–N protein interaction. Collectively, these findings demonstrate that VAPA inhibits autophagic PEDV N protein degradation, whereas PRMT3 antagonizes VAPA to enhance antiviral autophagy and ultimately restrict PEDV infection; the exact molecular mechanism underlying PRMT3-mediated VAPA regulation remains to be fully characterized.

### Critical domains and residues governing the VAPA–N protein interaction

**Fig 8:**
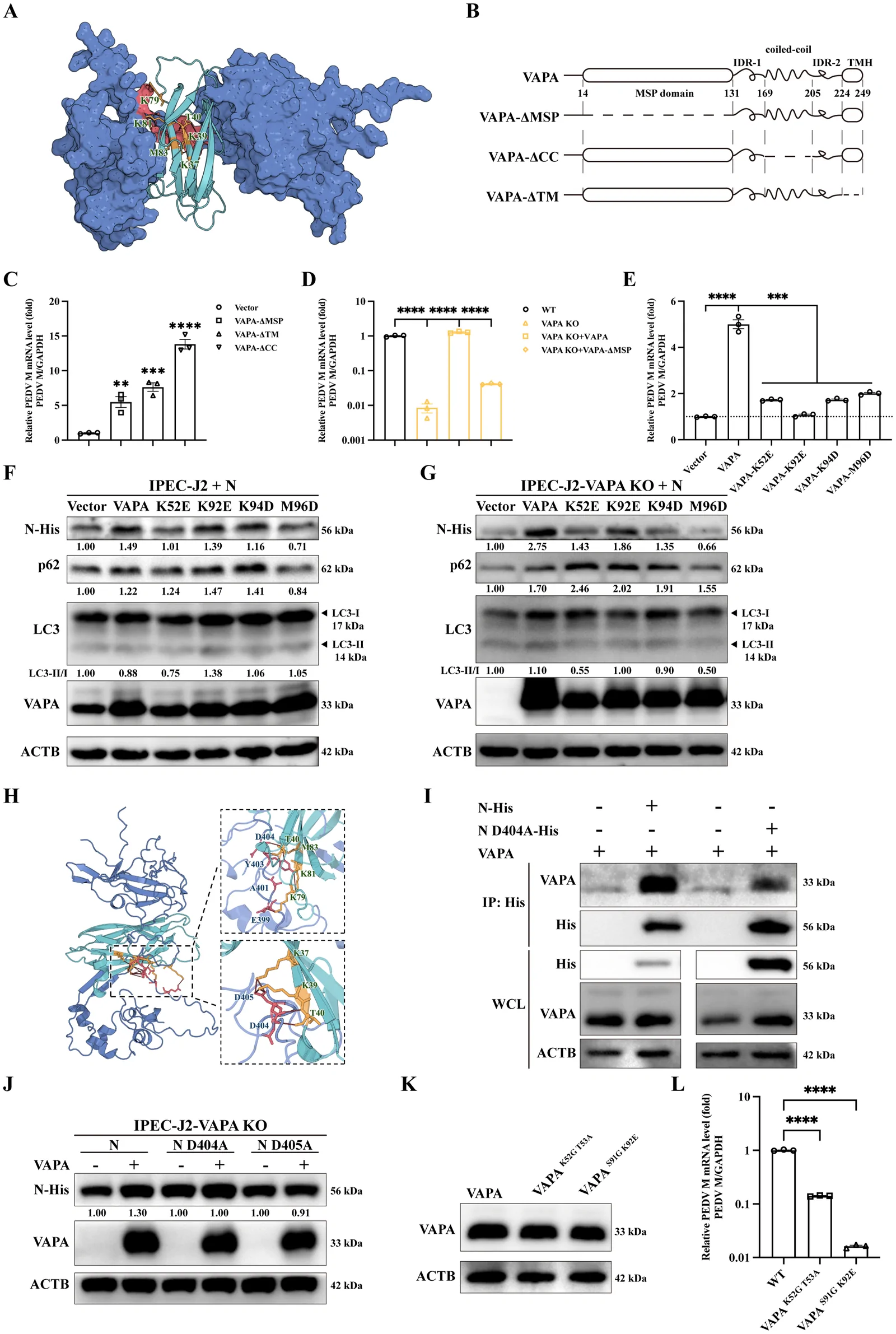
Critical domains and residues governing the VAPA–N protein interaction. (A) Molecular docking analysis of the interaction between VAPA and the PEDV N protein, demonstrating that VAPA binds to the N protein via its MSP domain. (B) Schematic diagrams of wild-type VAPA and its truncated mutants: VAPA-ΔMSP (MSP domain deletion), VAPA-ΔCC (coiled-coil domain deletion), and VAPA-ΔTM (transmembrane domain deletion). (C) VAPA-KO IPEC-J2 cells were transfected with empty vector, VAPA-ΔMSP, VAPA-ΔTM, or VAPA-ΔCC, followed by infection with PEDV AH2012/12 at an MOI of 0.1 for 24 h. Viral RNA levels were quantified by RT-qPCR. (D) WT IPEC-J2 cells, VAPA KO IPEC-J2 cells, and VAPA KO IPEC-J2 cells transfected with VAPA or VAPA-ΔMSP were infected with PEDV AH2012/12 at an MOI of 0.1 for 24 h. Viral RNA levels were quantified by RT-qPCR. (E) VAPA KO IPEC-J2 cells were transfected with empty vector, VAPA, VAPA-K52E, VAPA-K92E, VAPA-K94D, or VAPA-M96D, and then infected with PEDV AH2012/12 at an MOI of 0.1 for 12 h. Viral RNA levels were determined by RT-qPCR. (F and G) WT (F) and VAPA KO (G) IPEC-J2 cells were co-transfected to co-express the PEDV N protein together with WT VAPA or MSP domain point mutants. Levels of LC3, p62, and the N protein were detected by Western blotting and quantified. (H) Molecular docking analysis identified residues D404 and D405 of the PEDV N protein as key residues mediating its interaction with VAPA. (I) IPEC-J2 cells were co-transfected with VAPA together with N-His or N D404A-His plasmids. The interaction between VAPA and the N protein or N D404A mutant protein was examined by Co-IP. (J) VAPA KO IPEC-J2 cells were co-transfected with VAPA together with N-His, N D404A-His, or N D405A-His plasmids. N protein levels were detected by Western blotting and quantified. (K) Expression levels of VAPA and VAPA point mutants in WT and endogenous point-mutant IPEC-J2 cells were analyzed by Western blotting; β-actin was used as the internal control. (L) WT and VAPA endogenous point-mutant IPEC-J2 cells were infected with PEDV AH2012/12 at an MOI of 0.1 for 24 h. Viral RNA levels were quantified by RT-qPCR. All data are representative of three independent experiments and are expressed as mean ± standard error of the mean (SEM). P-values were calculated using unpaired Student’s t-tests or one-way analysis of variance (ANOVA). Statistical significance is denoted as **P < 0.01, ***P < 0.001, and ****P < 0.0001. All Western blot results are representative of at least three independent experiments.

Molecular docking predictions revealed that VAPA binds to the PEDV N protein via its MSP domain (Fig 8A). We generated three VAPA truncation mutants: VAPA-ΔMSP (MSP domain deletion), VAPA-ΔCC (coiled-coil domain deletion), and VAPA-ΔTM (transmembrane domain deletion; Fig 8B). PEDV infection assays confirmed that the MSP domain is indispensable for VAPA-mediated enhancement of PEDV infection (Fig 8C and 8D).

To pinpoint the critical amino acid residues within the MSP domain, we constructed a panel of VAPA site-directed point mutants (K52E, K92E, K94D, and M96D). Rescue infection assays in VAPA KO IPEC-J2 cells illustrated that all of these mutations completely abrogated the ability of VAPA to restore PEDV infection (Fig 8E). Further evaluation of these mutants demonstrated that the key residues in the MSP domain are essential for suppressing the autophagic degradation of the N protein in both WT and VAPA KO IPEC-J2 cells (Fig 8F and 8G).

Further docking analysis revealed that D404 and D405 within the FFAT-like motif of the N protein are critical for its interaction with VAPA (Fig 8H). Co-IP assays verified that the D404A mutation markedly attenuated the interaction between the N protein and VAPA (Fig 8I). Consistently, the N protein-stabilizing effect of VAPA was substantially diminished by the D404A or D405A mutation (Fig 8J).

In addition, we established stable IPEC-J2 cell lines carrying endogenous site-directed point mutations in VAPA (K52G/T53A and S91G/K92E; S3G Fig). These point mutations had no notable effect on VAPA expression (Fig 8K), but they significantly impaired PEDV infection (Fig 8L). Collectively, these findings demonstrate that the MSP domain of VAPA, along with its internal K52 and K92 residues, is indispensable for VAPA-mediated promotion of PEDV infection, whereas the D404 residue of the PEDV N protein serves as a key determinant governing its interaction with VAPA.

## Discussion

**Fig 9:**
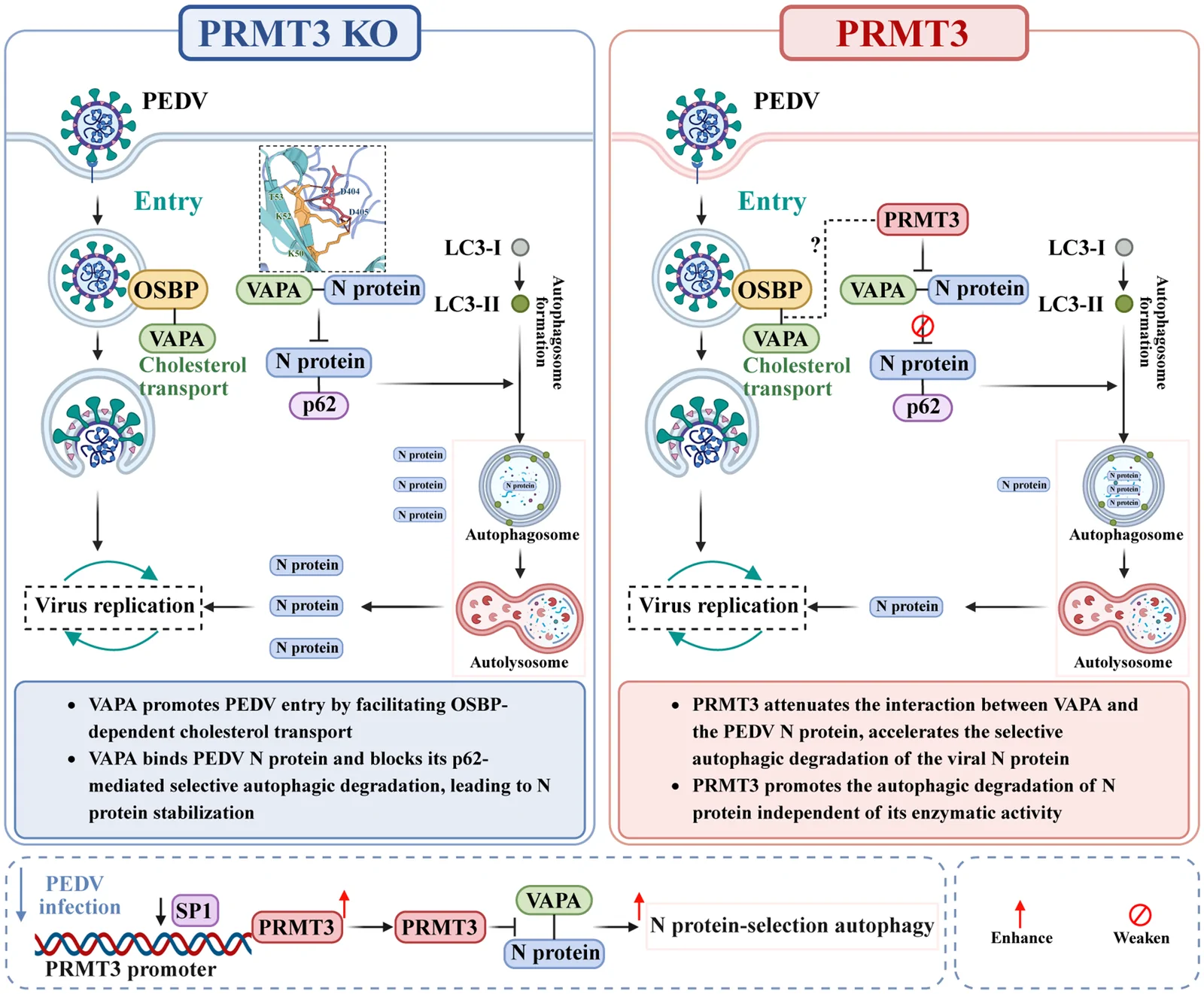
Working model of the PRMT3–VAPA–N protein selective autophagy regulatory axis governing the PEDV life cycle. PEDV infection might upregulate PRMT3 expression by downregulating the transcription factor SP1. VAPA promotes PEDV entry through OSBP-dependent cholesterol trafficking and enhances viral replication by inhibiting autophagic degradation of the viral nucleoprotein. PRMT3 antagonizes the binding between VAPA and PEDV N protein primarily through protein-protein interactions, thereby relieving the inhibitory effect of VAPA on the autophagic degradation of N protein. Fig 9 was created in BioRender. Wu, H. (2026) https://BioRender.com/5rhrt3f.

PEDV infection poses a severe threat to the global swine industry, causing exceptionally high morbidity and mortality in suckling piglets. Currently, commercially available vaccines fail to confer broad cross-protection against continuously emerging PEDV variants. The identification of critical host factors governing PEDV infection and the elucidation of underlying virus–host interaction mechanisms are therefore of great importance for PED prevention and control. In this study, we performed a genome-wide CRISPR/Cas9 knockout screen based on PEDV-induced CPEs and successfully identified *PRMT3* as a novel host restriction factor against PEDV. Mechanistic studies delineated a previously unrecognized PRMT3–VAPA–N protein selective autophagy regulatory axis, in which VAPA functions as an essential proviral host factor for PEDV pathogenesis (Fig 9). Collectively, these findings broaden our understanding of coronavirus–host interactions and provide promising molecular targets for the development of anti-PEDV therapeutics.

CRISPR-based libraries enable high-throughput, unbiased functional genomic screening, and have shown remarkable utility in identifying key genes underlying specific biological processes or diseases as well as dissecting their functional mechanisms. During screening, phenotypic readouts such as cell survival, drug resistance, cell differentiation, and migration serve as the critical bridge linking genotypes to biological functions, and form the core basis for high-throughput CRISPR library screening. In the present study, we conducted a CRISPR/Cas9 knockout screen using PEDV-induced CPEs in Vero cells as the phenotypic readout, with the primary aim of identifying regulators of virus-triggered cytopathology rather than viral replication per se. Our results showed that *PRMT3* knockout or knockdown markedly alleviated PEDV-induced CPEs in both LLC-PK1 and Vero cells. Intriguingly, PEDV infectivity was not reduced but rather significantly enhanced in *PRMT3*-deficient cells. Follow-up mechanistic studies suggested that this phenotype is associated with the regulatory role of PRMT3 in maintaining cellular autophagy homeostasis. Notably, TMEM41B, a well-documented regulator of autophagy [38,39] and viral infection [40–42], was also identified in our screen, which corroborates our findings on PRMT3 and validates the reliability of our screening pipeline. Virus-induced cytopathy is governed by diverse regulatory pathways and sophisticated molecular networks, and can be driven by multiple cell death modalities including apoptosis, autophagy, and ferroptosis. Therefore, we propose that adopting a multi-phenotype screening strategy—for instance, combining CPE-based selection with fluorescent virus-based quantification—would further improve the accuracy of genome-wide knockout screens for viral infection-related genes.

We further demonstrated that PEDV infection might downregulate the transcription factor SP1, which in turn upregulates endogenous PRMT3 transcription, forming a negative feedback loop that constrains excessive viral proliferation. SP1 is ubiquitously expressed in eukaryotic cells and regulates the transcription of approximately 6,000 genes involved in fundamental cellular processes, including cell cycle progression, metabolism, and DNA damage repair. Previous studies on viral infection have shown that the PEDV N protein interacts with SP1 to downregulate HDAC1 expression, which in turn promotes STAT1 acetylation. This cascade ultimately blocks STAT1 phosphorylation and nuclear translocation, suppresses interferon-mediated antiviral signaling, and facilitates viral replication [43]. Another study using microarray-based functional screening identified the long non-coding RNA LINC08148, which binds SP1 and upregulates Src transcription to modulate Zika virus infection [44]. Given that PEDV can efficiently invade host cells via caveolin-mediated endocytosis [45], in which Src acts as a key initiator, our findings together with previous reports indicate that SP1 regulates PEDV infection through multiple independent pathways. Accordingly, targeting SP1-associated regulatory networks holds promising potential for the development of anti-PEDV interventions.

PRMT3 is a type I protein arginine methyltransferase that catalyzes asymmetric arginine dimethylation of RIG-I, MDA5, and cGAS, thereby negatively regulating type I interferon responses and antiviral gene expression to promote infection by multiple viruses [10,11]. Here, we demonstrate for the first time that PRMT3 restricts PEDV infection primarily through the PRMT3–VAPA–N protein autophagy axis, which extends our current understanding of PRMT3-mediated viral regulation. VAPA is an endoplasmic reticulum-localized vesicle-associated membrane protein that is hijacked by numerous viruses, including hepatitis C virus and norovirus, to support viral replication via interactions with viral structural or nonstructural proteins [15–17]. In this study, we revealed that VAPA exerts dual proviral functions during PEDV infection: on one hand, it facilitates OSBP-dependent cholesterol transport in late endosomes to promote viral entry; on the other hand, it inhibits the selective autophagic degradation of the PEDV N protein, thereby maintaining N protein stability and supporting subsequent viral genome replication. Further dissection of the interaction interface identified the MSP domain of VAPA, particularly residues K52, and residue D404 within the FFAT-like motif of the PEDV N protein as the core determinants of their binding. These findings define precise molecular interfaces for antiviral intervention and provide a structural basis for the development of antiviral agents and vaccines.

In addition, base editing technology offers prominent advantages for disease-resistant breeding in pigs, including high editing precision, low off-target effects, and stable germline transmission of resistance traits. To date, this technology has been successfully applied to research on prevention and control of major swine diseases such as porcine reproductive and respiratory syndrome virus and TGEV. In this study, we generated IPEC-J2 cell lines harboring endogenous point mutations in VAPA (K52G/T53A and S91G/K92E). Functional validation showed that these mutations did not affect VAPA expression but significantly impaired PEDV infection. The identification of these critical residues provides high-precision targets for base editing-mediated disease-resistant breeding, and may also be incorporated into high-throughput breeding chips for genetic screening of virus-resistant pig breeds.

PEDV, TGEV, and PDCoV are all members of the Coronaviridae family and pose substantial threats to the global swine industry. In this study, we found that PRMT3 restricts PEDV and TGEV infection but promotes PDCoV infection. Consistently, VAPA is critical for PEDV and TGEV infection but exerts no significant effect on PDCoV proliferation. Integrating our structural interaction data with previous reports [13,46], we propose four mechanistic explanations for these divergent phenotypes. First, PDCoV employs markedly distinct interferon antagonism and immune evasion strategies relative to PEDV and TGEV. Second, PDCoV may have a lower cholesterol requirement for host cell entry than PEDV and TGEV, or may exploit alternative lipid species to facilitate cellular infection. Third, the absence of FFAT or FFAT-like motifs in the corresponding PDCoV viral proteins abrogates VAP-A binding, which in turn precludes VAP-A from modulating PDCoV replication. Fourth, VAP-A and VAP-B are structurally homologous paralogs with partially overlapping physiological functions and compensatory capacity. In contrast to PEDV and TGEV, which rely heavily on VAP-A, PDCoV exhibits greater flexibility in host factor utilization: in the absence of VAP-A, PDCoV can engage VAP-B to sustain essential processes including membrane contact formation and intracellular cargo transport. Collectively, our findings highlight profound divergence in the adaptive evolutionary strategies of PDCoV, PEDV, and TGEV, and reinforce the remarkable diversity of coronaviruses in host factor exploitation and pathogenic mechanisms. Detailed dissection of the infection mechanisms underlying diverse coronaviruses will inform our understanding of coronavirus evolution, guide the development of antiviral target repertoires with both broad-spectrum and virus-specific efficacy, and strengthen preparedness against emerging and re-emerging coronavirus outbreaks.

In addition, during the investigation into the mechanism by which PRMT3 inhibits PEDV infection in this study, it was found that knockout of either *PRMT3* or *VAPA* affects viral entry and replication. VAPA facilitates viral entry and replication through interactions with OSBP and PEDV N protein, respectively. However, this study has not yet confirmed whether the VAPA-OSBP-mediated PEDV entry process is regulated by PRMT3. Subsequent systematic and in-depth elucidation of the molecular mechanism underlying PRMT3-mediated regulation of PEDV host cell invasion will help to clarify the biological functions of PRMT3 and identify novel antiviral targets.

In summary, our study identifies *PRMT3* as a host restriction factor for PEDV infection and establishes VAPA as an essential proviral host factor required for efficient PEDV propagation. VAPA promotes PEDV entry through OSBP-dependent cholesterol trafficking and enhances viral replication by inhibiting autophagic degradation of the viral nucleoprotein. The newly defined PRMT3–VAPA–N protein selective autophagy regulatory axis advances our understanding of PEDV–host interactions and expands the known biological functions of PRMT3. These findings also provide actionable therapeutic targets and experimental evidence for the development of PED prevention and control strategies.

## Materials and methods

### Cells and viruses

Vero, HEK293T, PK15, LLC-PK1, and IPEC-J2 cells were maintained in the laboratory and cultured in Dulbecco’s Modified Eagle Medium (DMEM; Gibco, USA) supplemented with 10% fetal bovine serum (FBS; Sigma-Aldrich, USA) at 37°C with 5% CO_2_. LLC-PK1 and IPEC-J2 cells were kindly provided by Prof. Bin Li (Institute of Veterinary Medicine, Jiangsu Academy of Agricultural Sciences).

Primary porcine intestinal epithelial cells were isolated and cultured as previously described [47]. Colostrum‑deprived Landrace piglets were humanely euthanized, and their small intestines were aseptically harvested and dissected into 10‑cm segments. The intestinal segments were washed twice with antibiotic‑containing PBS to thoroughly remove intraluminal mucus. One end of each segment was ligated with sterile cotton thread, and the lumen was filled with type II collagenase solution (800 U/mL; Sigma‑Aldrich), followed by enzymatic digestion at 37 °C for approximately 5 min. Following digestion, the luminal cell suspension was collected and centrifuged at 400 × g for 8 min at 4 °C. The resulting cell pellet was resuspended in DMEM‑10 medium (DMEM supplemented with 10% FBS), repelleted by centrifugation, and washed twice with DMEM‑10 medium. Finally, the cells were seeded and maintained in EGF‑10 medium (epidermal growth factor‑supplemented medium containing 10% FBS). Cell identity and purity were validated by flow cytometric detection of the epithelial marker gene Villin and RT‑qPCR analysis of additional epithelium‑specific markers. All primary porcine intestinal epithelial cells used in this study were cultured for no more than 10 passages.

PEDV strains AH2012/12 (GenBank: KU646831) and PDCoV strain CZ2020 (GenBank: OK546242) were kindly provided by Prof. Bin Li and preserved in the laboratory. PEDV strain LW/L (GenBank: MK392335.1), TGEV strain SD/L, and PRV strain BarthaK61 (GenBank: JF797217.1) were preserved in the laboratory. The PRV-EGFP recombinant strain was constructed as previously described [48].

### Antibodies and reagents

Antibodies against PRMT3 (17628–1-AP), VAPA (15275–1-AP), His Tag (66005–1-Ig), Flag Tag (66008–4-Ig), LC3 (14600–1-AP), Villin (16488–1-AP), Rab7A (55469–1-AP), and p62/SQSTM1 (18420–1-AP) were purchased from Proteintech. Anti-β-actin (BM0627), HRP-conjugated AffiniPure goat anti-rabbit/mouse IgG (BA1055, BA1050), and DyLight 488/550-conjugated secondary antibodies (BA1127, BA1126, BA1133, BA1135) were obtained from BOSTER. Rabbit IgG (A7016), mouse IgG (A7028), Ad-mCherry-GFP-LC3B (C3011), and Protein A + G Magnetic Beads (P2108) were purchased from Beyotime. Anti-PEDV N antibody (DA0110) for IFA was obtained from YouLong Biotech (Shanghai, China). Poly(I:C) HMW (tlrl-pic) was purchased from InvivoGen. SGC707 (HY-19715), 25-hydroxycholesterol (25-HC; HY-113134), MβCD (HY-101461), bafilomycin A1 (BafA1; HY-100558), chloroquine (CQ; HY-17589A), and MG132 (HY-13259) were purchased from MCE. PRMT3-specific siRNAs were synthesized by Sangon Biotech (Shanghai, China); sequences are listed in S1 Table.

### Plasmids and transfection

All plasmids used in this study were constructed as detailed below. PRMT3 and VAPA overexpression plasmids were generated by PCR amplification from porcine cDNA, followed by ligation into the pB510B eukaryotic expression vector. Flag‑ and his‑tagged PRMT3, His‑tagged VAPA, and His‑tagged N plasmids were constructed and inserted into the pB510B vector using the same strategy. VAPA site-directed mutant plasmids harboring K52E, K92E, K94D, or M96D substitutions, as well as PEDV N protein site-directed mutant plasmids carrying D404A or D405A mutations, were constructed using the FAST Site-Directed Mutagenesis Kit (TIANGEN, KM101). The full‑length and truncated fragments of the PRMT3 promoter were cloned into the pGL4.1 luciferase reporter vector. sgRNAs targeting PRMT3 and VAPA were cloned into the PX459 plasmid or pBluescriptSKII+ U6-sgRNA(F + E) plasmid.

Transient cell transfection was performed using jetPRIME transfection reagent (Polyplus, 101000001). The Neon Transfection System (Thermo Scientific, Cat. No. MPK10096) was used for transfection to generate gene-knockout cell lines via SpCas9 and endogenously point-mutated cell lines via ABE8e, respectively. Library‑enriched sgRNAs targeting green monkey genes were subcloned into the lentiCRISPR v2 vector [49]. The lentiCRISPR v2 vector was a kind gift from Feng Zhang (Addgene plasmid #52961). The green monkey (*Chlorocebus sabaeus*) genome‑scale sgRNA library was provided by John Doench and David Root [50] (Addgene #178284). All primer pairs used for plasmid construction are summarized in S1 Table.

### Lentiviral production, cell library construction, and PEDV screening

Lentiviral library production was performed as follows. For each 100-mm culture dish, 6 μg of the green monkey genome-wide CRISPR/Cas9 knockout (GeCKO) library plasmid, 5 μg of psPAX2, and 4 μg of pMD2.G were diluted in 0.5 mL Opti-MEM. A total of 20 independent 100-mm culture dishes were prepared, with 120 μg of GeCKO library plasmid used in total. In parallel, 45 μL of polyetherimide transfection reagent was diluted in 0.5 mL of Opti-MEM. The two solutions were mixed gently and incubated at room temperature for 15–20 minutes. The transfection mixture was then added dropwise to the cell culture dishes. At 12 hours post-transfection, the medium containing the transfection complex was removed and replaced with 10–12 mL of complete culture medium supplemented with 10% FBS. After 60 hours of incubation, the viral supernatant was harvested and centrifuged at 2000 rpm for 5 minutes at 4°C to remove large cellular debris. The supernatant was filtered through a 0.22 μm filter to eliminate residual cell debris and metabolic waste, then aliquoted and stored at −80°C until use.

To construct the green monkey GeCKO cell library, Vero‑Cas9 cells (Vero cells stably expressing Cas9) were seeded at 40% confluence in 10 × 150 mm dishes. The green monkey GeCKO lentiviral library was mixed 1:1 with DMEM containing 8 μg/mL polybrene (Sigma‑Aldrich, #TR‑1003) and used to transduce the cells. The viral volume was adjusted to ensure an MOI ≤ 0.3 and at least 500 cells were represented per sgRNA. After 72 h of incubation, transduced cells were selected in medium supplemented with 5 μg/mL puromycin (Solarbio, P8230) for 7 days, followed by a 7‑day recovery period.

For PEDV screening, the GeCKO cell library was infected with PEDV LW/L in DMEM supplemented with 10 μg/mL trypsin, and incubated at 37°C with 5% CO_2_. Surviving cells were collected 3 days post‑infection, expanded, and used for the next round of viral challenge. After four rounds of screening in three independent screening experiments, surviving cells were collected, and sgRNA amplicons were constructed for deep sequencing analysis.

For deep sequencing analysis, maintain a sequencing depth of at least 500× for sequencing data during sequencing. Raw sequencing data were subjected to md5sum verification to validate data integrity and then decompressed. The names and sequences of sgRNAs were extracted using the awk command and formatted into a non-redundant FASTA library. The sgRNA library was indexed with Bowtie 2, and paired-end reads were merged using FLASH software, followed by trimming to the 20-bp core sgRNA region with Cutadapt. The MAGeCK count and test modules were applied to quantify sgRNA read counts and analyze differential abundance between groups. volcano plots were generated on the online bioinformatics platform (https://www.bioinformatics.com.cn) to visualize the library screening results.

### Generation of candidate gene-KO Vero and transcription factor-KO IPEC-J2 cell lines

sgRNAs targeting candidate genes and transcription factors were subcloned into the lentiCRISPR v2 vector, and lentivirus packaging was performed following the aforementioned experimental procedures. The harvested sgRNA-expressing lentiviruses were separately used to infect Vero cells and IPEC-J2 cells for 3 days, followed by 7 consecutive days of puromycin selection to generate polyclonal stable knockout cell pools. Comparative analysis of sequencing chromatograms and quantitative measurement of indel mutation frequencies between wild-type cells, candidate gene-knockout Vero cells and transcription factor-knockout IPEC-J2 cells are summarized in Tables S1 and S2. The quantitative analysis of indel mutation frequencies was conducted with the TIDE (Tracking of Indels by DEcomposition) tool [51].

### CellTiter-glo luminescent cell viability assay

Vero cells transduced with non-targeting or gene-specific sgRNAs were infected with PEDV LW/L (MOI = 0.01) for 60 h. Cell viability was measured using the CellTiter-Glo Luminescent Cell Viability Assay according to the manufacturer’s instructions, and luminescence was detected using a microplate reader. Cell viability was calculated as (virus-infected group − blank)/(uninfected group − blank).

### Luciferase reporter assay

HEK293T cells were seeded into 24-well plates and transfected using polyetherimide. Cells were co-transfected with the PRMT3 promoter luciferase reporter plasmid, pRL-TK internal control vector, and the indicated expression plasmids. At 48 h post-transfection, cells were harvested and lysed. Firefly and Renilla luciferase activities were measured using a dual-luciferase reporter assay kit (Beyotime, RG027) following the manufacturer’s instructions. Luciferase activity was normalized to Renilla luciferase activity as an internal control. Primer pairs used for plasmid construction are listed in S1 Table.

### IFA

Cells were seeded into 24‑well plates. At 24 h post‑transfection or post‑infection, cells were fixed with 4% paraformaldehyde (AR1068, BOSTER) at room temperature for 30 min. Subsequently, cells were permeabilized with 0.1% Triton X‑100 (T8200, Solarbio) at room temperature for 10 min. Cells were then incubated with primary antibodies at room temperature for 1 h, followed by three washes with PBS. Next, cells were incubated with fluorescently labeled secondary antibodies at room temperature for 1 h in the dark. Finally, cell nuclei were counterstained with DAPI (C1002, Beyotime). Fluorescent images were acquired using an EVOS f1 fluorescence microscope and an LSM 900 confocal laser scanning microscope.

For cholesterol staining, cells were stained in accordance with the instructions of the Cholesterol Blue Fluorescence Assay Kit with Filipin Complex (Beyotime, C2063S). The stained samples were imaged and photographed using a Nikon C2 Plus laser scanning confocal microscope.

### WB

Cells were lysed in cell lysis buffer (Beyotime, P0013) containing phenylmethanesulfonyl fluoride (PMSF, Beyotime, ST506), centrifuged at 12,000 rpm for 15 min at 4 °C, and boiled with 5 × SDS loading buffer. Proteins were separated by SDS-PAGE and transferred to PVDF membranes. Membranes were blocked with 5% skim milk for 2 h, incubated with primary antibodies overnight at 4 °C, and then with HRP-conjugated secondary antibodies for 1 h at room temperature. Protein bands were visualized using an ECL chemiluminescent kit (BOSTER, AR1197) and the Hesper II Chemiluminescence Imaging System (Monad Biotech). Band intensity was quantified using Fiji ImageJ.

### Co-IP assay

HEK293T, IPEC-J2, or LLC-PK1 cells were co-transfected with the indicated plasmids and cultured for 48 h. Cells were then lysed in cell lysis buffer (Beyotime, P0013) supplemented with PMSF. The protein lysates were incubated with specific antibodies (3 μg of antibody per 2 mg of total cellular protein) overnight at 4 °C. Next, 20 μL of Protein A + G magnetic beads (Beyotime, P2108) were added to the antibody–protein mixture and incubated at room temperature for 1 h to capture antibody–protein complexes. After washing five times with Tris-buffered saline, the magnetic beads were boiled in SDS loading buffer to elute bound proteins. The magnetic beads were removed, and co-precipitated proteins were detected by Western blotting.

### Autophagy experiment

Western blot analysis: IPEC-J2 and other indicated cell lines were transfected to express the PEDV N protein and target proteins, or infected with PEDV for 24 h. Cells were subsequently treated with 50 μM CQ or DMSO for 9 h. Total cellular protein was extracted, and the expression levels of target proteins, p62 and LC3 were detected by Western blot.

mCherry-GFP-LC3 autophagic flux assay: IPEC-J2 and indicated cells were infected with Ad-mCherry-GFP-LC3B at an MOI of 10 for 12 h, followed by transfection with plasmids encoding the indicated proteins for 24 h. Cells were then subjected to PEDV infection or mock infection for another 24 h. After treatment with 50 μM CQ or DMSO for 9 h, cells were fixed and visualized using an LSM 900 confocal laser scanning microscope. Autophagosome puncta per cell was manually counted from ≥3 random visual fields per sample to calculate autophagic flux level.

### RNA isolation and RT-qPCR

Total RNA was extracted using the RNAsimple Total RNA Kit (TIANGEN, DP419) and reverse-transcribed into cDNA using the FastKing gDNA Dispelling RT SuperMix (TIANGEN, KR118). RT-qPCR was performed using the Talent qPCR PreMix (SYBR Green; TIANGEN, FP209), and relative gene expression was calculated using the 2^(-ΔΔCt) method with *GAPDH* as the internal reference. Primer sequences are listed in S1 Table.

### Crystal Violet assay and TCID_50_ assay

For the crystal violet staining assay, confluent cell monolayers in 6‑well plates were infected with the indicated virus until obvious CPE developed. Infected cells were fixed with 4% paraformaldehyde at room temperature for 30 min, washed twice with distilled water, and then stained with crystal violet staining solution (Beyotime, ST506) for 10 min. After extensive washing with distilled water, cells were air‑dried and subjected to observation and imaging.

For the TCID₅₀ assay, cells were seeded at a density of 1 × 10⁴ cells per well in 96-well plates. Collected viral supernatants and cell suspensions were subjected to multiple freeze–thaw cycles, and the resulting viral preparations were used to inoculate the confluent cell monolayers. Cells were incubated for 3–5 days until prominent CPE was observed under an optical microscope. TCID₅₀ titers were calculated using the Reed–Muench method [52]. Viral titers of PEDV, TGEV, PDCoV, and PRV were determined in Vero, LLC-PK1, and PK15 cells, respectively.

### Statistical analysis

Statistical analyses were performed using GraphPad Prism 5.0 software. All data are representative of at least three independent experiments and are presented as the mean ± standard error of the mean (SEM). P-values were calculated using unpaired Student’s t-tests or one-way analysis of variance (ANOVA). A P-value < 0.05 was considered statistically significant.

## Supporting information

S1 Fig (A) LLC‑PK1 and IPEC‑J2 cells were transfected with non‑targeting control siRNA (siNC) or PRMT3‑specific siRNA. PRMT3 mRNA levels were quantified by RT‑qPCR. (B) Morphological comparison of isolated primary porcine intestinal epithelial cells (IPEC-WW) and IPEC‑J2 cells under light microscopy. Scale bar, 100 µm. (C) Expression of Villin proteins in IPEC‑J2 and primary IPEC-WW cells was detected by flow cytometry. (D) RT-qPCR was performed to quantify the mRNA expression of epithelial marker genes including EPCAM, Villin, E-cadherin, and KRT8 in IPEC-J2 and IPEC-WW cells, with GAPDH used as the internal reference. (E) Schematic diagram illustrating the generation of stable PRMT3 OE LLC‑PK1 and IPEC‑J2 cell lines using the PiggyBac transposon system. (F) WT and PRMT3 OE LLC‑PK1 cells were infected with PEDV LW/L at MOIs of 0.01 and 0.05 for 24 h. Viral RNA levels were quantified by RT‑qPCR. (G) WT and PRMT3 OE LLC‑PK1 cells were infected with PEDV LW/L (MOI = 0.01) for 24 h. PEDV N protein expression was detected and quantified by IFA. Scale bar, 100 µm. (H) LLC‑PK1 cells were transfected with wild‑type PRMT3 or its enzymatically inactive mutant PRMT3-3A (G263A/C264A/G265A), followed by infection with PEDV AH2012/12 at an MOI of 0.1 for 24 h. Viral RNA levels were quantified by RT‑qPCR. PRMT3 and PRMT3-3A expression was detected by Western blotting. (I) Schematic workflow of PRMT3‑His IP‑MS in LLC‑PK1 cells. (J) Western blotting analysis of PRMT3‑His expression in PRMT3 KO LLC‑PK1 cells; β‑actin was used as the internal control. (K) Validation of PRMT3‑His immunoprecipitation by Western blotting using an anti‑His antibody. (L) Gene Ontology biological process enrichment analysis of candidate PRMT3‑interacting proteins. All data are representative of three independent experiments and are expressed as mean ± standard error of the mean (SEM). P-values were calculated using unpaired Student’s t-tests or one-way analysis of variance (ANOVA). Statistical significance is denoted as **P < 0.01, ***P < 0.001, and ****P < 0.0001. All Western blot results are representative of at least three independent experiments.
(TIF)

S2 Fig (A) Kyoto Encyclopedia of Genes and Genomes pathway enrichment analysis of candidate PRMT3-interacting proteins. (B) HEK293T cells were co-transfected with PRMT3-Flag and VAPA-His plasmids. The interaction between PRMT3 and VAPA was validated by reciprocal Co-IP using an anti-His antibody. (C) HEK293T cells were co-transfected with PRMT3-Flag and VAPA-His plasmids. Intracellular co-localization of PRMT3 and VAPA was visualized by immunofluorescence microscopy. Scale bar, 20 µm. (D and E) WT and VAPA KO IPEC-J2 cells were infected with PDCoV strain CZ2020 (MOI = 0.1) (D) or PRV strain BarthaK61 (MOI = 0.001) (E) for 24 h. Viral RNA levels and titers were quantified by RT-qPCR and TCID₅₀ assay, respectively. (F) Expression levels of PRMT3 and VAPA in mock-infected and PEDV LW/L-infected WT, PRMT3-KO, and PRMT3 OE LLC-PK1 cells were detected by Western blotting. (G) IPEC-J2 cells were infected with PEDV AH2012/12 at an MOI of 0.1 for 2 h, followed by treatment with 25-HC or DMSO for an additional 22 h. The interaction between VAPA and OSBP was examined by Co-IP. (H) Following 4 h treatment with DMSO or MβCD, WT and VAPA KO cells were fixed and co-stained with filipin (cyan) and Rab7 (red). Fluorescent images were acquired under a fluorescence microscope. Scale bar, 20 µm. (I) IPEC-J2 cells were transfected with Nsp5-His plasmid. The interaction between endogenous VAPA and PEDV Nsp5 protein was assessed by Co-IP. All data are representative of three independent experiments and are expressed as mean ± standard error of the mean (SEM). P-values were calculated using unpaired Student’s t-tests or one-way analysis of variance (ANOVA). Statistical significance is denoted as ns: not significant (P > 0.05). All Western blot results are representative of at least three independent experiments.
(TIF)

S3 Fig (A) IPEC-J2 cells were co-transfected with N-His and VAPA expression plasmids. The protein levels of N protein, total mTOR, and phosphorylated mTOR (p-mTOR) were determined by Western blotting and quantified by band densitometry. (B) IPEC-J2 cells were co-transfected with N-His and VAPA plasmids, then treated with 50 μM CQ for 9 h. Co-IP assays were performed to examine the effect of VAPA on the interaction between N-His and endogenous p62 protein. (C) IPEC-J2 cells were co-transfected with N-His along with wild-type PRMT3 or its catalytically inactive mutant PRMT3-3A (G263A/C264A/G265A), followed by treatment with 50 μM CQ or DMSO for 9 h. The protein levels of LC3 and PEDV N protein were detected by Western blotting and subjected to quantitative analysis. (D) IPEC-J2 cells were transduced with Ad-mCherry-GFP-LC3B adenovirus, followed by transfection with empty vector, N-His, or N-His + PRMT3 plasmids. After 9 h of treatment with 50 μM CQ or DMSO, autophagic flux was visualized by fluorescence microscopy. Scale bar, 20 μm. The numbers of intracellular red and yellow fluorescent puncta were quantified to evaluate autophagic flux. (E) WT and PRMT3 KO LLC-PK1 cells were mock-infected or infected with PEDV LW/L at an MOI of 0.1 for 24 h. The protein levels of LC3 and p62 were determined by Western blotting, quantified, and normalized to those in mock-infected controls. (F) IPEC-J2 cells were transduced with Ad-mCherry-GFP-LC3B adenovirus and then transfected with empty vector or PRMT3 plasmids. Cells were subsequently mock-infected or infected with PEDV for an additional 24 h. Following 9 h of treatment with 50 μM CQ or DMSO, autophagic flux was examined by fluorescence microscopy. Scale bar, 20 μm. The numbers of intracellular red and yellow fluorescent puncta were quantified to assess autophagic flux. (G) Comparison of Sanger sequencing chromatograms between wild-type IPEC-J2 cells and IPEC-J2 cells harboring endogenous VAPA point mutations. Autophagosome dots in each cell were counted by selecting at least three random fields of view per sample to assess the level of autophagic flux, and the results were presented as mean ± standard error of the mean (SEM). P-values were calculated using unpaired Student’s t-tests. Statistical significance is denoted as ns: not significant (P > 0.05) and ****P < 0.0001. All Western blot results are representative of at least three independent experiments.
(TIF)

S1 Table Primer pairs, sgRNA targeting sequences, and siRNAs used in this study. (XLSX)

S2 Table Sequencing results of genes in the MOI = 0.01 Vero KO library screen. (XLSX)

S3 Table Sequencing results of genes in the MOI = 0.1 Vero KO library screen. (XLSX)

S4 Table The protein quantification data results for PRMT3 IP-MS. (XLSX)

S5 Table Comparison of sequencing chromatograms between wild‑type and candidate gene-KO Vero cells and quantification of indel frequency. (XLSX)

S6 Table Comparison of sequencing chromatograms between wild‑type and transcription factor‑knockout IPEC‑J2 cells and quantification of indel frequency. (XLSX)

S7 Table Quantification from independent experiments for key immunoblot data supporting major conclusions. (XLSX)

S1 File S1 Rawgel. Uncropped original western blot images.
(PDF)
