# Supplementary material for: PRMT3 restricts porcine epidemic diarrhea virus replication by disrupting the interaction between VAPA and the viral nucleocapsid protein
Source: PLoS Pathog. 2026 Sep 8;22(9):e1014599. doi: 10.1371/journal.ppat.1014599 (PMC13577570; doi:10.1371/journal.ppat.1014599)
Supplement: Supplementary file 1 [file ppat.1014599.s011.pdf]

Fig 1E

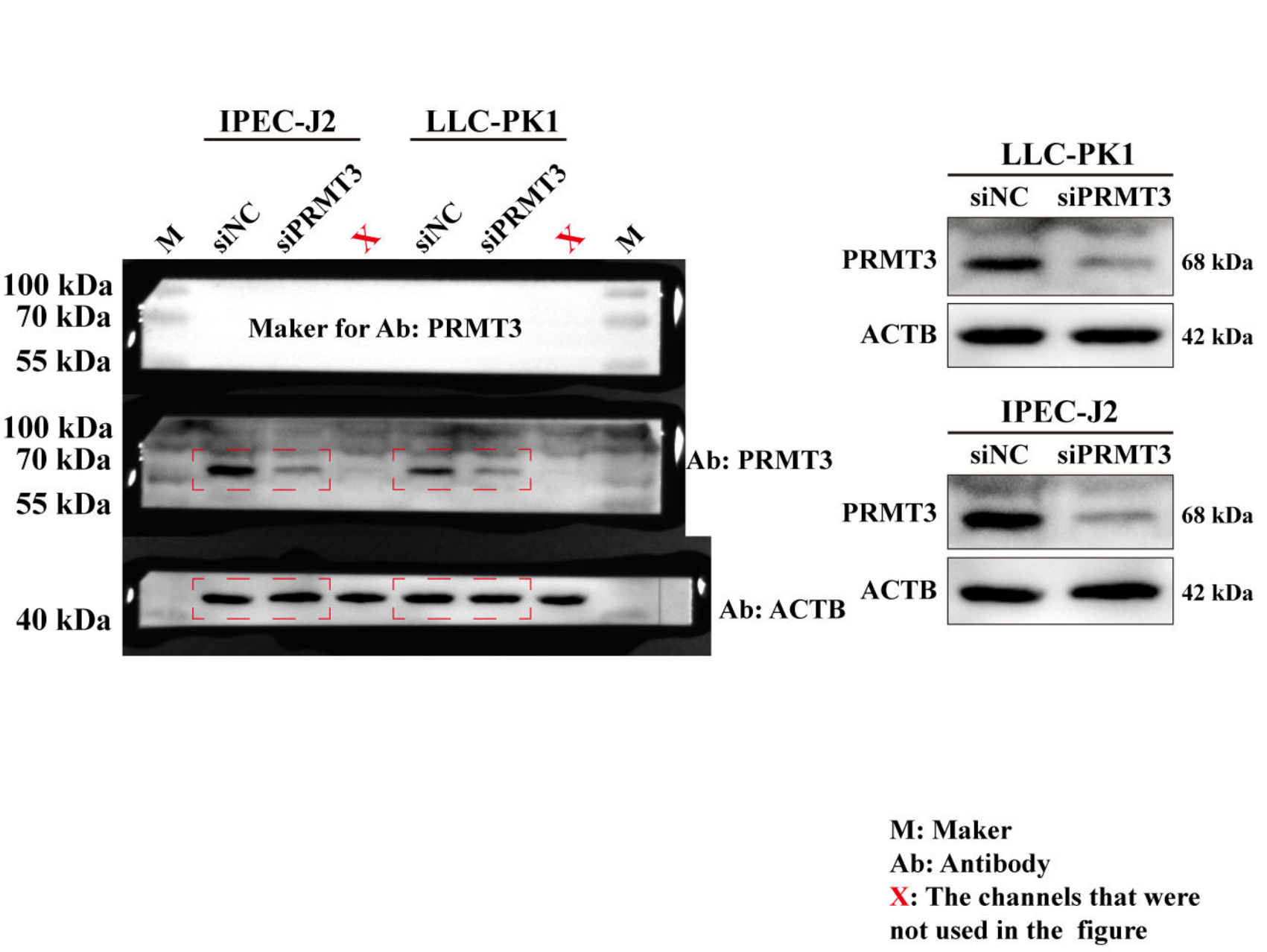

Fig 2A

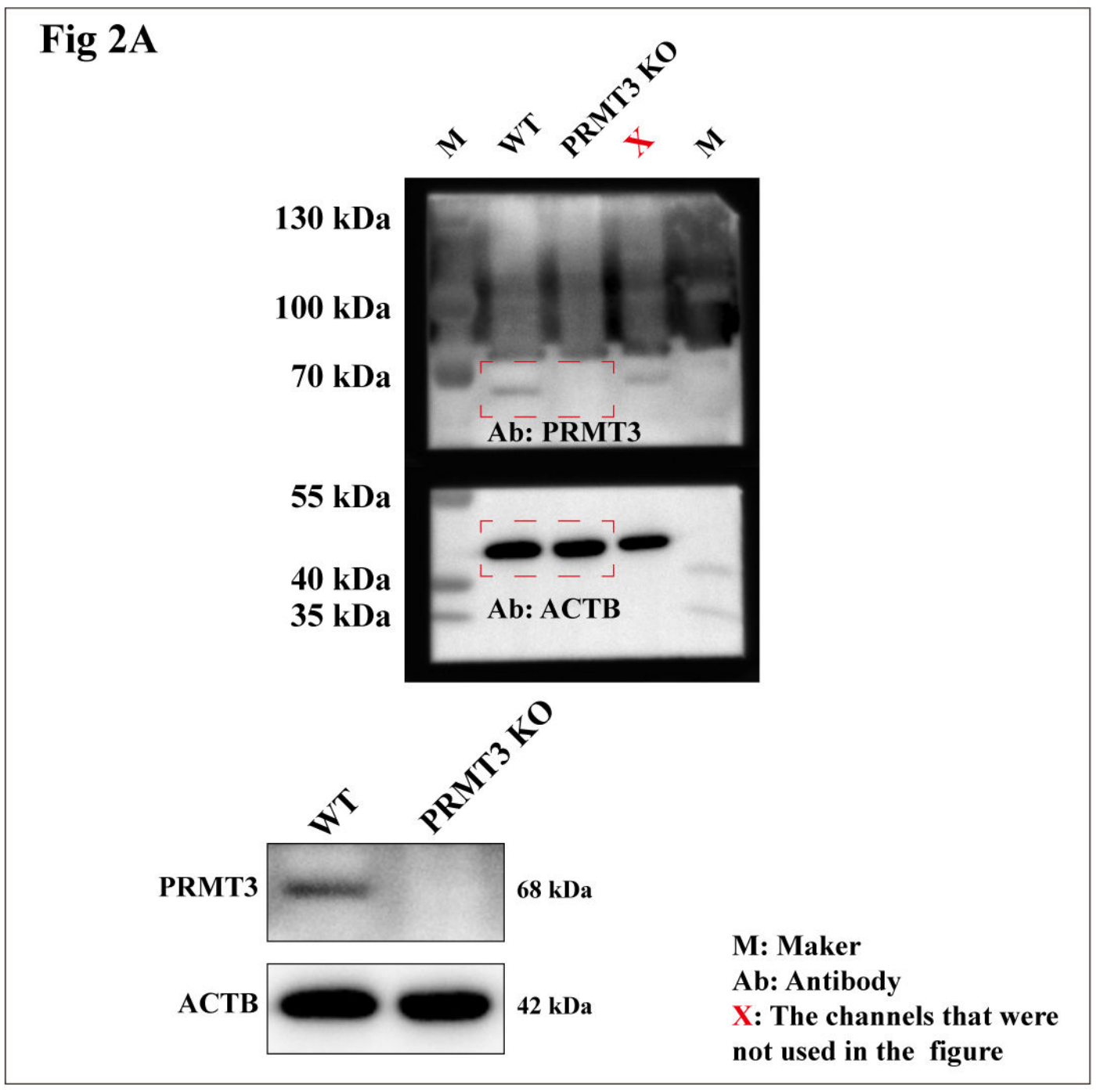

Fig 2I

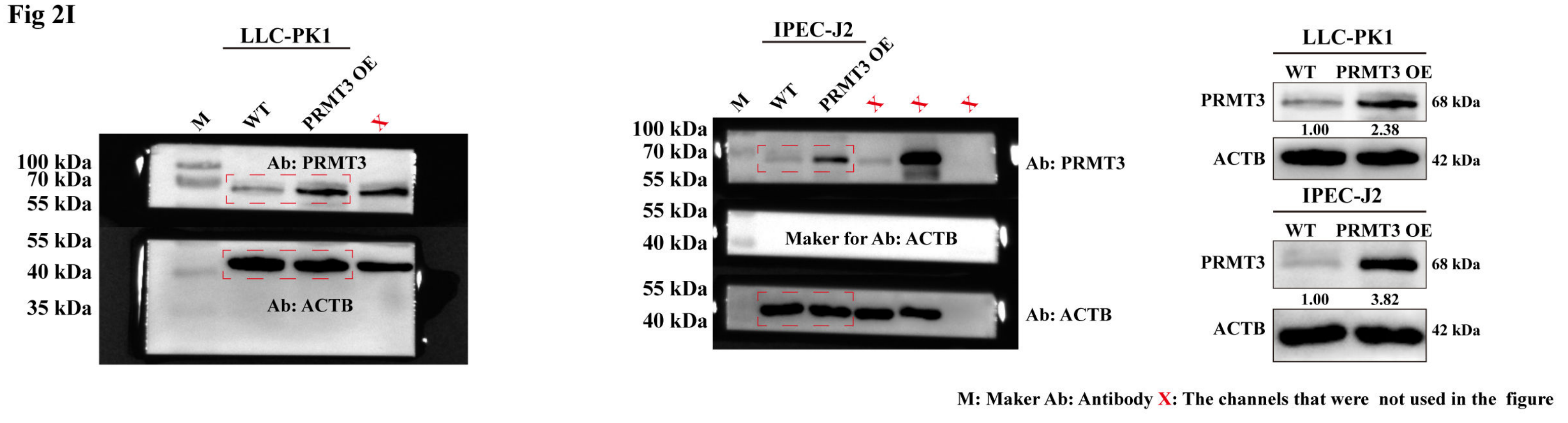

Fig 2N

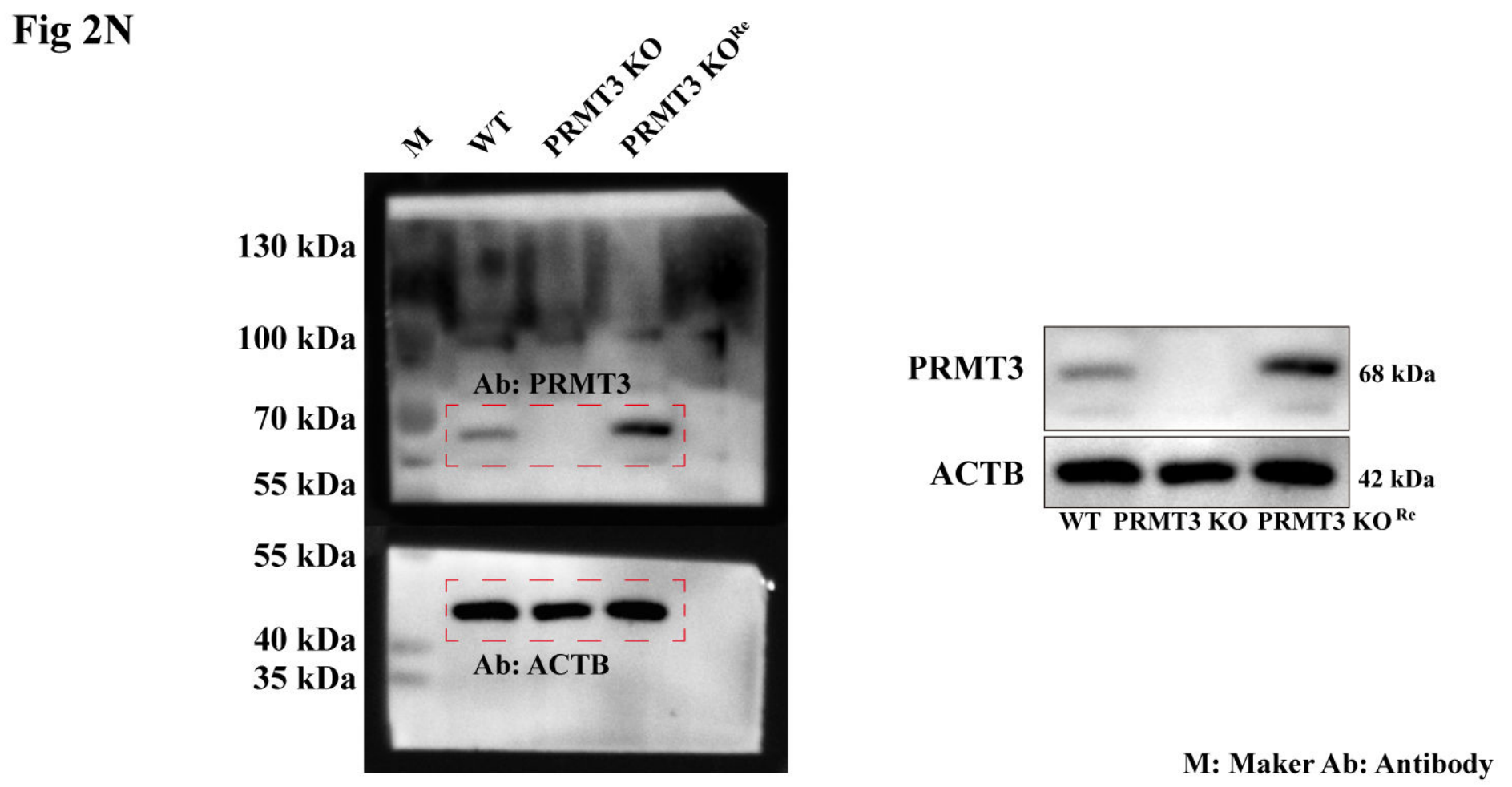

Fig 3A

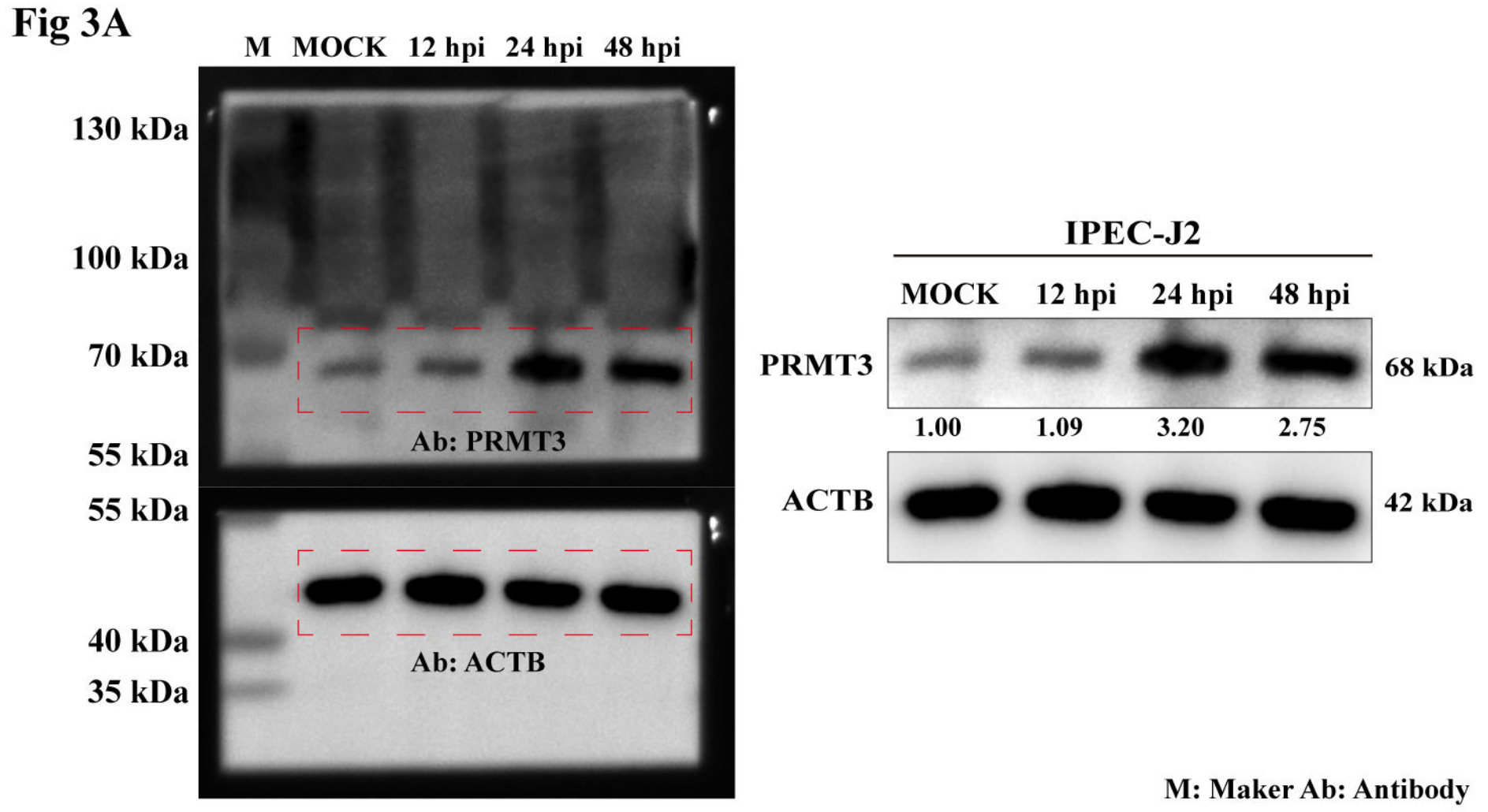

Fig 3B

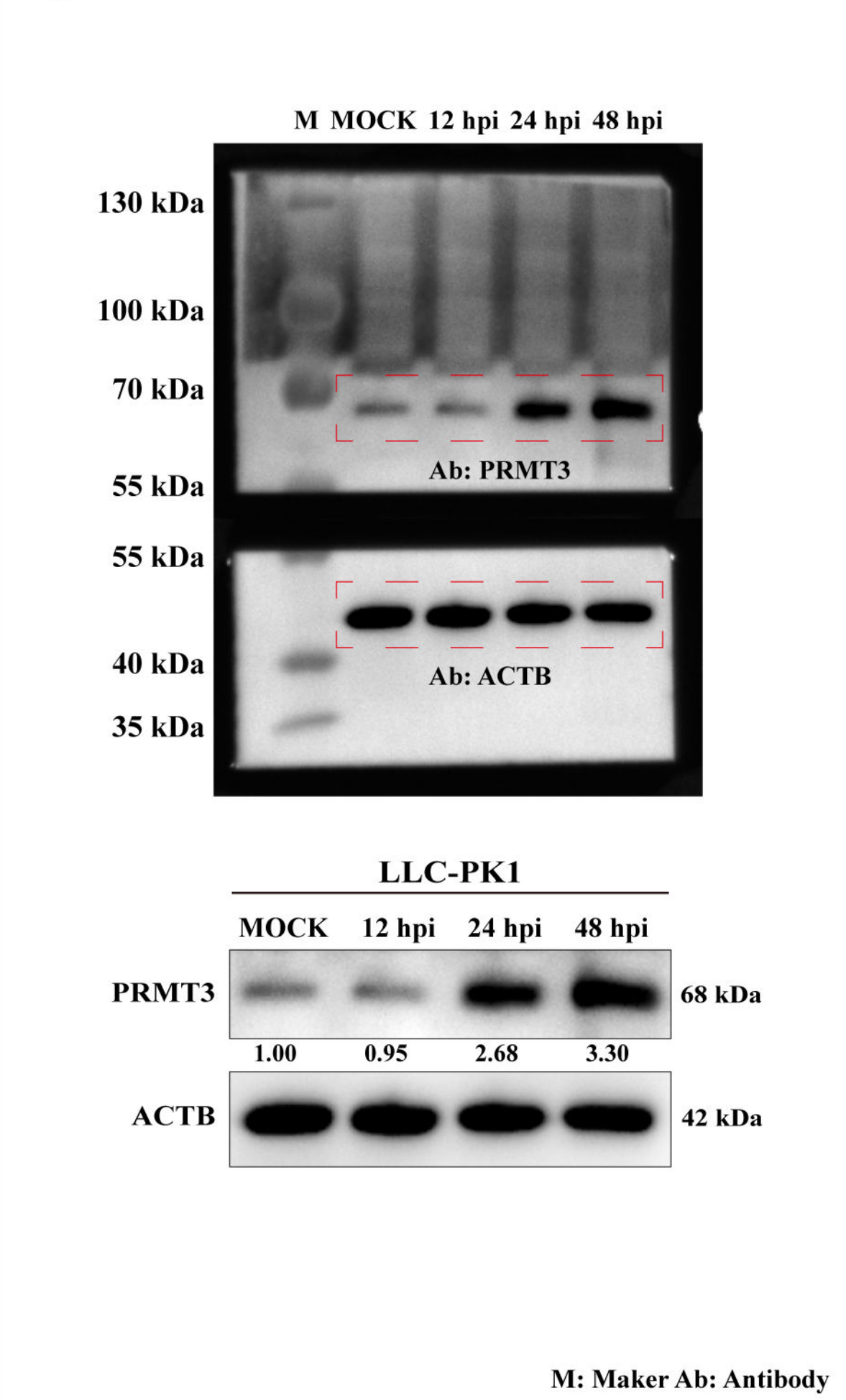

Fig 5B

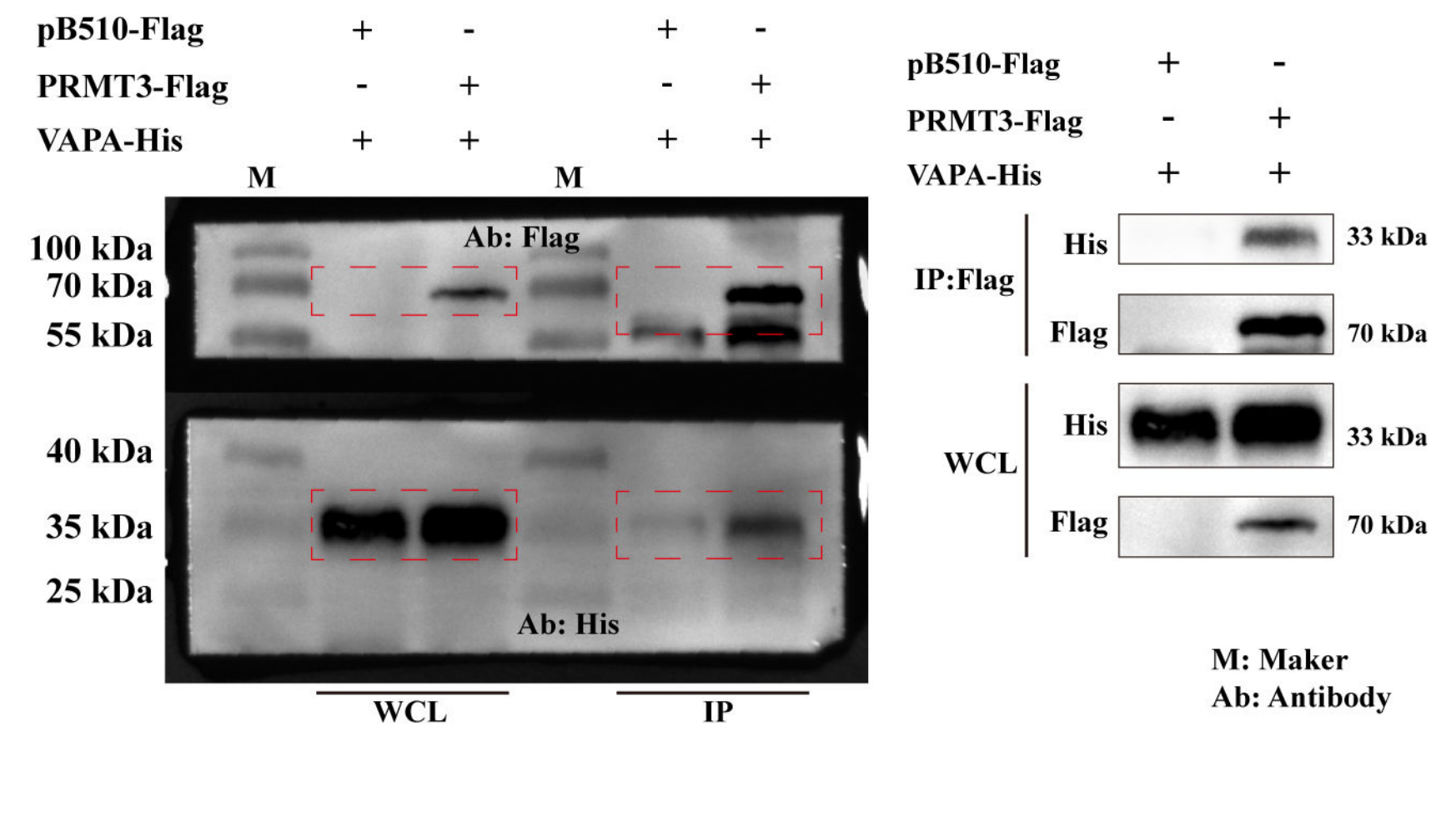

Fig 5C

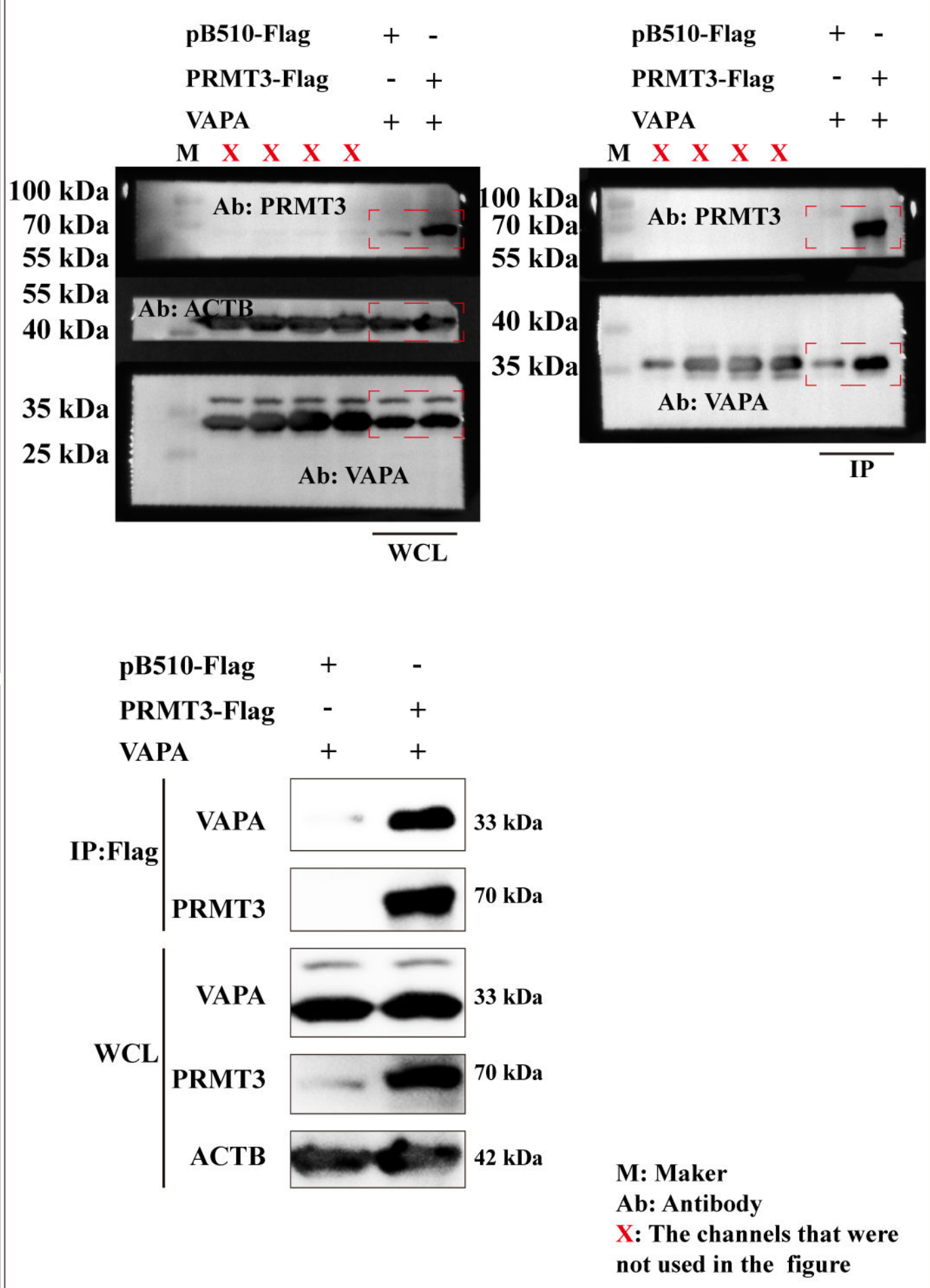

Fig 5D

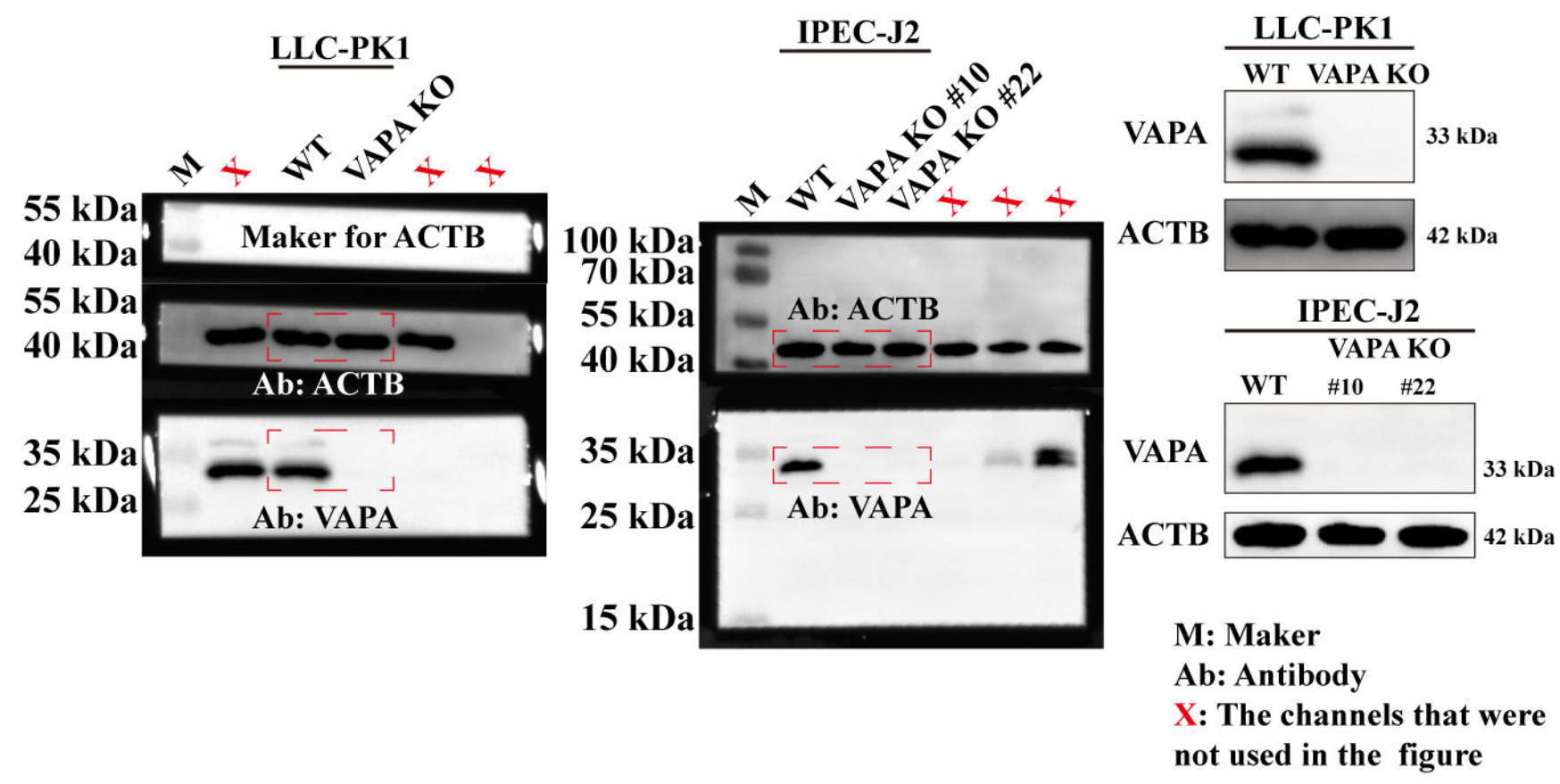

Fig 5K

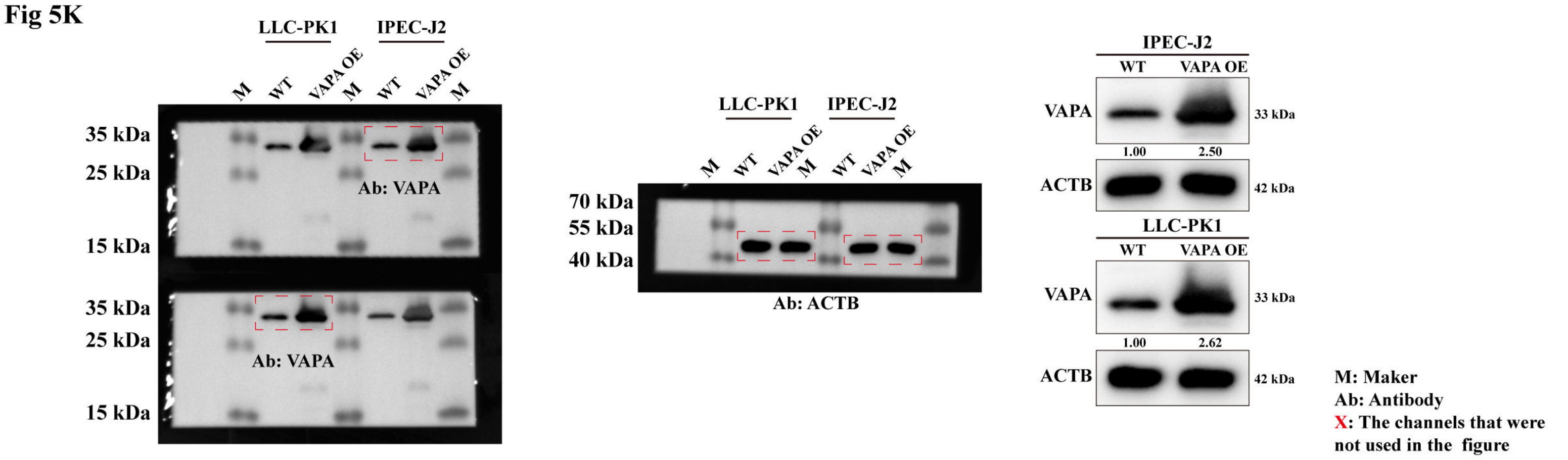

Fig 5M

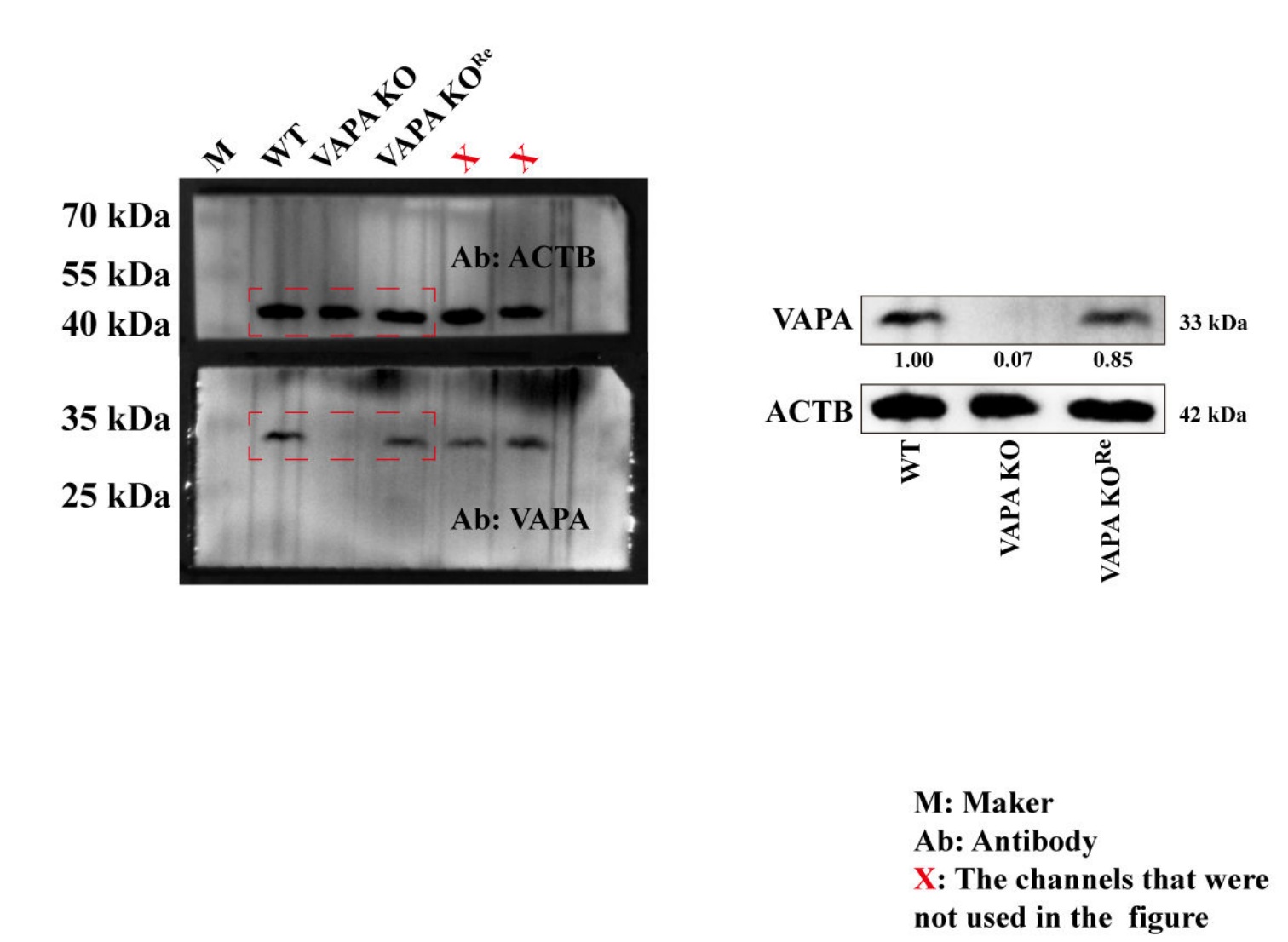

Fig 5N

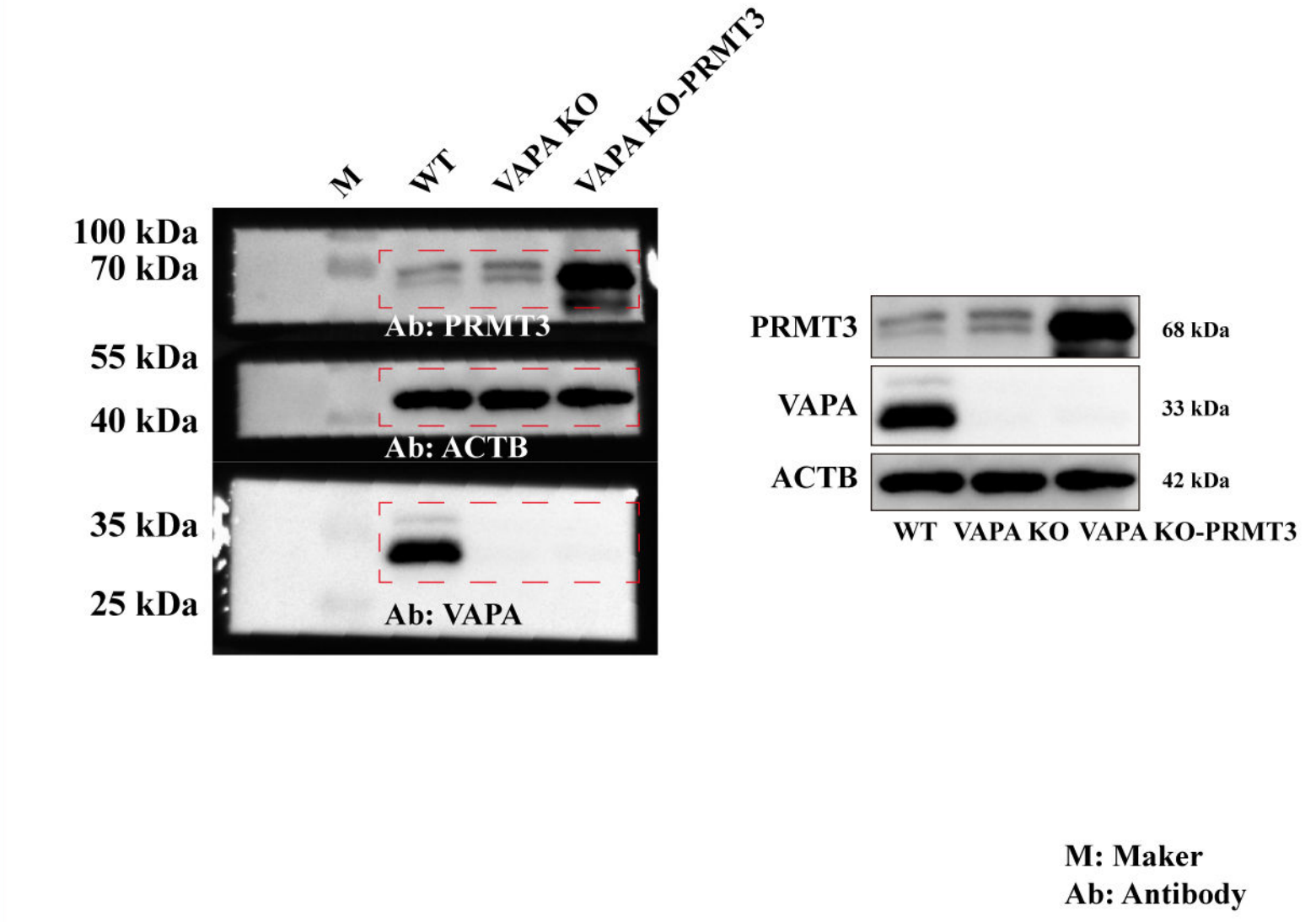

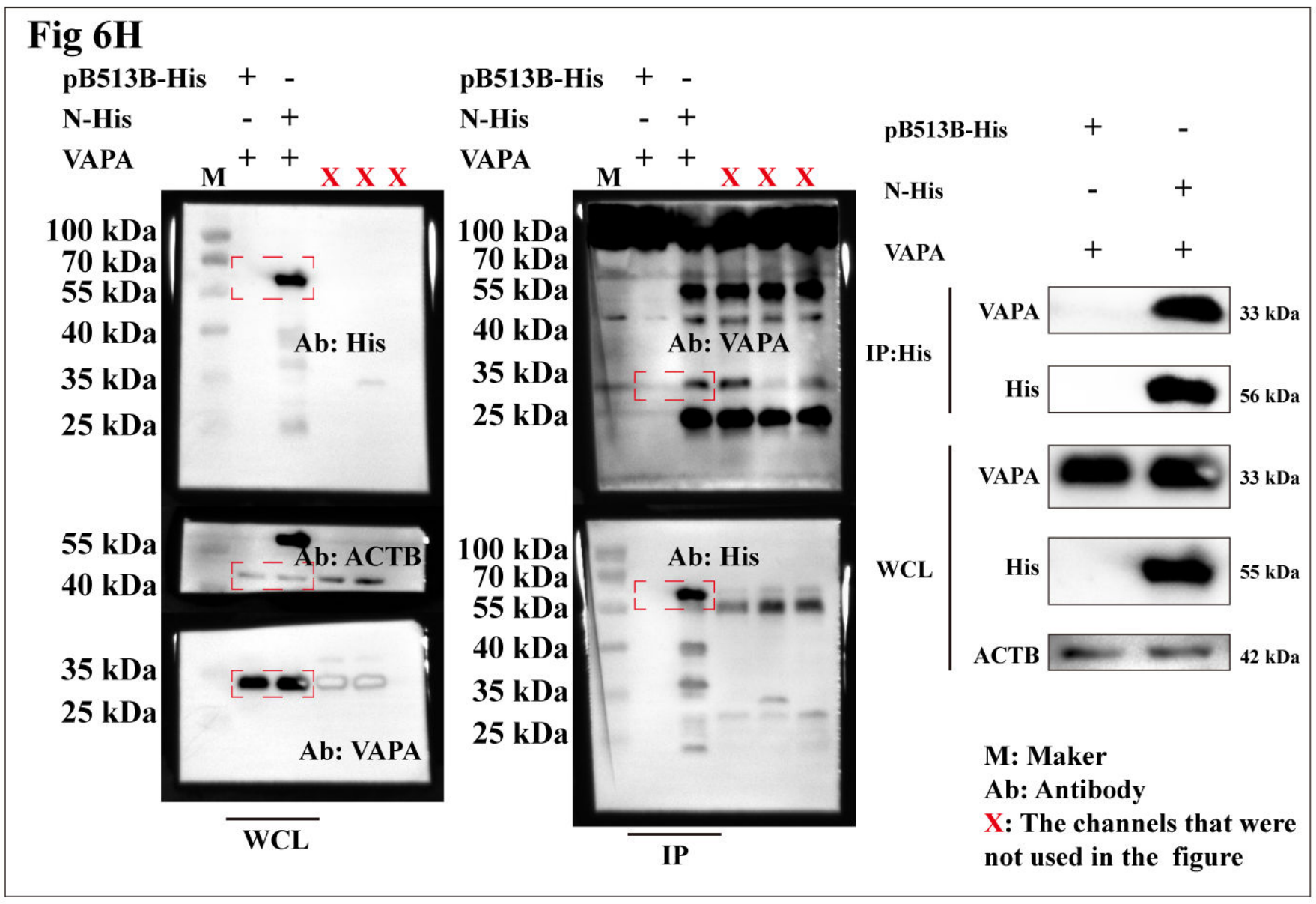
Fig 6H
pB513B-His + -
N-His - +
VAPA + +
M X X X
100 kDa
70 kDa
55 kDa
40 kDa
35 kDa
25 kDa
Ab: His
Ab: ACTB
Ab: VAPA
WCL
Ab: VAPA
Ab: His
IP
pB513B-His + -
N-His - +
VAPA + +
IP:His
VAPA 33 kDa
His 56 kDa
WCL
VAPA 33 kDa
His 55 kDa
ACTB 42 kDa
M: Maker
Ab: Antibody
X: The channels that were not used in the figure

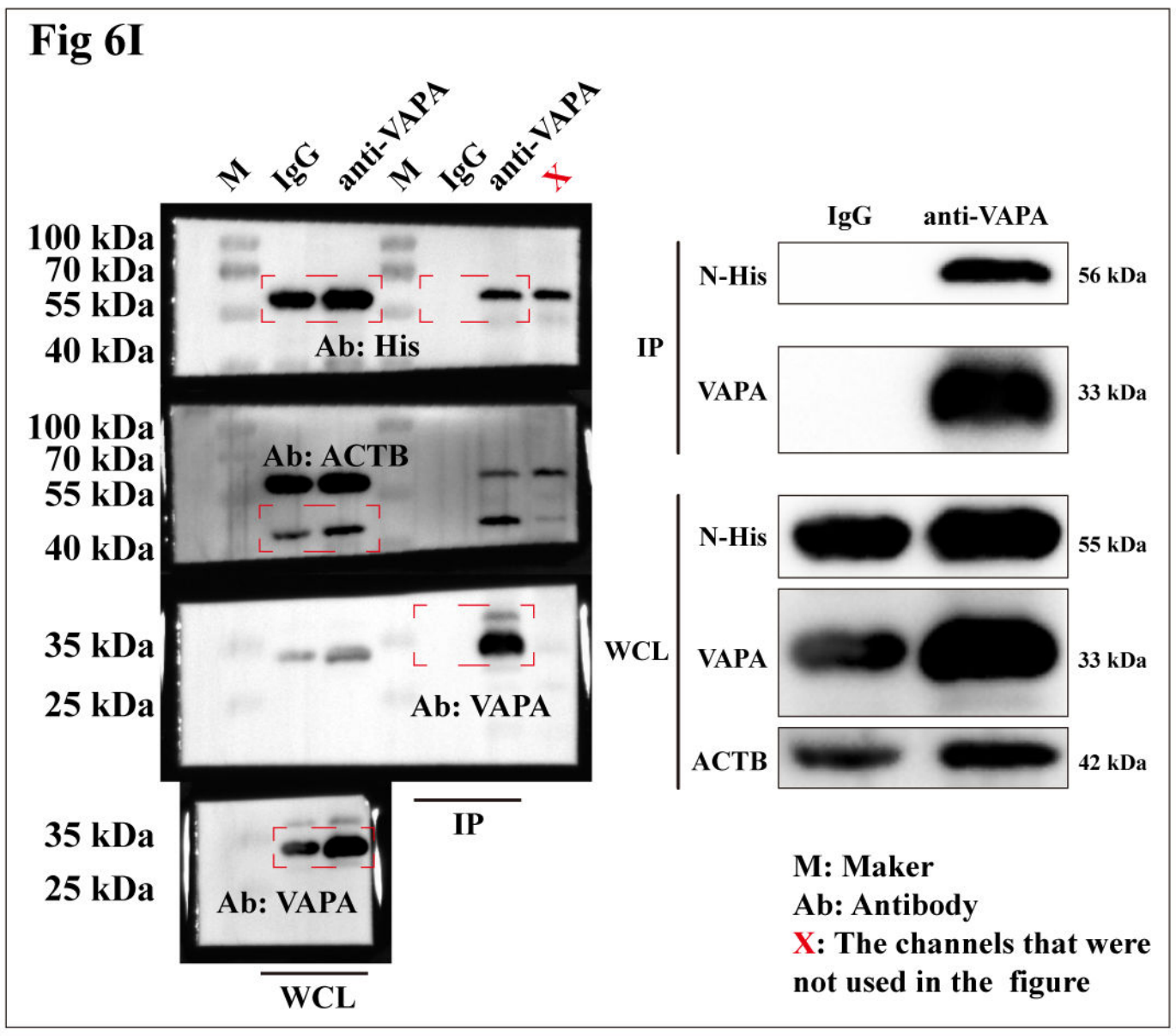
Fig 6I
M IgG anti-VAPA M IgG anti-VAPA X
100 kDa
70 kDa
55 kDa
40 kDa
35 kDa
25 kDa
Ab: His
Ab: ACTB
Ab: VAPA
IP
Ab: VAPA
WCL
IgG anti-VAPA
IP
N-His 56 kDa
VAPA 33 kDa
WCL
N-His 55 kDa
VAPA 33 kDa
ACTB 42 kDa
M: Maker
Ab: Antibody
X: The channels that were not used in the figure

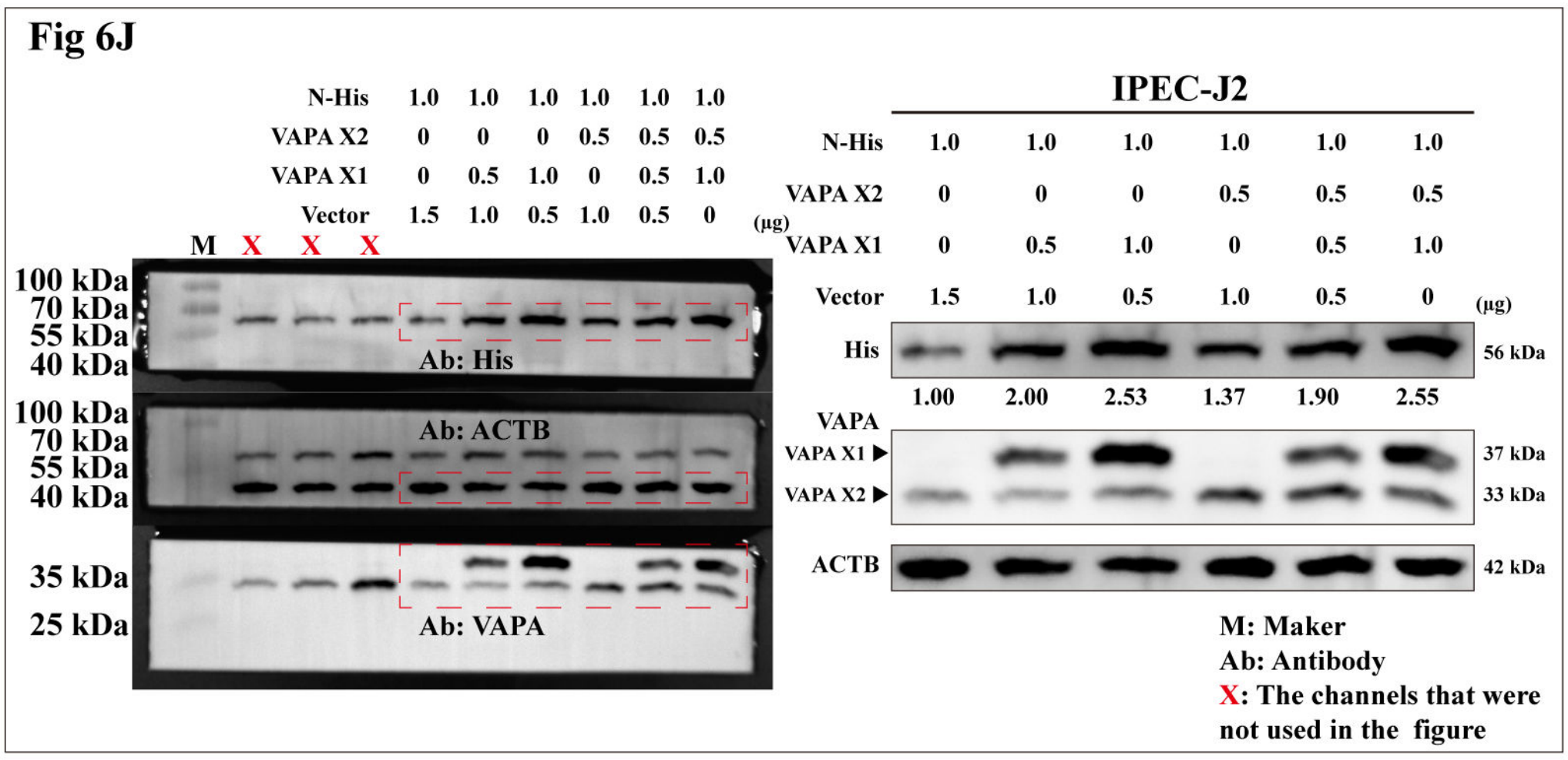
Fig 6J
N-His 1.0 1.0 1.0 1.0 1.0 1.0
VAPA X2 0 0 0 0.5 0.5 0.5
VAPA X1 0 0.5 1.0 0 0.5 1.0
Vector 1.5 1.0 0.5 1.0 0.5 0 (μg)
M X X X
Ab: His
Ab: ACTB
Ab: VAPA
IPEC-J2
N-His 1.0 1.0 1.0 1.0 1.0 1.0
VAPA X2 0 0 0 0.5 0.5 0.5
VAPA X1 0 0.5 1.0 0 0.5 1.0
Vector 1.5 1.0 0.5 1.0 0.5 0 (μg)
His 56 kDa
1.00 2.00 2.53 1.37 1.90 2.55
VAPA
VAPA X1 37 kDa
VAPA X2 33 kDa
ACTB 42 kDa
M: Maker
Ab: Antibody
X: The channels that were not used in the figure

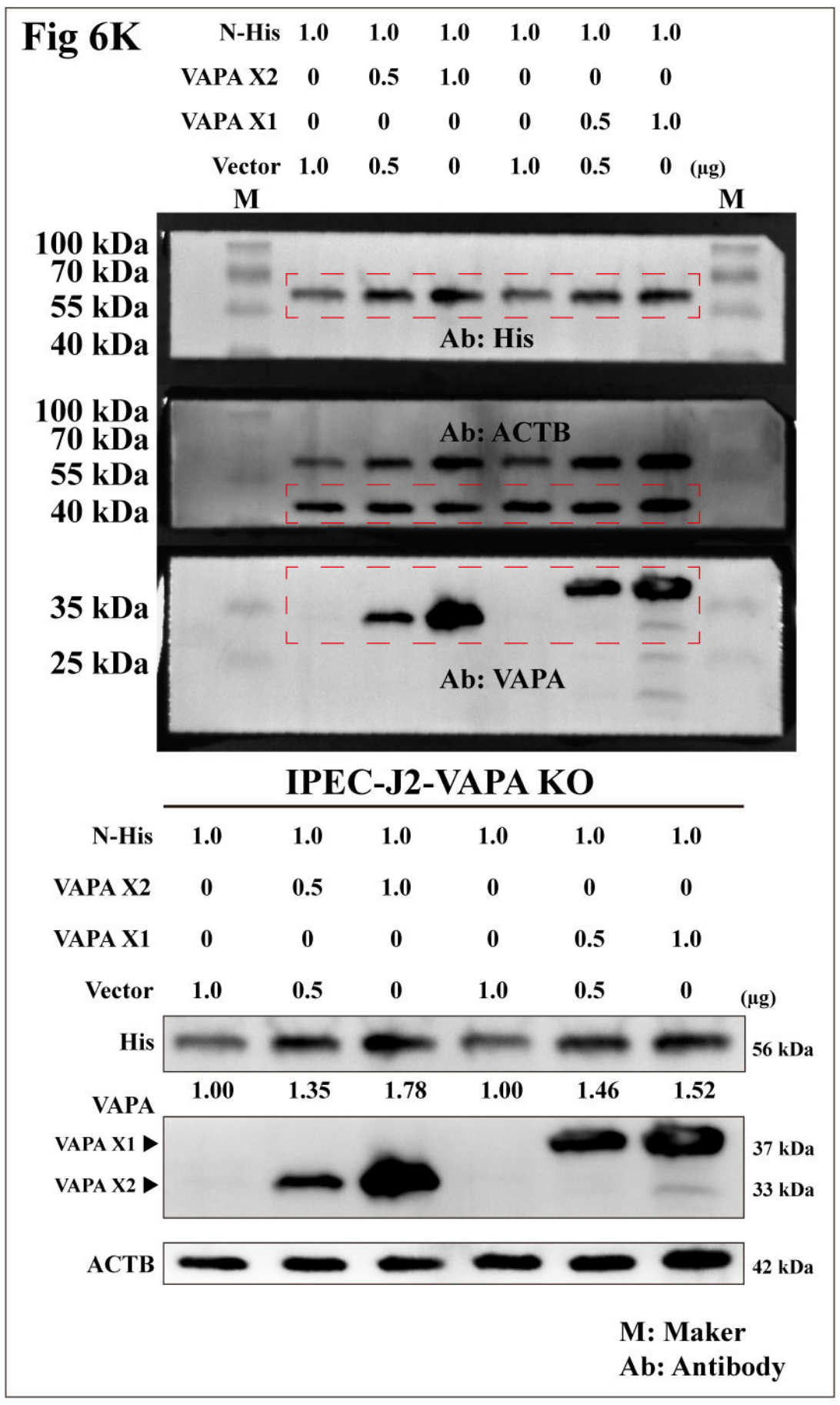
Fig 6K
N-His 1.0 1.0 1.0 1.0 1.0 1.0
VAPA X2 0 0.5 1.0 0 0 0
VAPA X1 0 0 0 0 0.5 1.0
Vector 1.0 0.5 0 1.0 0.5 0 (μg)
M M
Ab: His
Ab: ACTB
Ab: VAPA
IPEC-J2-VAPA KO
N-His 1.0 1.0 1.0 1.0 1.0 1.0
VAPA X2 0 0.5 1.0 0 0 0
VAPA X1 0 0 0 0 0.5 1.0
Vector 1.0 0.5 0 1.0 0.5 0 (μg)
His 56 kDa
1.00 1.35 1.78 1.00 1.46 1.52
VAPA
VAPA X1 37 kDa
VAPA X2 33 kDa
ACTB 42 kDa
M: Maker
Ab: Antibody

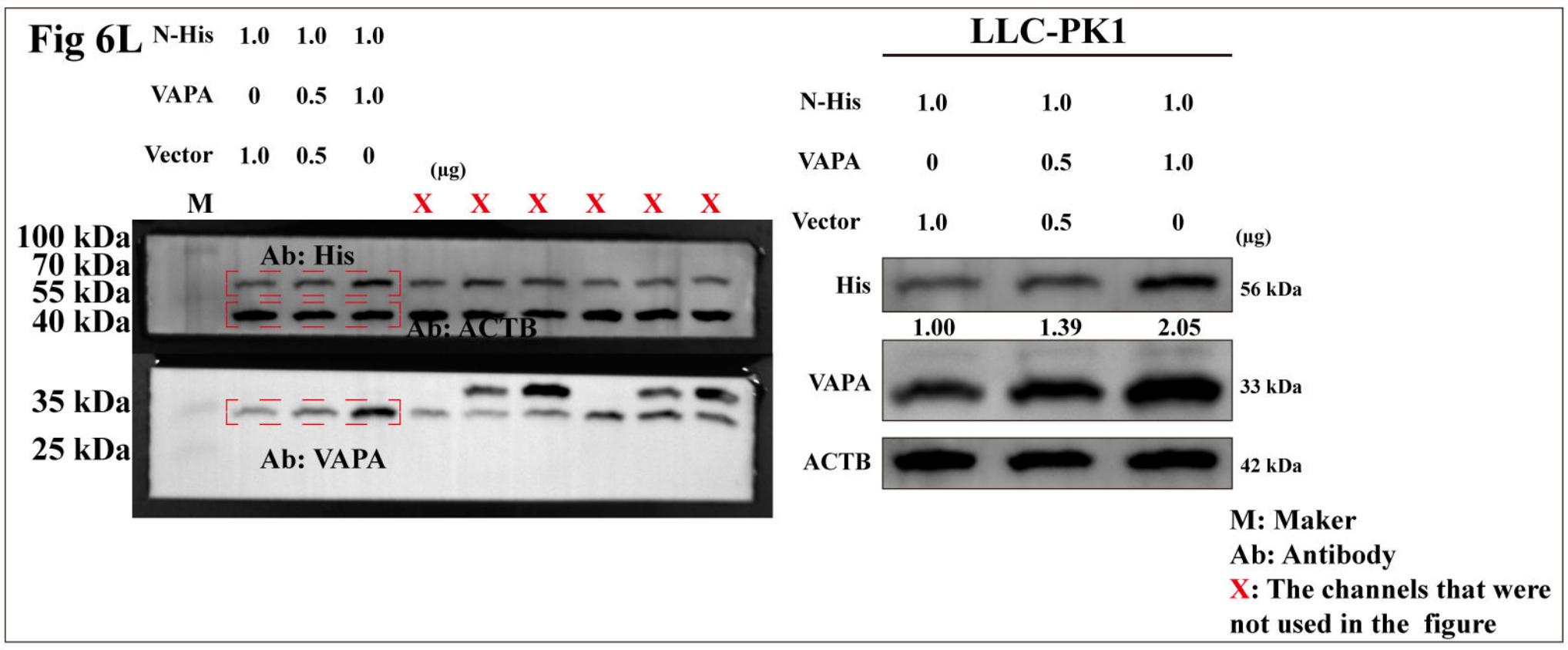
Fig 6L
N-His 1.0 1.0 1.0
VAPA 0 0.5 1.0
Vector 1.0 0.5 0 (μg)
M X X X X X X
Ab: His
Ab: ACTB
Ab: VAPA
LLC-PK1
N-His 1.0 1.0 1.0
VAPA 0 0.5 1.0
Vector 1.0 0.5 0 (μg)
His 56 kDa
1.00 1.39 2.05
VAPA 33 kDa
ACTB 42 kDa
M: Maker
Ab: Antibody
X: The channels that were not used in the figure

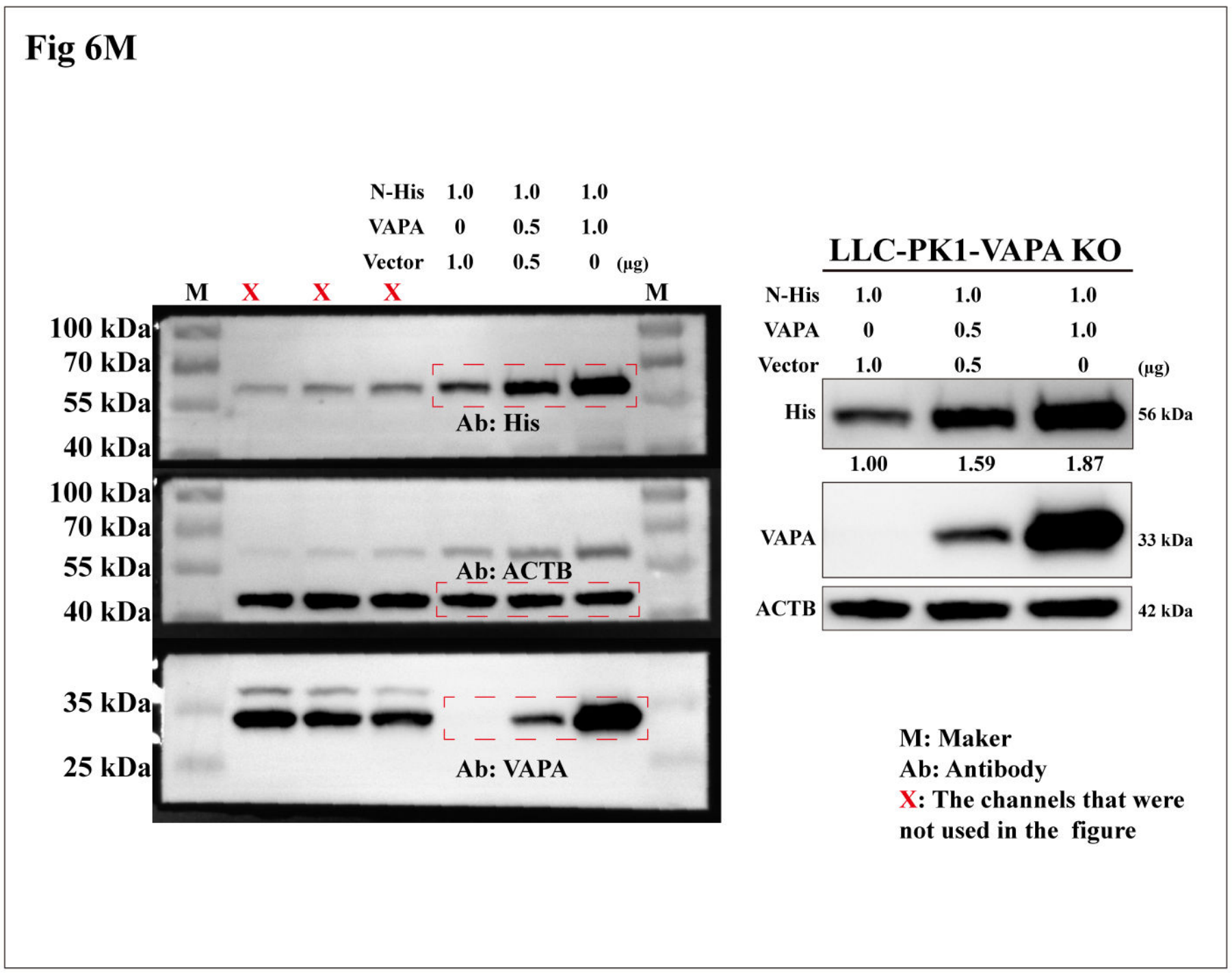
Fig 6M
N-His 1.0 1.0 1.0
VAPA 0 0.5 1.0
Vector 1.0 0.5 0 (μg)
M X X X M
Ab: His
Ab: ACTB
Ab: VAPA
LLC-PK1-VAPA KO
N-His 1.0 1.0 1.0
VAPA 0 0.5 1.0
Vector 1.0 0.5 0 (μg)
His 56 kDa
1.00 1.59 1.87
VAPA 33 kDa
ACTB 42 kDa
M: Maker
Ab: Antibody
X: The channels that were not used in the figure

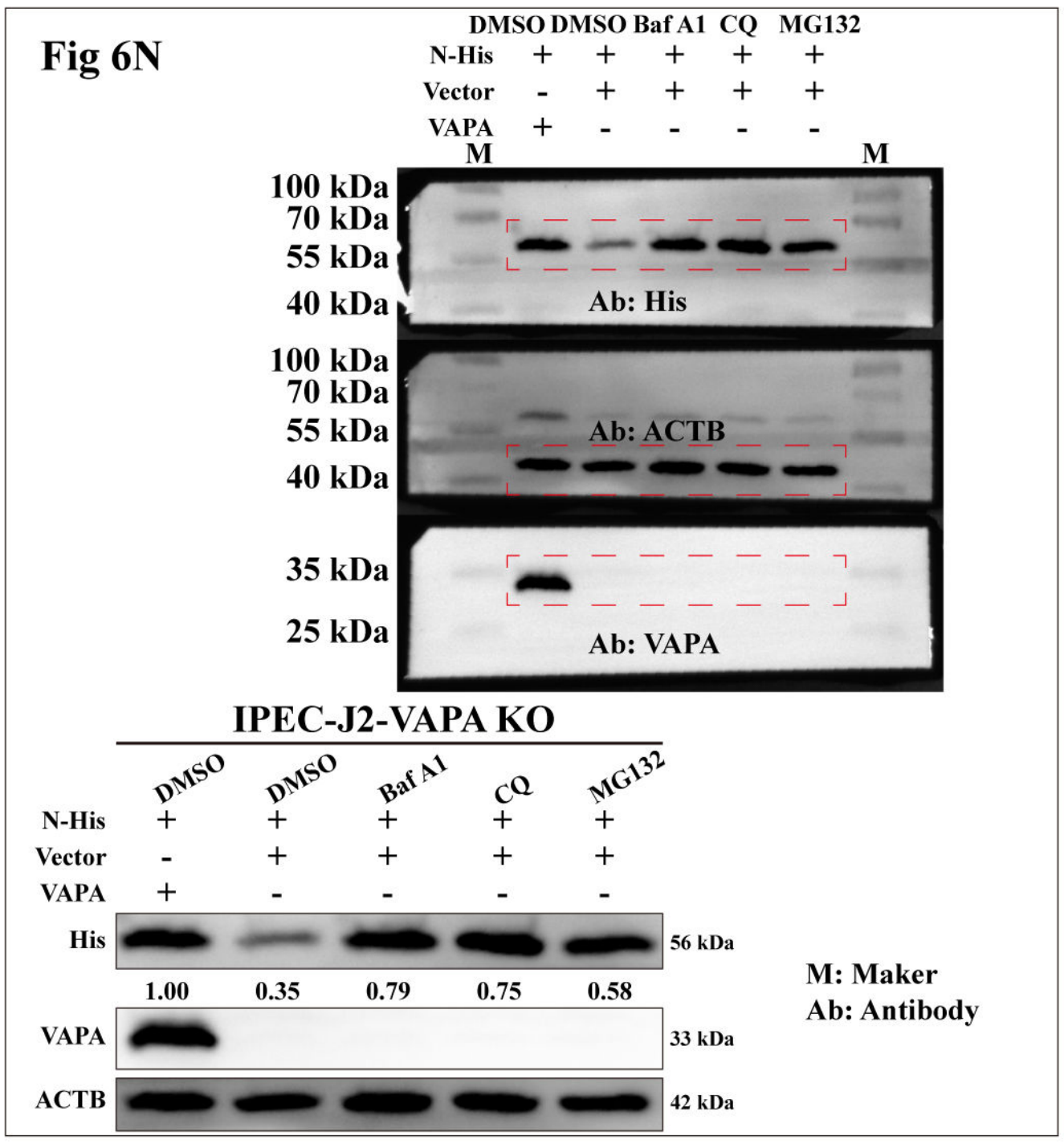
Fig 6N
DMSO DMSO Baf A1 CQ MG132
N-His + + + + +
Vector - + + + +
VAPA + - - - -
M M
Ab: His
Ab: ACTB
Ab: VAPA
IPEC-J2-VAPA KO
DMSO DMSO Baf A1 CQ MG132
N-His + + + + +
Vector - + + + +
VAPA + - - - -
His 56 kDa
1.00 0.35 0.79 0.75 0.58
VAPA 33 kDa
ACTB 42 kDa
M: Maker
Ab: Antibody

Fig 6O

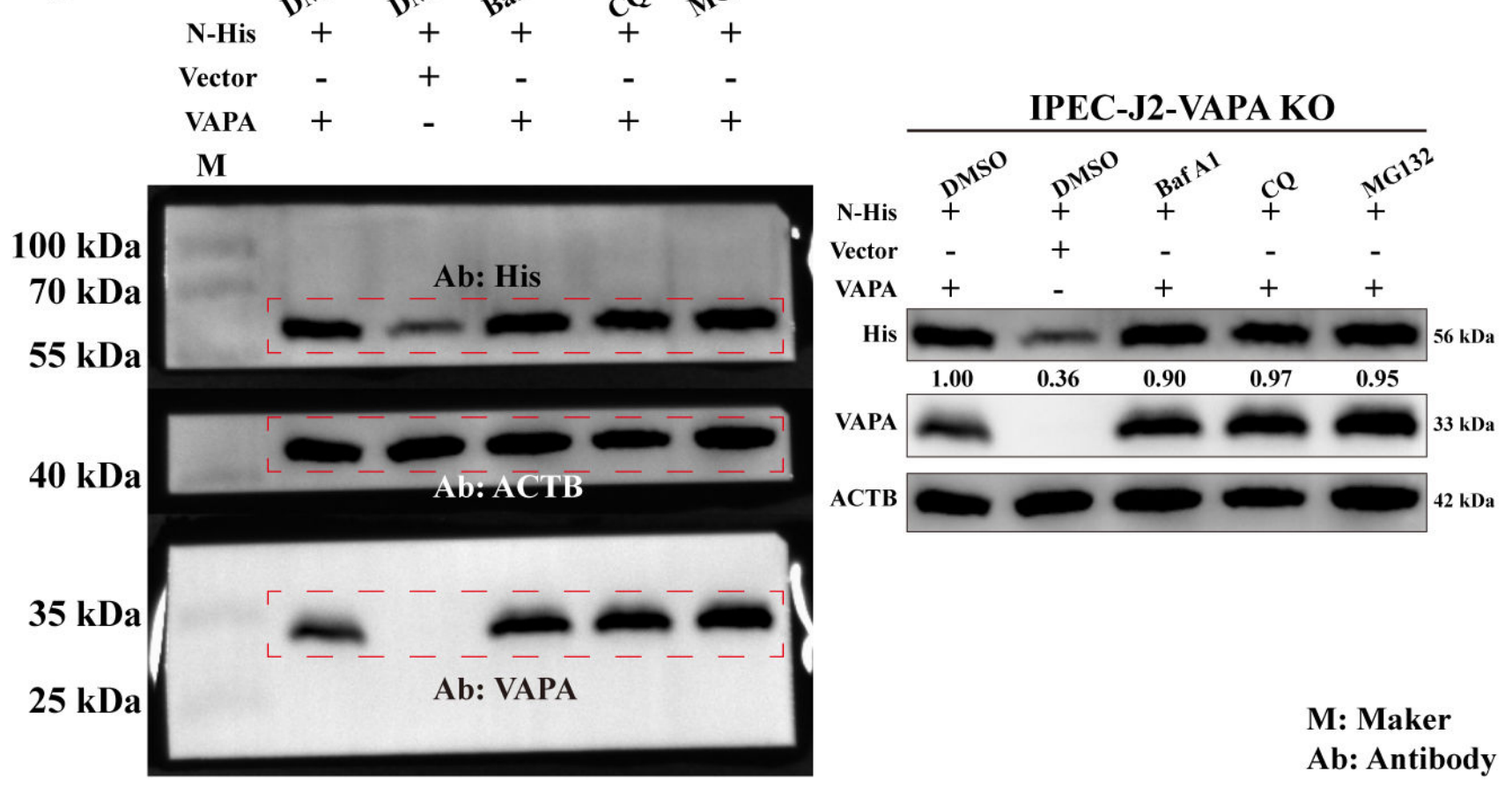

Fig 7A

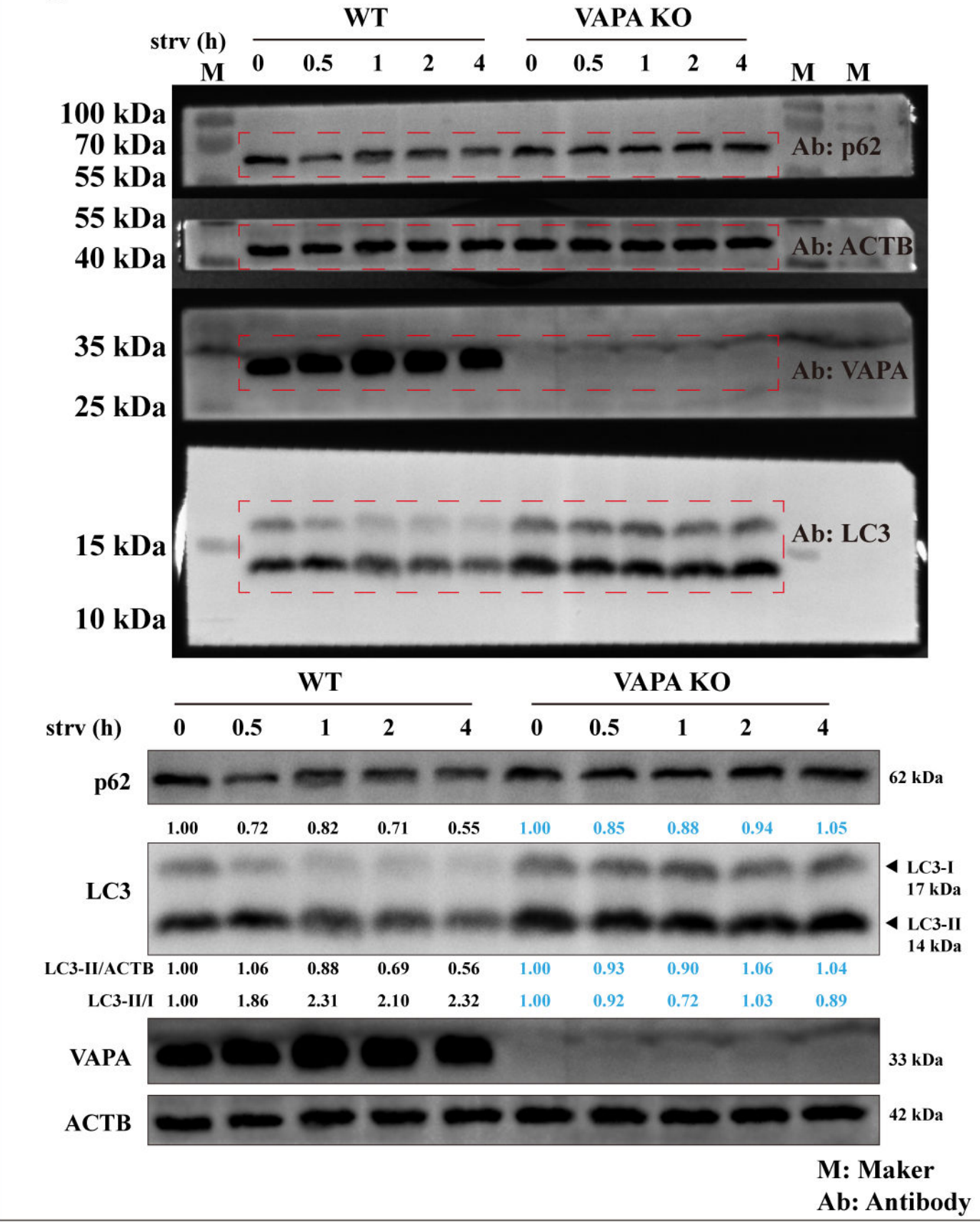

Fig 7B

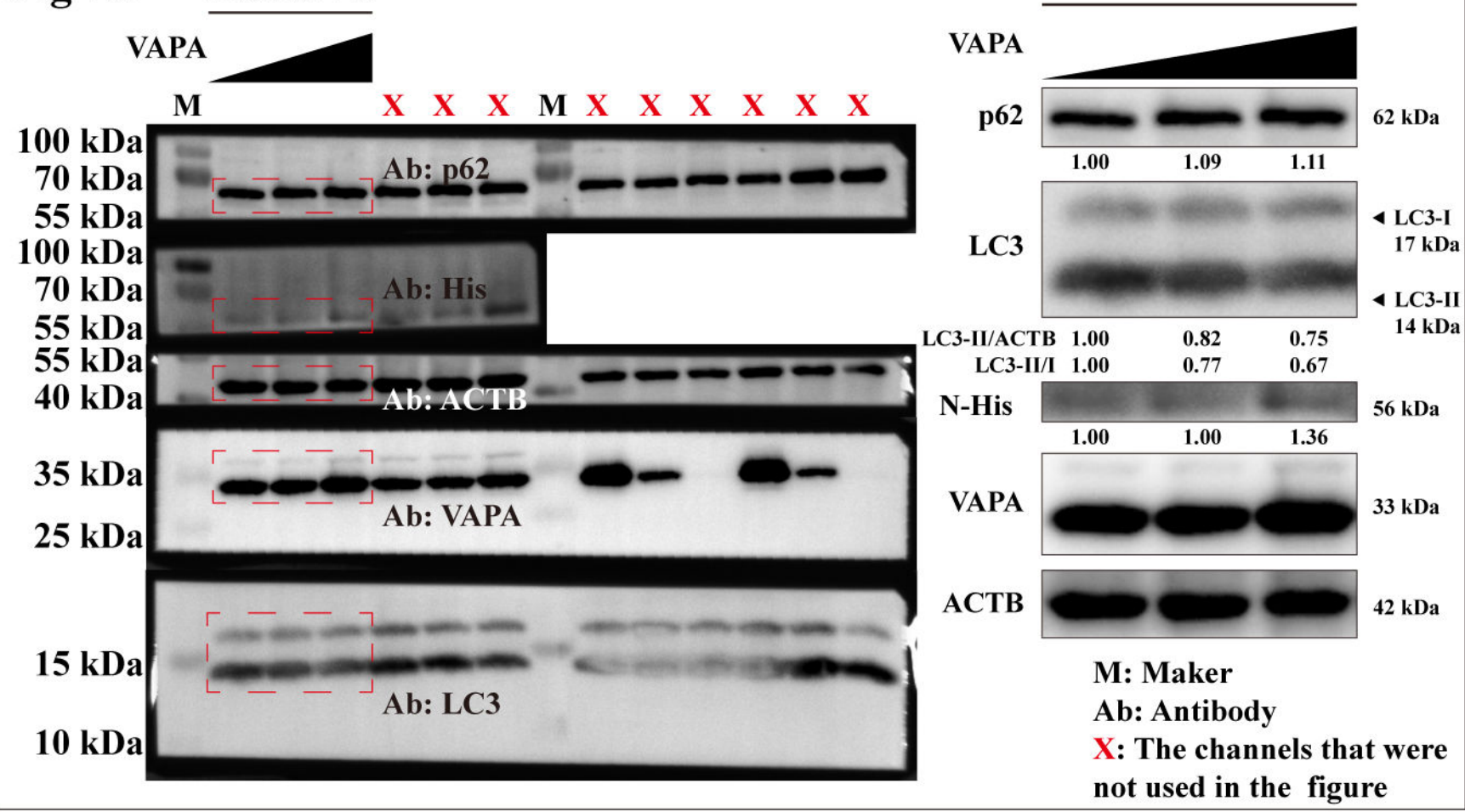

Fig 7D

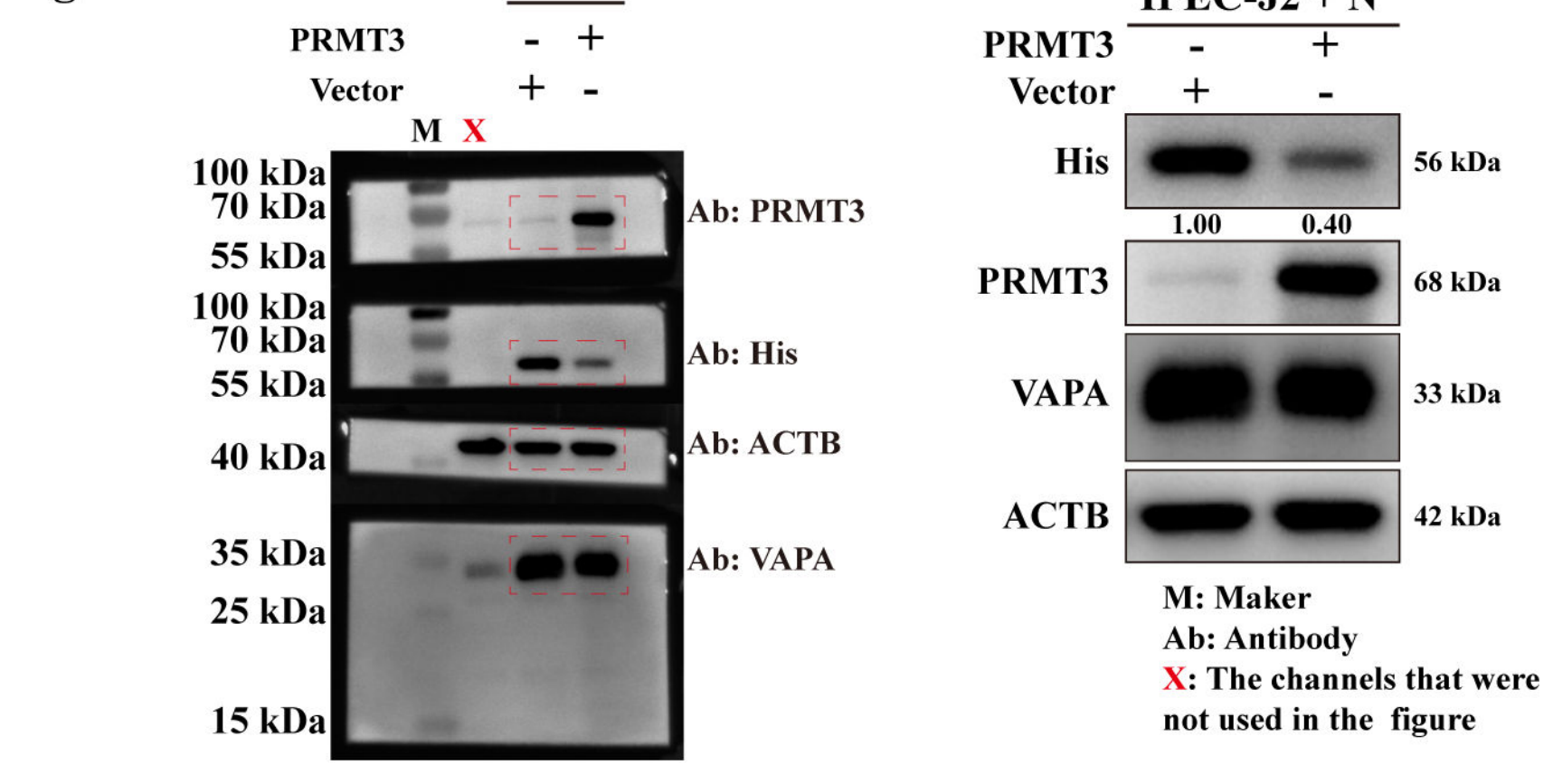

Fig 7E

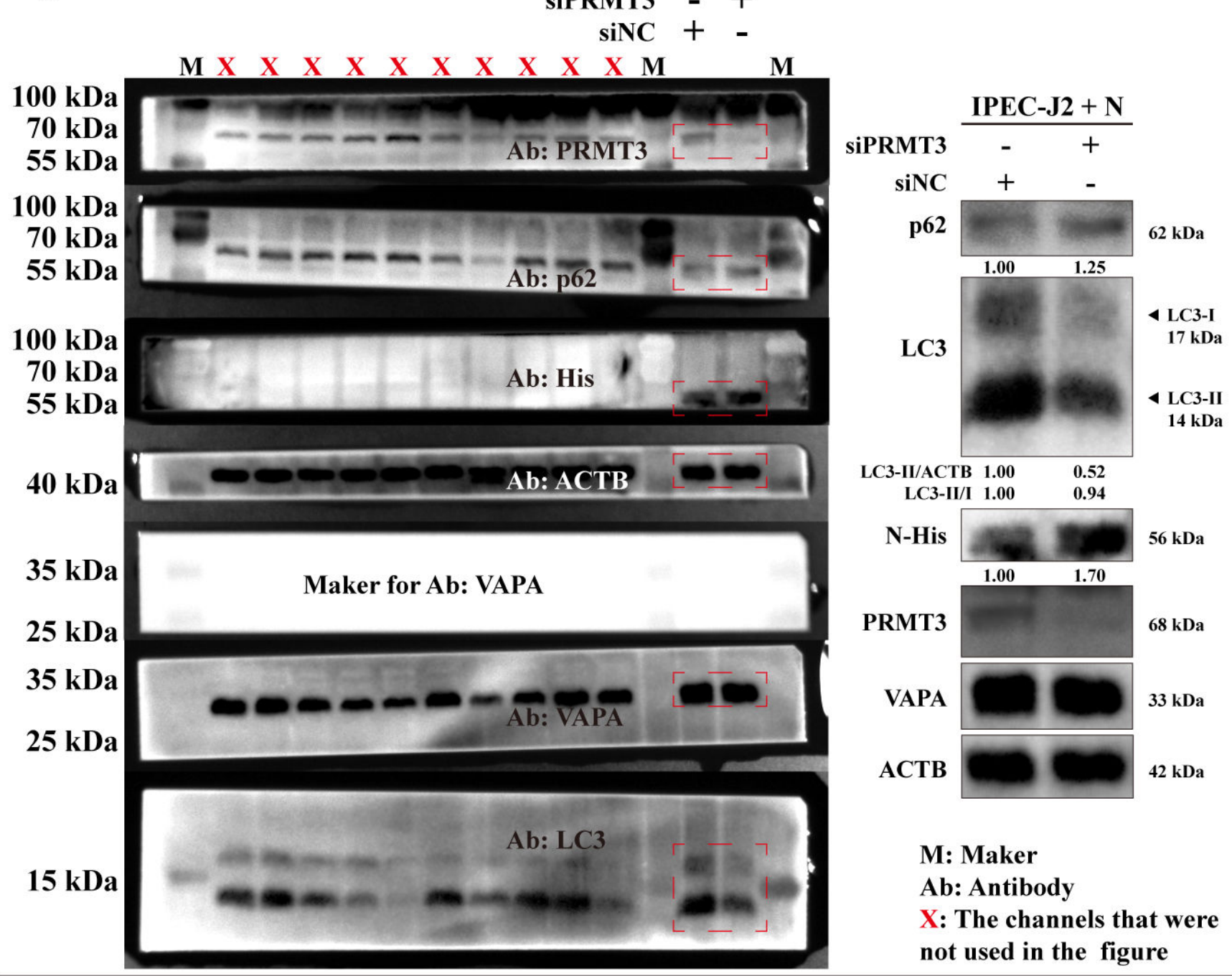

Fig 7F

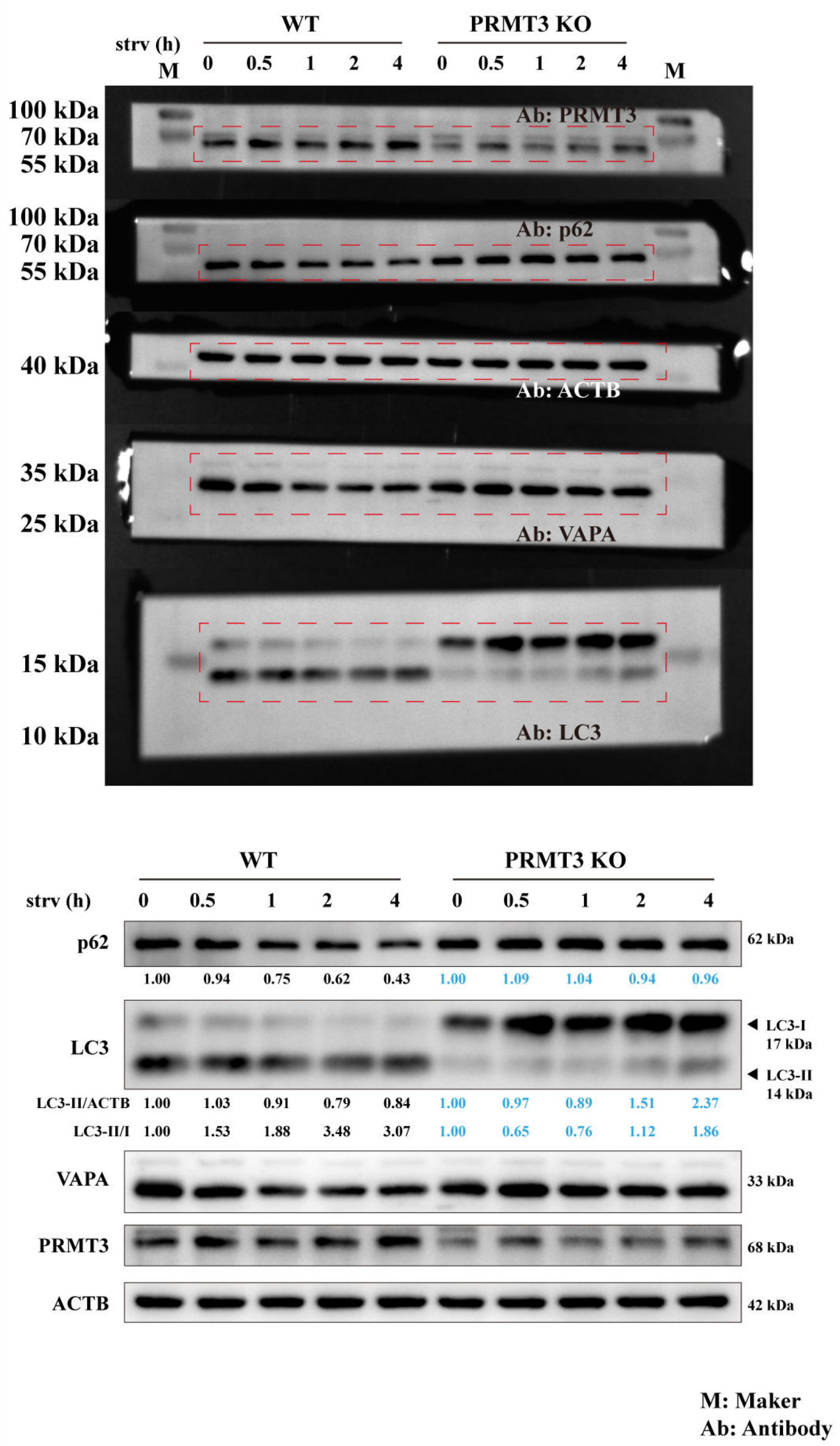

Fig 7G

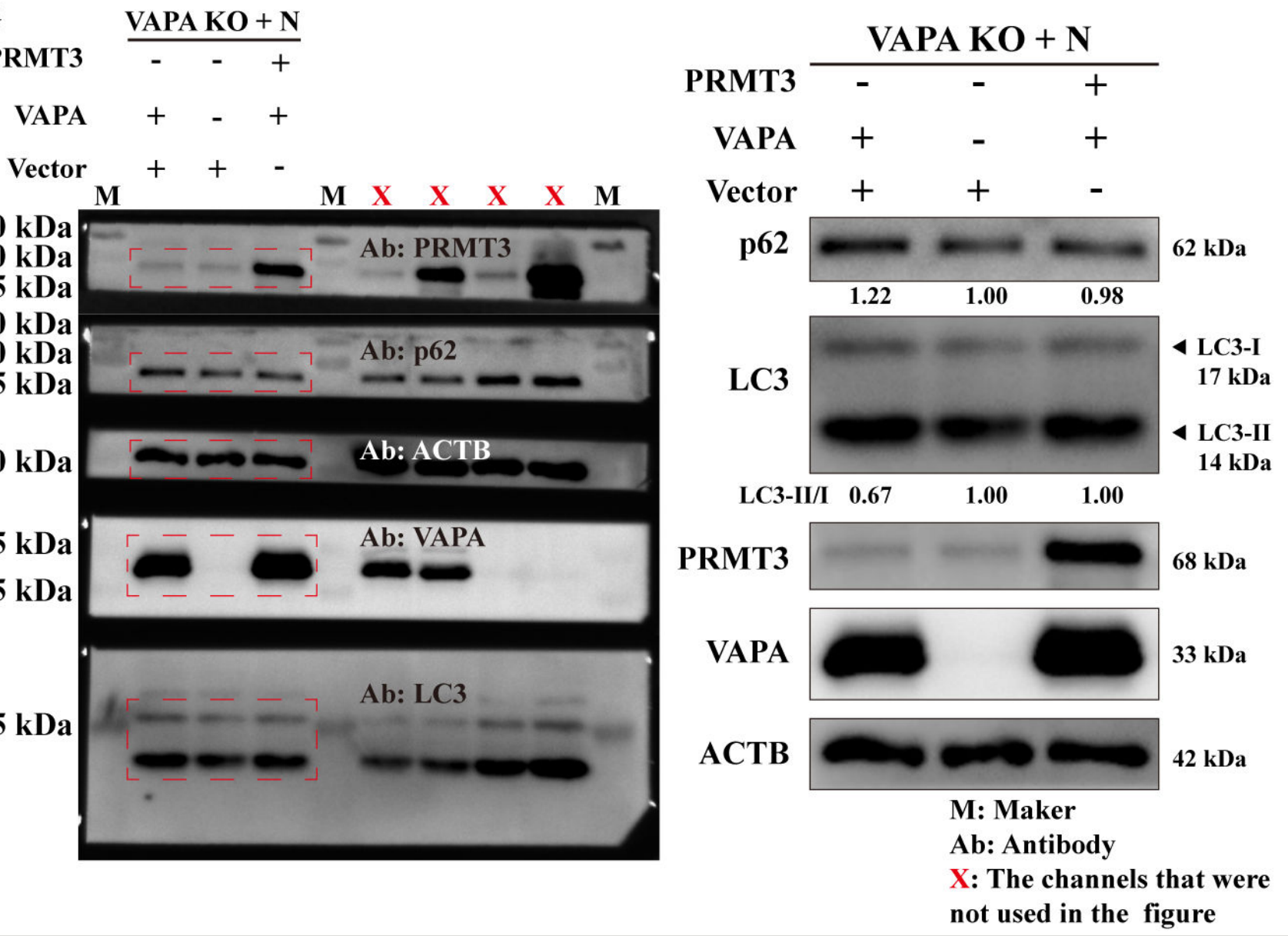

Fig 7H

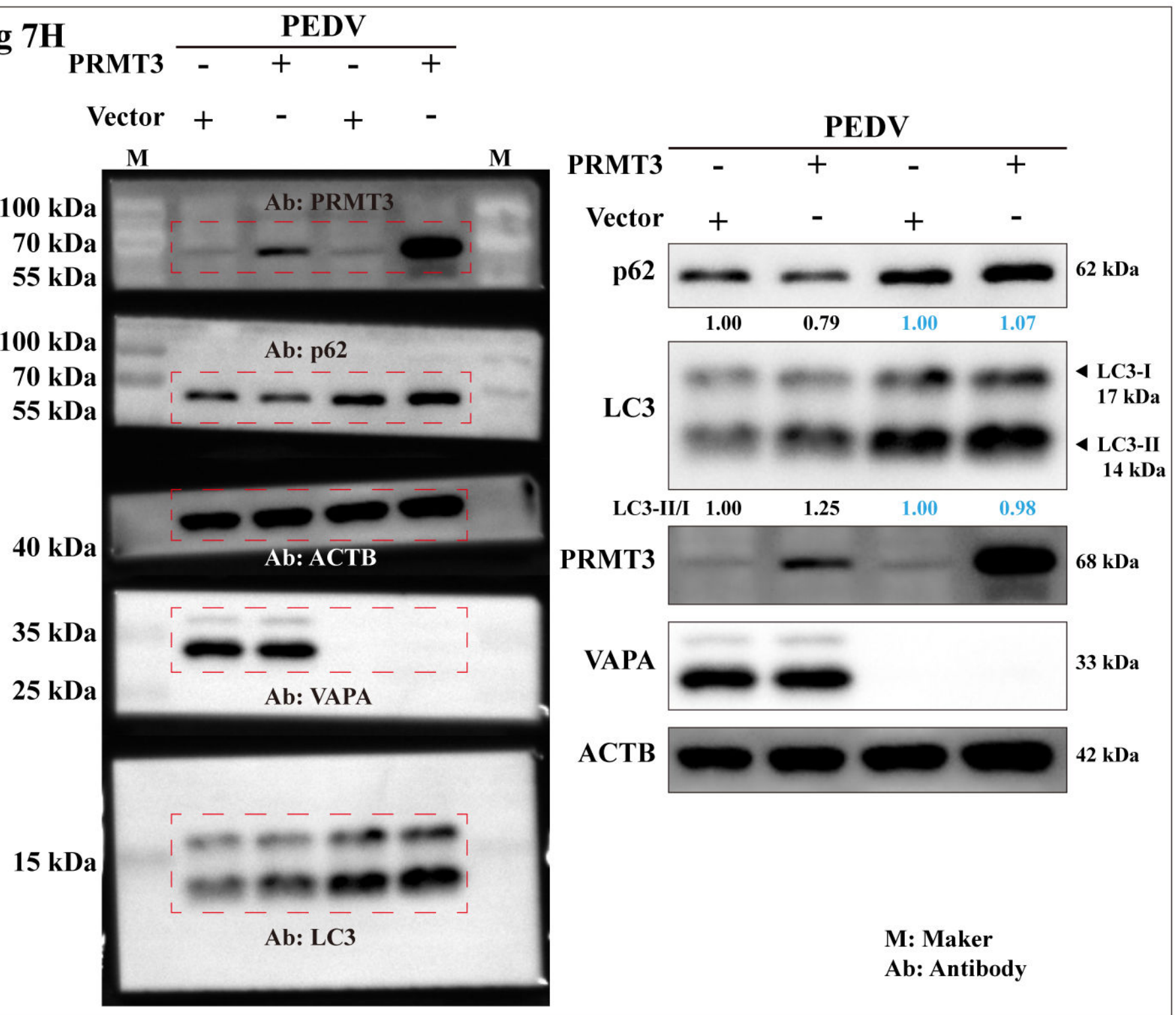

Fig 7I

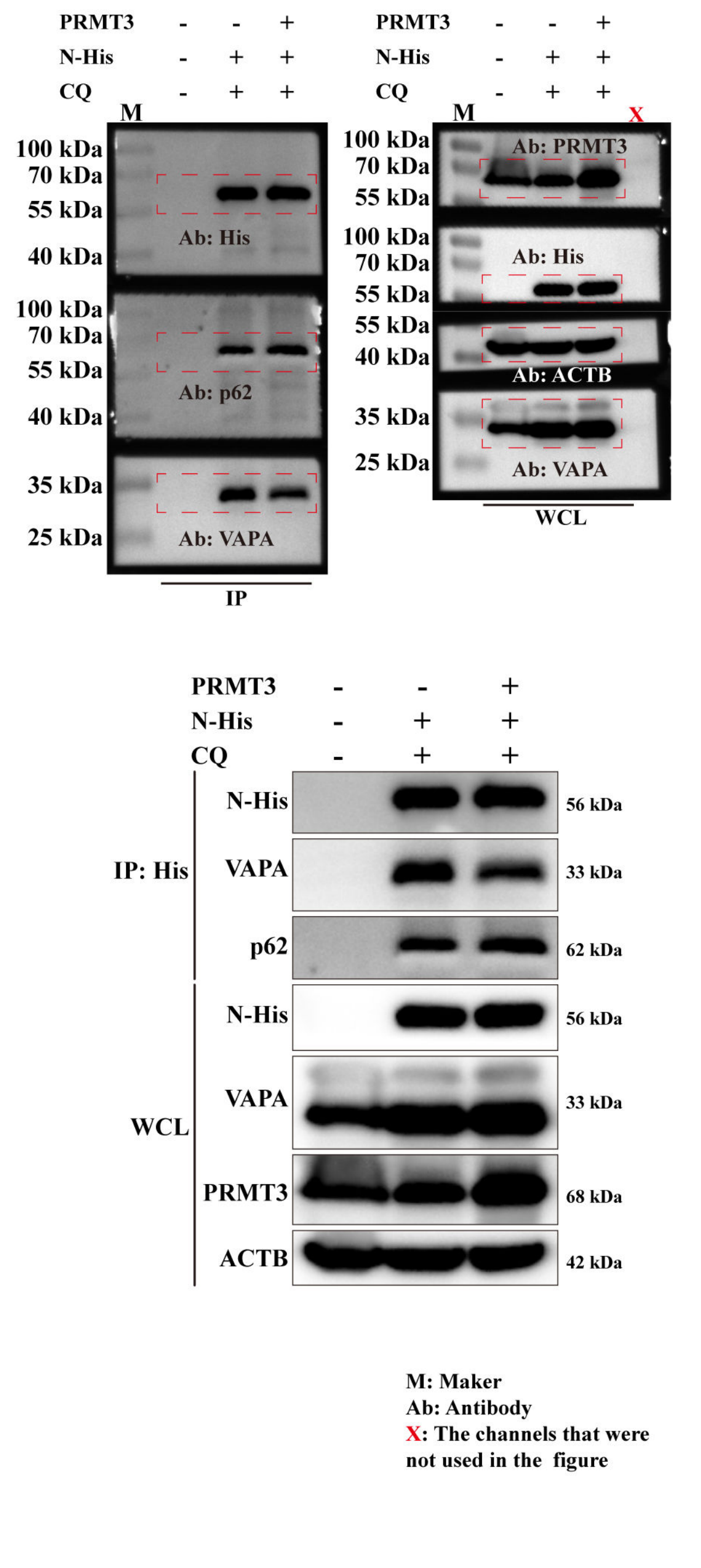

Fig 8F

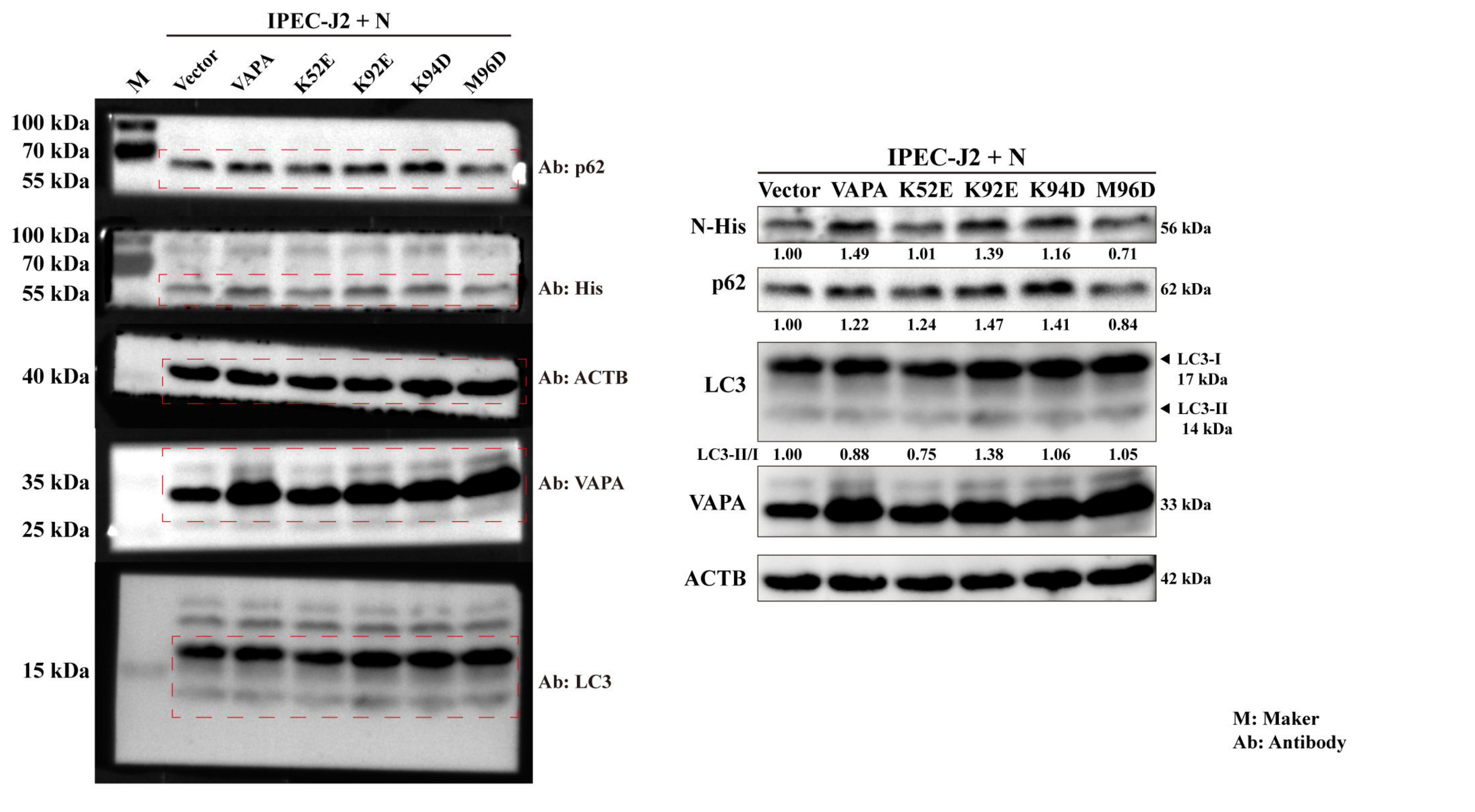

Fig 8G

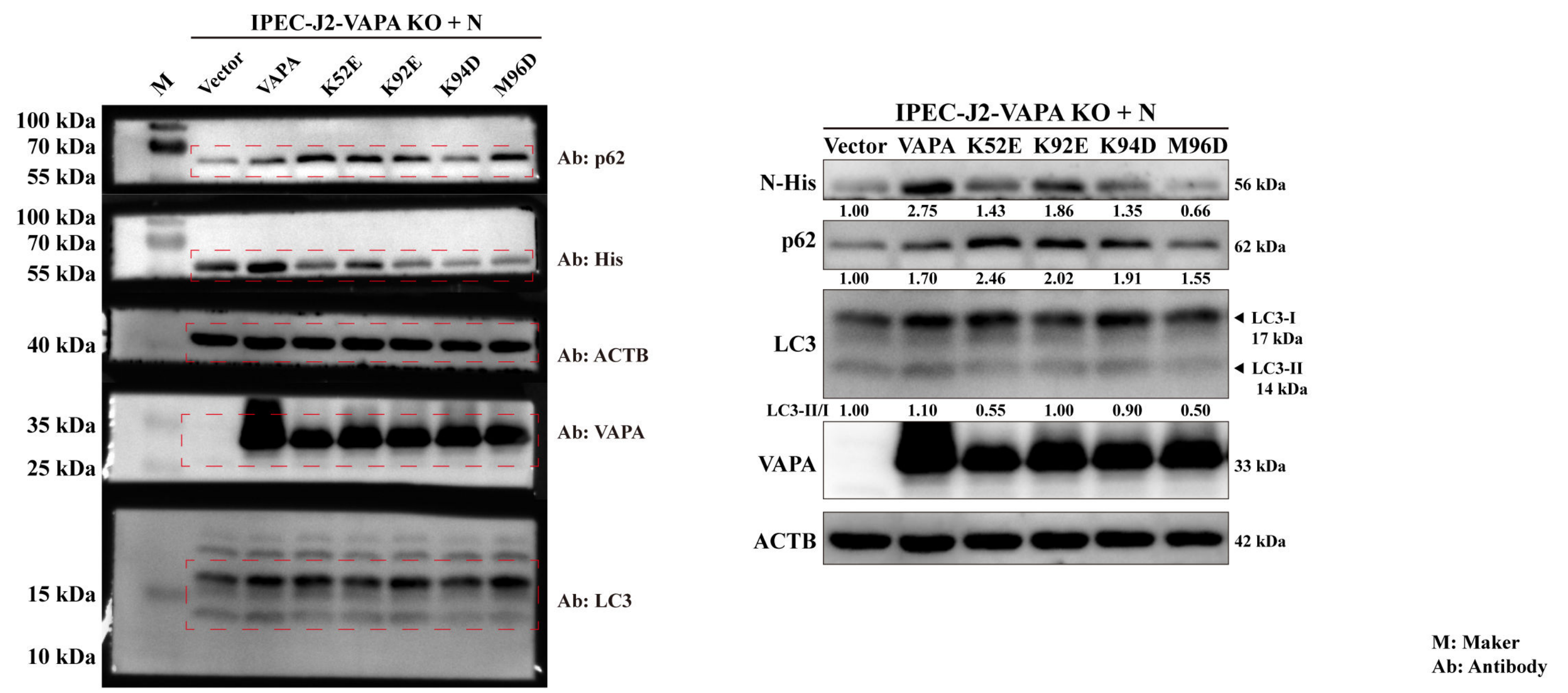

Fig 8I

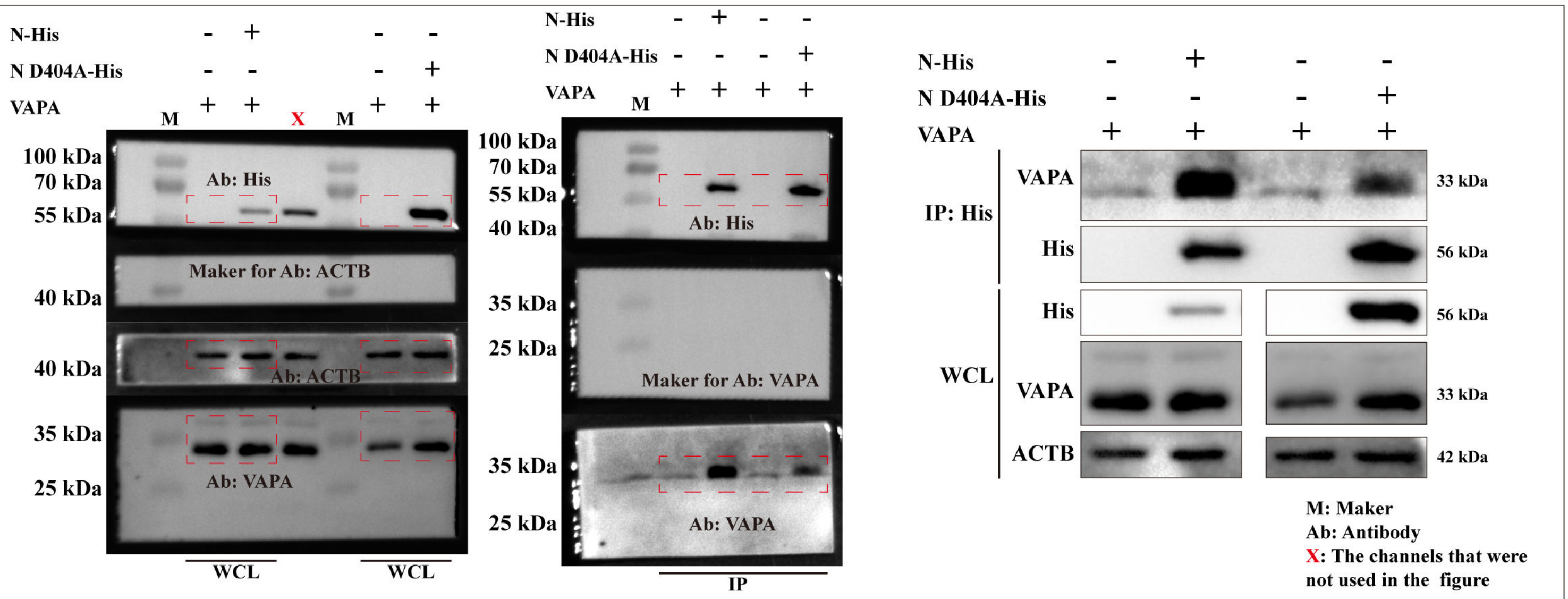

Fig 8J

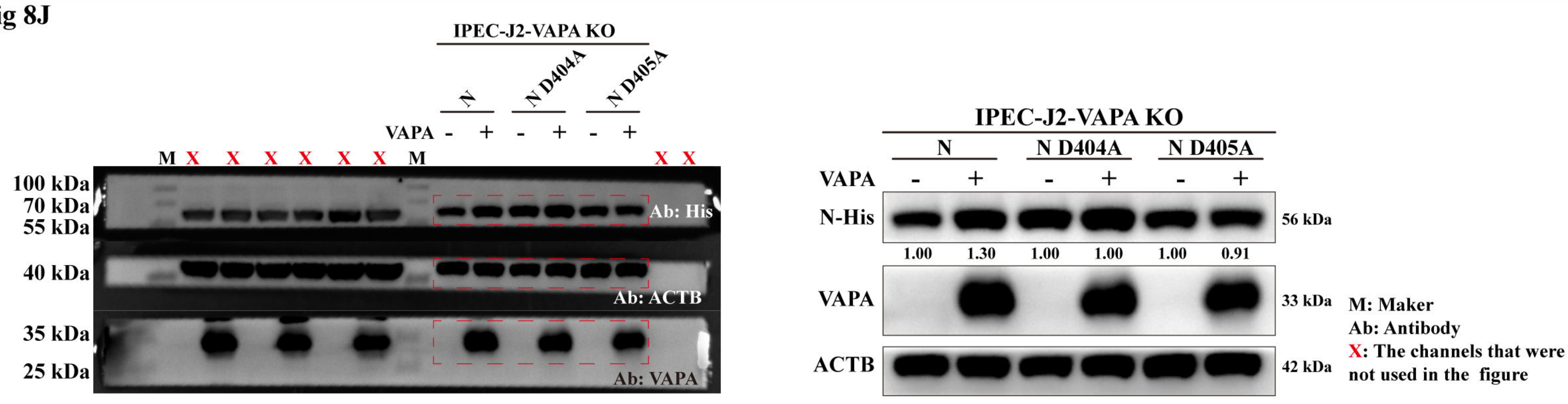

Fig 8K

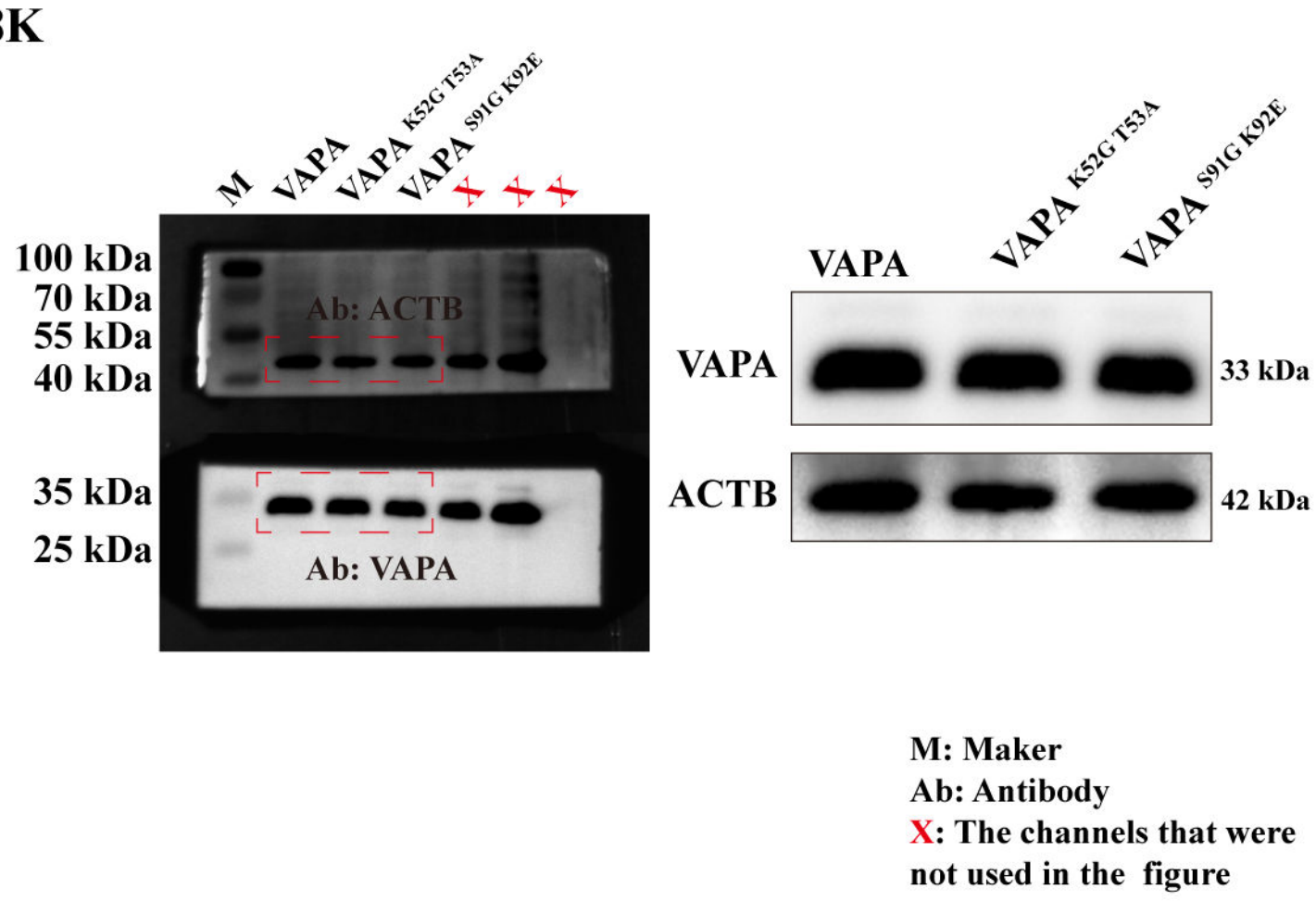

S1H Fig

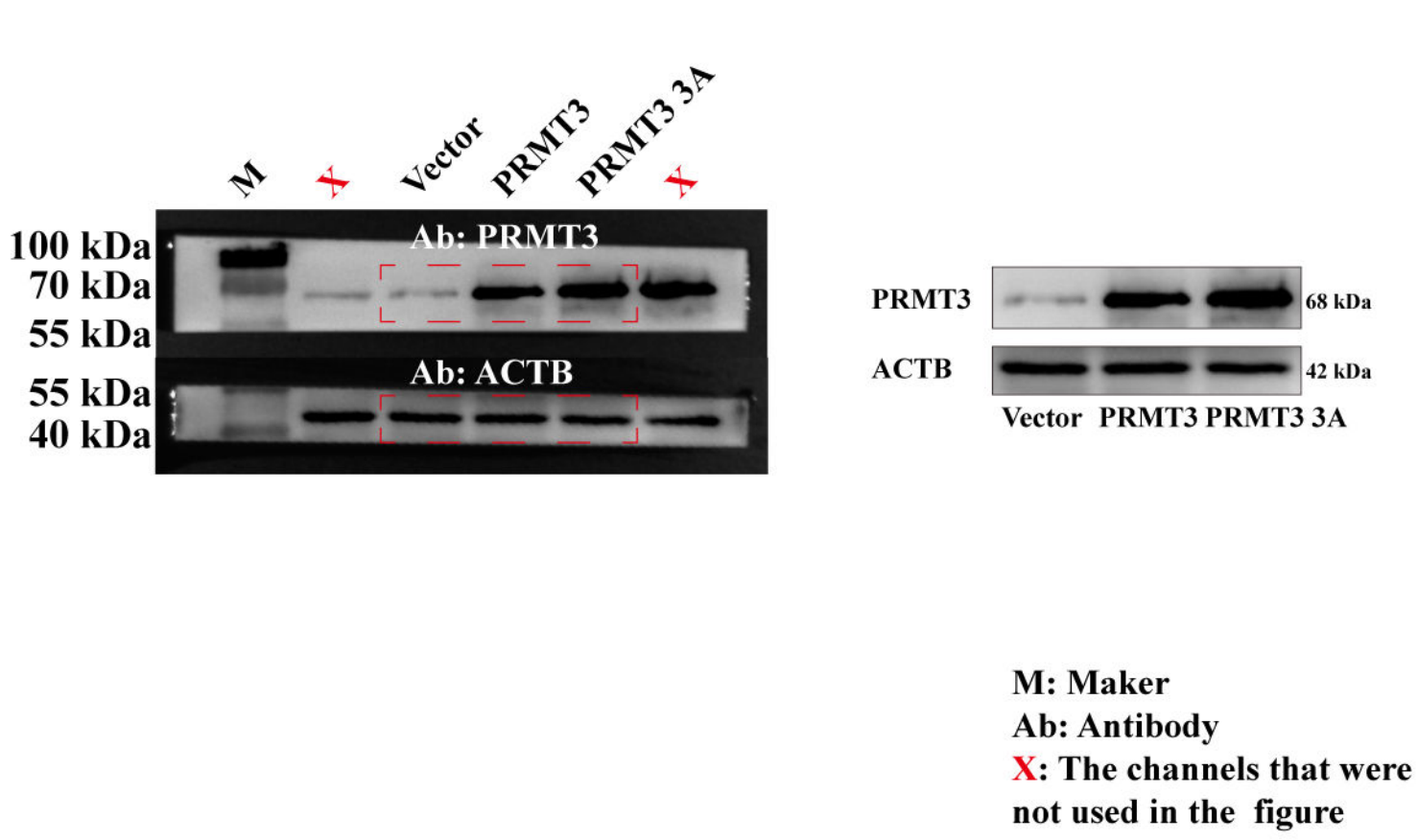

S1J Fig

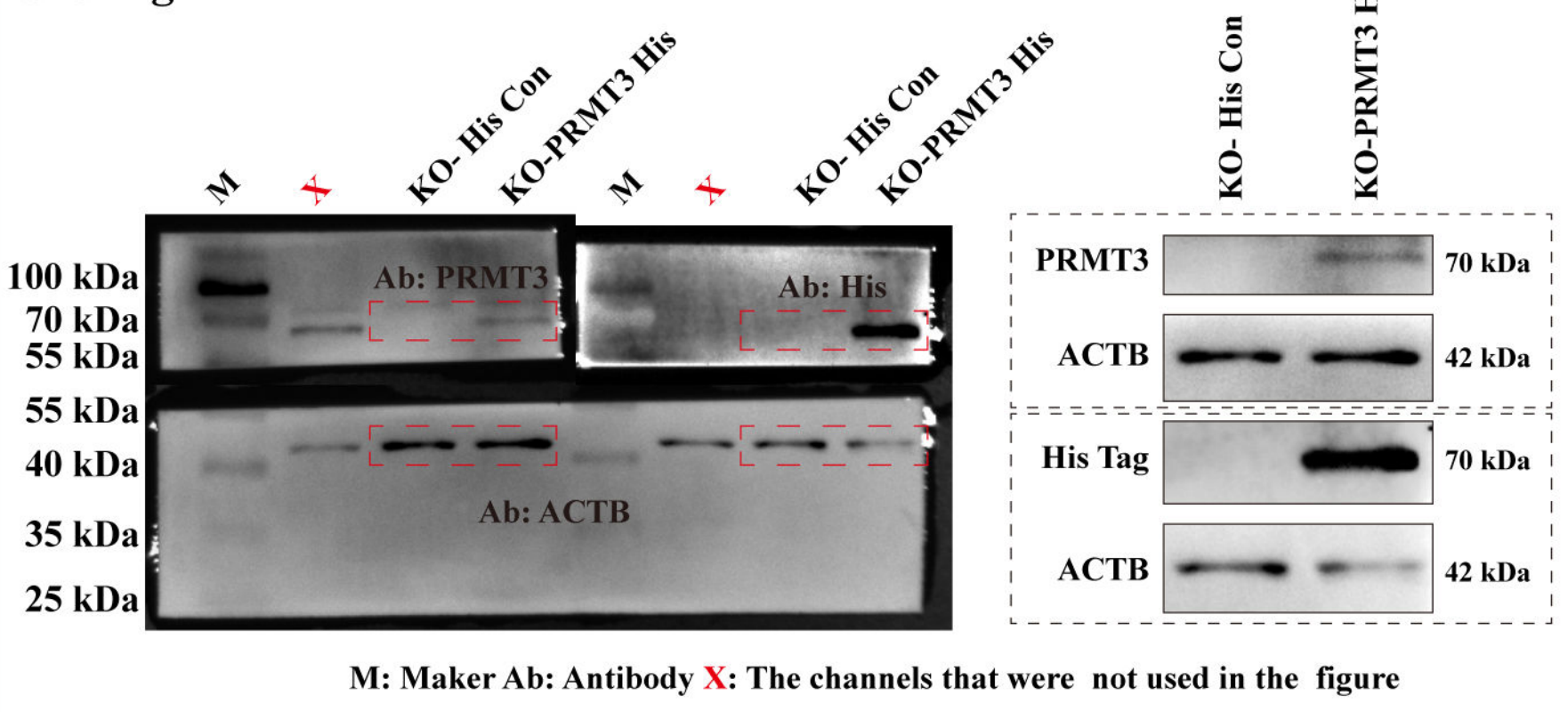

S1K Fig

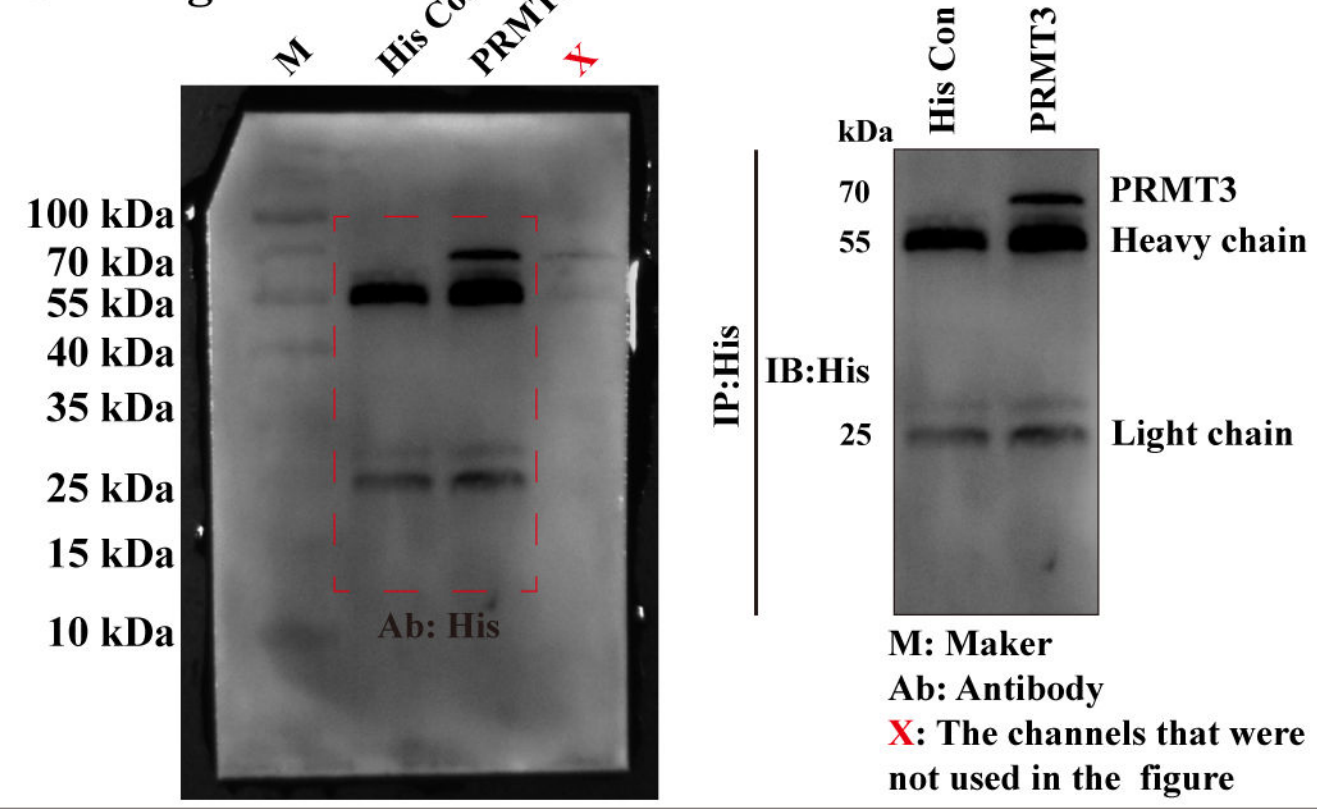

S2B Fig

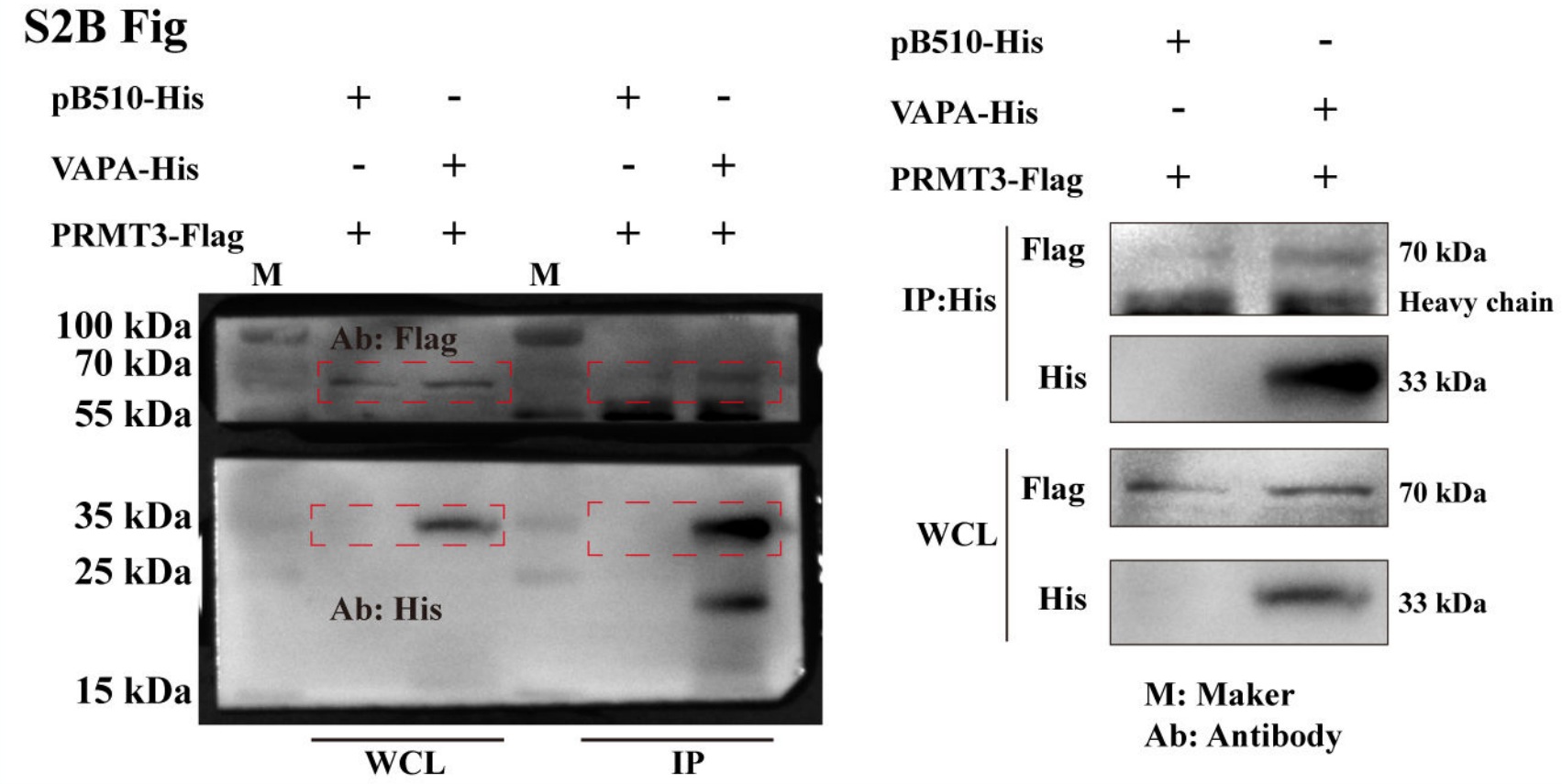

S2F Fig

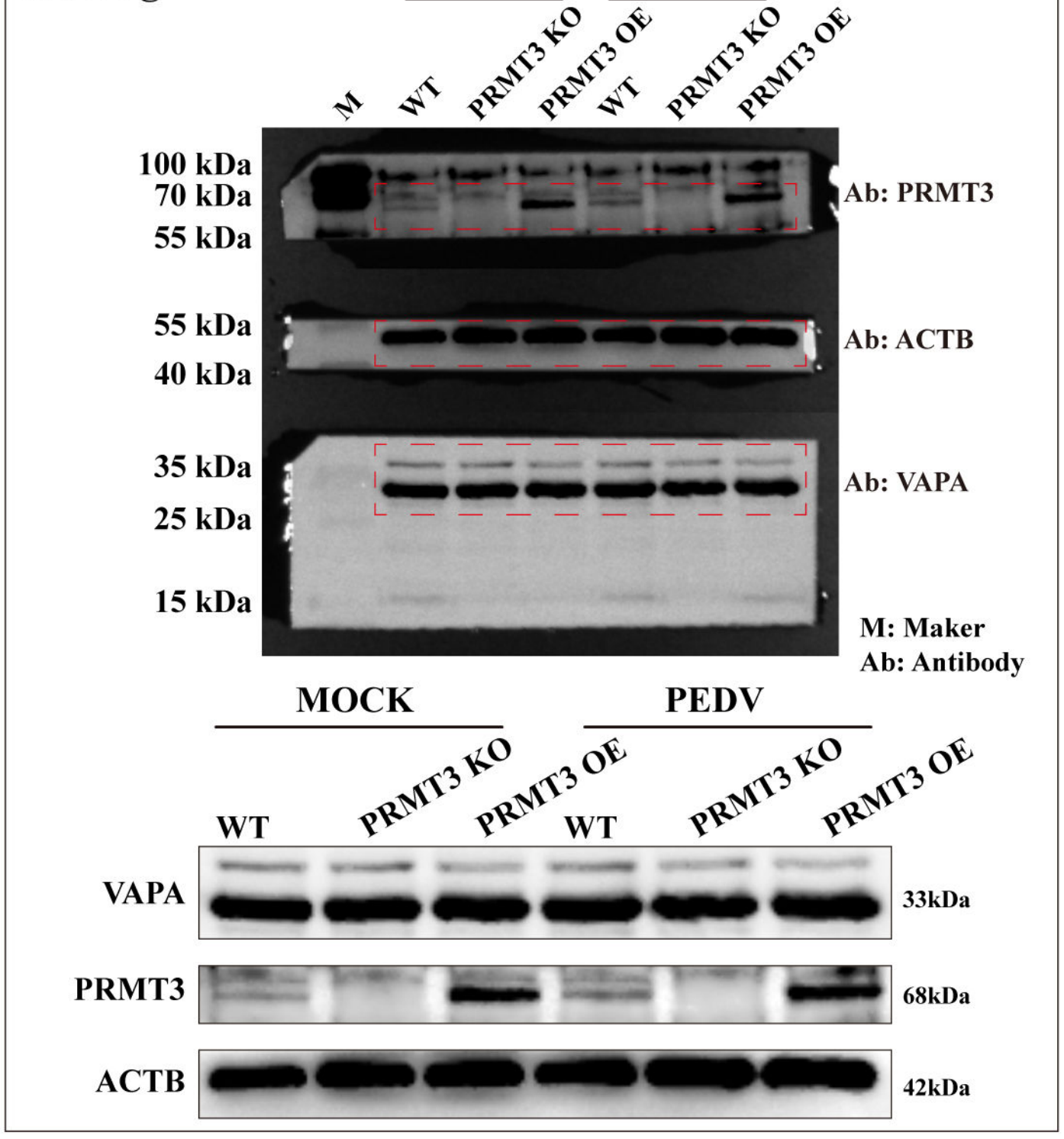

S2G Fig

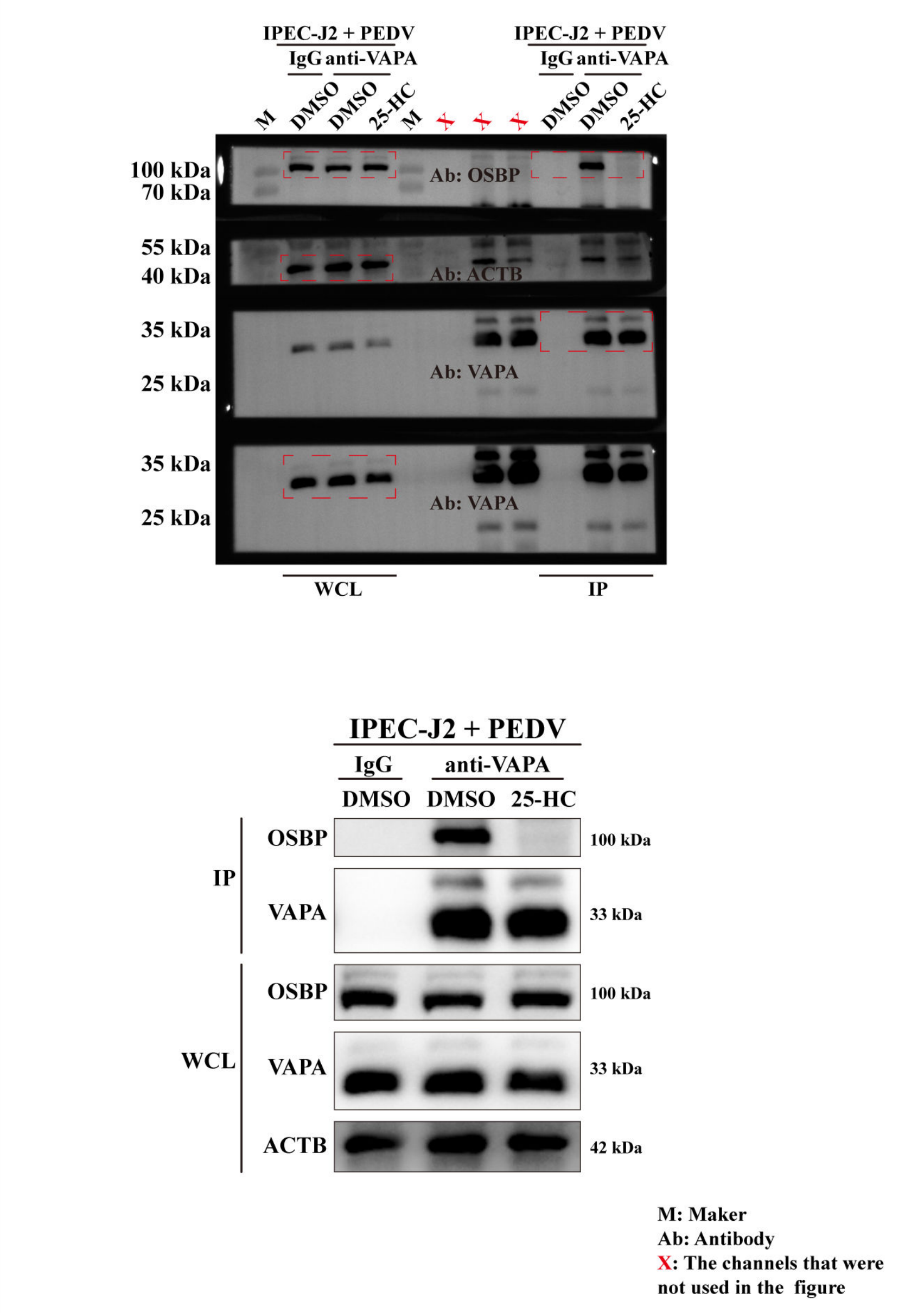

S2I Fig

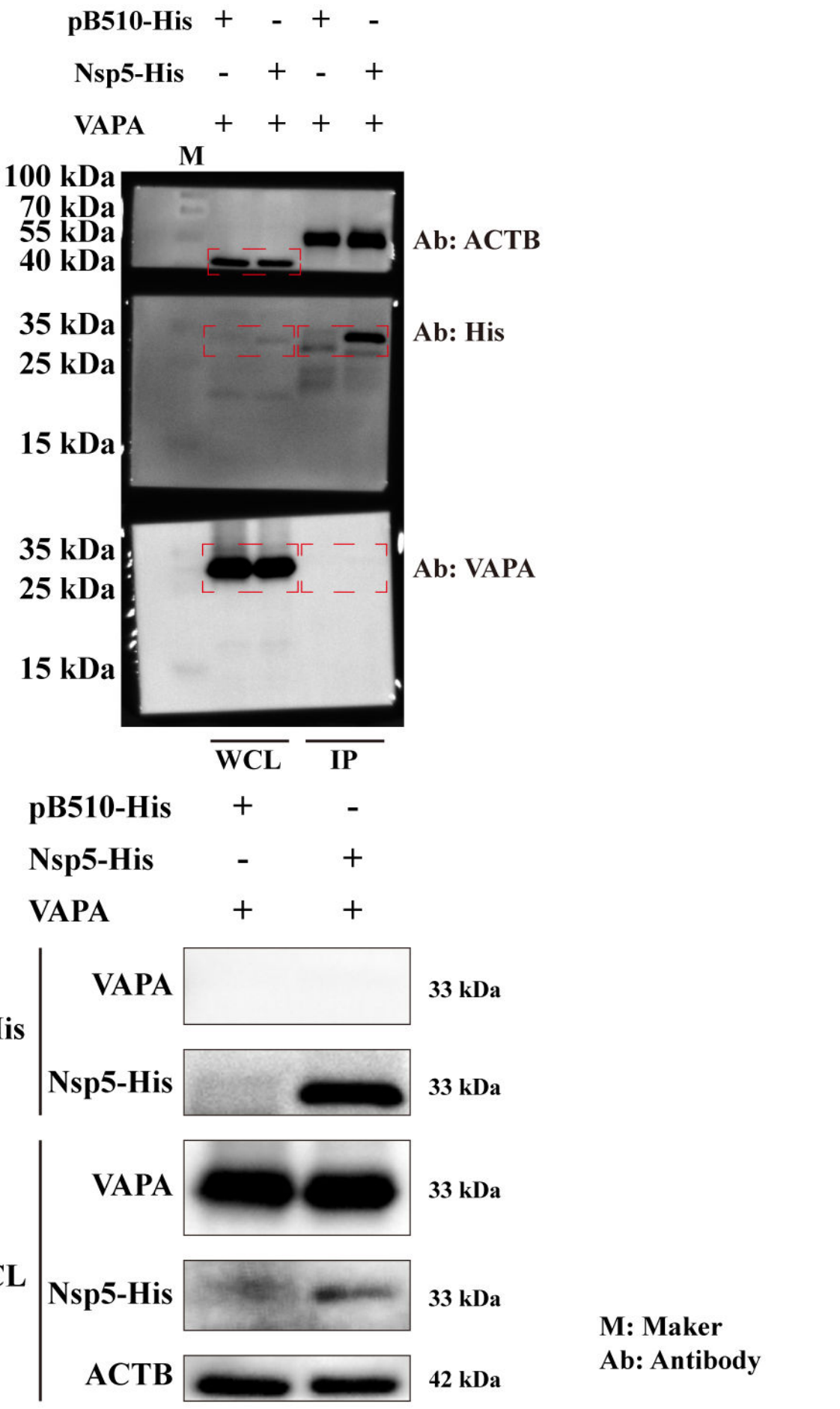

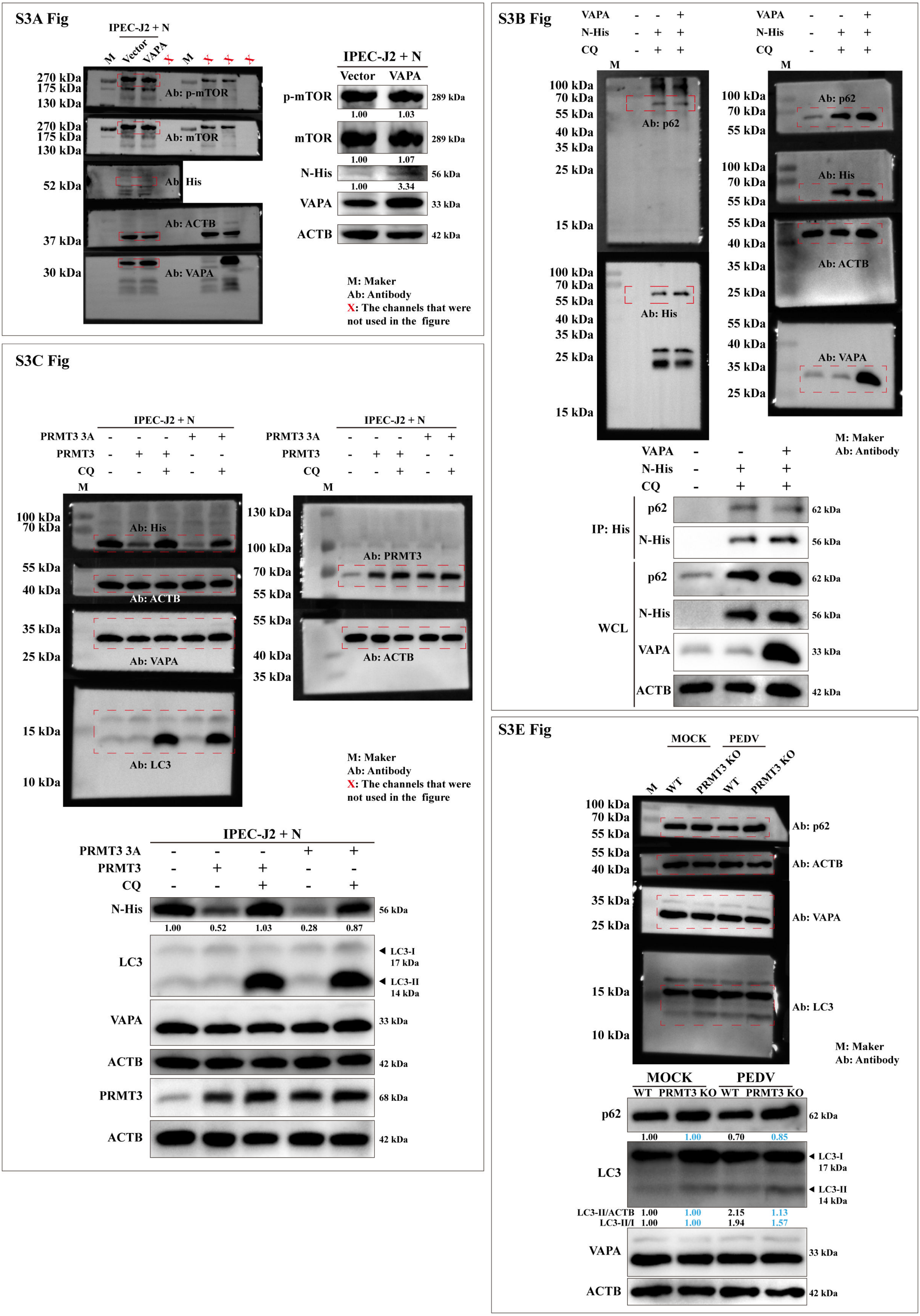
S3A Fig
IPEC-J2 + N
M Vector VAPA X M X X X
270 kDa
175 kDa
130 kDa
Ab: p-mTOR
270 kDa
175 kDa
130 kDa
Ab: mTOR
52 kDa
Ab: His
37 kDa
Ab: ACTB
30 kDa
Ab: VAPA
IPEC-J2 + N
Vector VAPA
p-mTOR 289 kDa
1.00 1.03
mTOR 289 kDa
1.00 1.07
N-His 56 kDa
1.00 3.34
VAPA 33 kDa
ACTB 42 kDa
M: Maker
Ab: Antibody
X: The channels that were not used in the figure
S3B Fig
VAPA - - +
N-His - + +
CQ - + +
M
100 kDa
70 kDa
55 kDa
40 kDa
35 kDa
25 kDa
15 kDa
Ab: p62
Ab: His
VAPA - - +
N-His - + +
CQ - + +
M
Ab: p62
Ab: His
Ab: ACTB
Ab: VAPA
M: Maker
Ab: Antibody
VAPA - - +
N-His - + +
CQ - + +
IP: His
p62 62 kDa
N-His 56 kDa
WCL
p62 62 kDa
N-His 56 kDa
VAPA 33 kDa
ACTB 42 kDa
S3C Fig
IPEC-J2 + N
PRMT3 3A - - - + +
PRMT3 - + + - -
CQ - - + - +
M
100 kDa
70 kDa
Ab: His
55 kDa
40 kDa
Ab: ACTB
35 kDa
25 kDa
Ab: VAPA
15 kDa
10 kDa
Ab: LC3
130 kDa
100 kDa
70 kDa
55 kDa
Ab: PRMT3
40 kDa
35 kDa
Ab: ACTB
M: Maker
Ab: Antibody
X: The channels that were not used in the figure
IPEC-J2 + N
PRMT3 3A - - - + +
PRMT3 - + + - -
CQ - - + - +
N-His 56 kDa
1.00 0.52 1.03 0.28 0.87
LC3
LC3-I 17 kDa
LC3-II 14 kDa
VAPA 33 kDa
ACTB 42 kDa
PRMT3 68 kDa
ACTB 42 kDa
S3E Fig
MOCK PEDV
M WT PRMT3 KO WT PRMT3 KO
100 kDa
70 kDa
55 kDa
Ab: p62
55 kDa
40 kDa
Ab: ACTB
35 kDa
25 kDa
Ab: VAPA
15 kDa
10 kDa
Ab: LC3
M: Maker
Ab: Antibody
MOCK PEDV
WT PRMT3 KO WT PRMT3 KO
p62 62 kDa
1.00 1.00 0.70 0.85
LC3
LC3-I 17 kDa
LC3-II 14 kDa
LC3-II/ACTB 1.00 1.00 2.15 1.13
LC3-II/I 1.00 1.00 1.94 1.57
VAPA 33 kDa
ACTB 42 kDa
